# Theory and Simulations of Ionic Liquids in Nanoconfinement

**DOI:** 10.1021/acs.chemrev.2c00728

**Published:** 2023-05-10

**Authors:** Svyatoslav Kondrat, Guang Feng, Fernando Bresme, Michael Urbakh, Alexei A. Kornyshev

**Affiliations:** † Institute of Physical Chemistry, Polish Academy of Sciences, 01-224 Warsaw, Poland; ‡ Institute for Computational Physics, University of Stuttgart, Stuttgart 70569, Germany; § State Key Laboratory of Coal Combustion, School of Energy and Power Engineering, Huazhong University of Science and Technology (HUST), Wuhan 430074, China; ∥ Nano Interface Centre for Energy, School of Energy and Power Engineering, Huazhong University of Science and Technology, Wuhan 430074, China; ⊥ Department of Chemistry, Molecular Sciences Research Hub, White City Campus, London W12 0BZ,United Kingdom; # Thomas Young Centre for Theory and Simulation of Materials, Imperial College London, South Kensington Campus, London SW7 2AZ, United Kingdom; ∇ London Centre for Nanotechnology, Imperial College London, London SW7 2AZ, United Kingdom; ○ School of Chemistry and the Sackler Center for Computational Molecular and Materials Science, Tel Aviv University, Tel Aviv 6997801, Israel

## Abstract

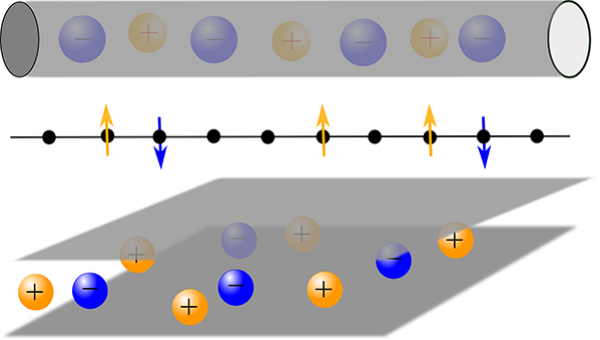

Room-temperature ionic liquids (RTILs) have exciting properties such as nonvolatility, large electrochemical windows, and remarkable variety, drawing much interest in energy storage, gating, electrocatalysis, tunable lubrication, and other applications. Confined RTILs appear in various situations, for instance, in pores of nanostructured electrodes of supercapacitors and batteries, as such electrodes increase the contact area with RTILs and enhance the total capacitance and stored energy, between crossed cylinders in surface force balance experiments, between a tip and a sample in atomic force microscopy, and between sliding surfaces in tribology experiments, where RTILs act as lubricants. The properties and functioning of RTILs in confinement, especially nanoconfinement, result in fascinating structural and dynamic phenomena, including layering, overscreening and crowding, nanoscale capillary freezing, quantized and electrotunable friction, and superionic state. This review offers a comprehensive analysis of the fundamental physical phenomena controlling the properties of such systems and the current state-of-the-art theoretical and simulation approaches developed for their description. We discuss these approaches sequentially by increasing atomistic complexity, paying particular attention to new physical phenomena emerging in nanoscale confinement. This review covers theoretical models, most of which are based on mapping the problems on pertinent statistical mechanics models with exact analytical solutions, allowing systematic analysis and new physical insights to develop more easily. We also describe a classical density functional theory, which offers a reliable and computationally inexpensive tool to account for some microscopic details and correlations that simplified models often fail to consider. Molecular simulations play a vital role in studying confined ionic liquids, enabling deep microscopic insights otherwise unavailable to researchers. We describe the basics of various simulation approaches and discuss their challenges and applicability to specific problems, focusing on RTIL structure in cylindrical and slit confinement and how it relates to friction and capacitive and dynamic properties of confined ions.

## 1 Introduction

The definition of the term ionic liquids (ILs) cannot be more straightforward: It is a liquid composed exclusively of cations and anions. In the past, we knew only high-temperature molten salts as an example of such liquids. Since the rediscovery of room temperature ionic liquids (RTILs) some 30 years ago, things have changed drastically.^1,2^ RTILs are electrolytes in which at least one ion type is a large organic ion of a complex shape and internal charge distribution; due to this complexity, RTILs do not freeze at room temperature, hence the name. The discovery of new moisture and temperature-stable RTILs boosted the interest in RTILs for practical applications.^3,4^ These later-generation RTILs attracted the broad attention of the electrochemical community^5−10^ as electrolytes possessing a unique combination of properties that made them exciting from fundamental and application point of view. These properties include high charge densities, electrochemical stability, low or nearly negligible volatility, tunable polarity, etc.

The variety of RTILs that can be synthesized is virtually unlimited. Moreover, they can mix in “cocktails of your choice” to acquire the desired properties, such as a wider temperature range of the liquid state and decreased viscosity. RTILs can serve not only as electrolytes in electrochemical applications but as almost “universal” solvents, often called “designer solvents”. Many RTILs can also mix with various organic and inorganic polar solvents, giving a practically infinite number of solvent-free or “solvent-doped” electrolytes and “solvent-in-salt” systems to achieve the target-oriented, tunable properties.^11,12^

An electrochemical window of RTILs is often significantly larger than of solutions of inorganic salts in organic solvents and aqueous electrolytes, reaching 3–4 V or even 7 V.^5,13−17^ A large electrochemical window is an additional attraction for electrochemists, as it allows one to subject an RTIL to higher voltages before the ions start reducing, oxidizing or decomposing, i.e., participating in electrode reactions. Indeed, many applications require an electrolyte to be electrochemically inactive, and RTILs can ensure their operation in a high-voltage regime. The wide range of such applications includes electrical double-layer capacitors (EDLCs), also called supercapacitors, electroactuators, reverse actuators, sensors, electrowetting lenses, and self-assembling optical metamaterials.

Many investigation techniques and real-world applications of RTILs rely on their behavior in confinement. For instance, RTILs are confined inside the pores of nanostructured electrodes in EDLCs; such electrodes increase the contact area with RTILs and enhance the total capacitance and stored energy. In atomic force microscopy (AFM), also known as scanning force microscopy (SFM), RTILs are located between the AFM tip and a sample; in the surface force apparatus (SFA), sometimes called surface force balance (SFB), RTILs are confined between the surfaces of crossed cylinders. In tribology experiments, RTILs are squeezed in nanogaps between sliding macroscopic surfaces that use RTILs as lubricants.

Of course, it may sound reasonable to assume that to understand the properties of RTILs in nanoconfinement, one needs to understand the structure and dynamics of bulk RTILs and the properties of semi-infinite systems with RTILs at a single surface. This statement seems logical and has direct value when the confinement dimensions are larger than the screening lengths and ion sizes of RTILs. However, the situation is different when the confinement becomes comparable to the ion size, which is the focus of this review. While a thin RTIL film may reflect some properties of the bulk or semi-infinite system, it generally has a different structure and properties and, as we shall see, exhibits a new and exciting physics. RTILs in such ultranarrow confinements require unique approaches to theory and computer simulations. The subject of this review is the description of such systems and their performance in different applications.

In 2006, Chmiola et al.^18^ and Raymundo-Piñero et al.^19^ observed that the capacitance of microporous electrodes increased with decreasing the (average) pore size down to the size of a desolvated ion. This observation challenged the traditional view that such small pores do not contribute to energy storage. However, similar trends have also been observed for solvent-free ILs,^20^ suggesting that the pore sizes comparable to the ion sizes provide the highest achievable capacitance. (We note that other experiments^21^ reported a constant capacitance in a wide range of pore sizes in the case of aqueous electrolytes. While the discussion of the origin of these discrepancies^21,22^ is beyond the scope of our review, we will mention a possible reason in section 5.2.3.) These experiments attracted much attention from theory and simulations aimed at rationalizing such an “anomalous increase of capacitance”. To explain this phenomenon, ref (23) introduced the concept of a superionic state, in which the interionic interactions inside a narrow conducting pore are screened by the charge carriers of the confining walls. The electrostatic screening is probably the most crucial in distinguishing narrow conducting confinements from nonconducting (e.g., dielectric) or wide (mesoscopic) ones. Herein, after briefly introducing definitions (section 2), we discuss the superionic state at length in section 3, pointing out how it differs for different confining geometries and materials.

Analytically tractable models are essential in physics and physical chemistry, as they allow the unveiling of generic features and more systematic development of new physical insights. Several models have been developed with exact or approximate (but reliable) analytical solutions to describe charge storage in ultranarrow confinements. Most of these models are defined on lattices and based on the concept of the superionic state. Probably the simplest one has been obtained by mapping the charge storage onto the celebrated Ising model of magnetism.^24^ In this case, cations and anions correspond to spin up and down, and the external magnetic field corresponds to the applied potential difference between the pore and the bulk electrolyte. In 1D, this model describes the charge storage in single-file pores such as carbon nanotubes (CNTs) or CNT forests.^25−27^ The beauty of this model is its simplicity and the analytical, closed-form solution known to us for nearly 100 years.^28^ In section 4, we describe the analytical models in detail, including single-file cylindrical (section 4.1) and single-layer slit confinements (section 4.2). We also discuss the difference between the lattice and continuum models (section 4.1.2) and use a continuum model to derive large-voltage asymptotic expressions for capacitance and energy storage that are not captured adequately by the lattice models (section 4.3). In addition, we describe how some of these models can be extended and formulated to describe the dynamics of charging (section 4.5).

Despite their beauty, analytical models often neglect vital microscopic details that may play an important role in extreme confinements. An essential step toward including such information is a so-called density functional theory (DFT) that has played an indispensable role in many areas of physics and physical chemistry.^29−34^ In section 5, we provide a brief historical introduction and technical account of DFT, including its dynamical version, a time-dependent DFT. Section 5.2 describes the applications of DFT and dynamical DFT to ionic liquids in narrow conducting confinements.

While DFT is formally exact, several approximations are necessary to perform the calculations in practice. These approximations often neglect fluctuations or approximate the correlation functions for confined ILs by their bulk values. Monte Carlo or molecular dynamics (MD) simulations naturally contain such fluctuations and provide direct access to experimentally measured quantities. In addition, MD simulations give information on nanopore ion dynamics, charging kinetics, and frictional properties of RTILs as lubricants, which are treated phenomenologically with analytical models and classical time-dependent DFT. In section 6.1, we introduce the readers to molecular simulations, explaining different simulation approaches, force fields and various challenges with simulation techniques. For instance, we discuss whether frequently used and computationally less demanding constant charge simulations adequately describe ionic liquids in narrow conducting confinements and how finite simulation boxes affect ion dynamics. These discussions are quite generic as similar issues arise also for bulk and semi-infinite ionic systems. We then proceed by describing the simulation results, focusing on ionic structure in slit and cylindrical confinements (section 6.2), capacitance and energy storage (section 6.3), and dynamical properties, such as ion diffusion, charge–discharge kinetics, and electrotunable friction (section 6.4).

When completing this review, we felt that despite the years of active research and the significant efforts of theorists and simulators, we still lack a clear understanding of many processes occurring in IL-filled narrow conducting confinements. In our final section (section 7), we provide concluding remarks by linking theory, simulations and experiments, pointing out a few open questions, and suggesting new perspectives and future directions, particularly concerning future experiments, that we feel are interesting and relevant.

After completing this work, we learned that two reviews on related subjects^35,36^ appeared in a special issue of *Chemical Reviews* on “Computational Electrochemistry”. Wu^36^ discussed the structure, capacitance, and dynamics of electrical double layers, and Jeanmairet et al.^35^ reviewed molecular dynamics simulations of electrical double layers (EDLs). Both articles touch on the issues discussed in our work; for instance, both discuss classical DFT, anomalous capacitance increase, and microstructure of real porous electrodes. While Jeanmairet et al. and Wu present a broad view on EDLs, including confined and nonconfined systems, we focus specifically on ionic liquids in nanoconfinement. Moreover, a unique feature of our review is that it presents the material by systematically increasing the model complexity accompanied by the incorporation of microscopic details of confined ionic systems. In doing so, we attempted to pay particular attention to physical phenomena described and predicted by simple analytical theories and discussed how they compare with simulations and experiments. Speaking of DFT and simulations, we devoted several pages to explain the mathematical details and the origin of classical DFT and the machinery of molecular simulations, discussing their benefits and challenges. Understanding these details is essential for researchers pursuing theory and simulations in this field and for experimentalists to know the intricacies and capabilities of the theory and simulations and, thus, their applicability to the description of experimental systems.

We thus welcome all readers to a journey into the world of nanoconfined ionic systems. We hope it will be as exciting to read this review as it has been for us to write it.

## 2 Some Basic Notions

### 2.1 Capacitance

An experimentally measurable quantity of special interest in electrochemistry is the differential capacitance^37^
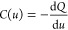
1where *Q* is the average electric charge accumulated in a nanopore (normalized to the surface area, volume, or mass of the electrode, if needed) and *u* is a potential difference applied to the electrode with respect to the bulk electrolyte. Frequently, the integral capacitance,
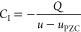
2where *u*_PZC_ is the potential of zero charge, is reported in the literature, particularly in molecular simulation studies. *C*_I_ is a useful quantity to characterize charge storage, but it does not provide new thermodynamic information because it is merely a ratio of two thermodynamic quantities. In contrast, the differential capacitance is a distinct thermodynamic quantity, describing the response of a system to the applied potential difference. It can be related to charge fluctuations^38^
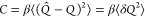
3where  is inverse temperature, *k*_B_ is the Boltzmann constant, *T* is absolute temperature, and  is the accumulated charge for microscopic configurations corresponding to a given macroscopic state. In Monte Carlo or MD simulations,  is the charge accumulated in a nanopore during each simulation step. The average accumulated charge , where ⟨·⟩ denotes thermal (ensemble) averaging over the microscopic configurations (see eq 15 below for an example).

In the linear regime, i.e., for *Q* ∼ *u*, the differential capacitance and the integral capacitance are equal, implying *Q*(*u*) = *C*_I_*u* = *Cu*.

We note that the capacitance defined by eqs 1 and 3 is an electrical double-layer capacitance due to the accumulated ionic charge in an electrode, assuming the electode is ideally metallic. For real electrodes, particularly for low dimensional electrode materials such as carbon nanotubes and graphene, there is an additional contribution to the total (measured) capacitance due to the finite density of states of electrons in such electrodes. We briefly discuss the role of quantum capacitance in section 4.4.

### 2.2 Stored Energy

A practically important quantity is the electrical energy stored in a nanopore
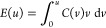
4For a constant capacitance, the stored energy is
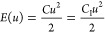
5We stress that eq 5 applies only in the linear regime and cannot be used in the case of a voltage-dependent capacitance; eq 4 must be used instead.

### 2.3 Mechanisms of Nanopore Charging

**Figure 1:**
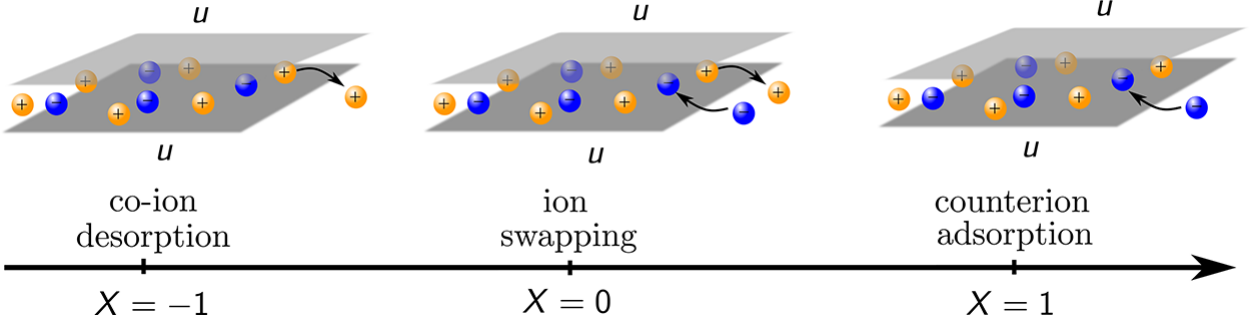
Three basic mechanisms of nanopore charging. Schematics of co-ion desorption (*X* = −1), co-ions swapping for counterions (*X* = 0), and counterion adsorption (*X* = 1). *u* is the potential difference applied to the nanopore with respect to bulk electrolyte (not shown). The differential charging parameter *X*(*u*) is defined by eqs 7. A charging mechanism can be a combination of swapping and either adsorption (*X* > 0) or desorption (*X* < 0). Note that *X* can be smaller than −1 (larger than +1), in which case co-ion desorption (counterion adsorption) is accompanied by the desorption of counterions (adsorption of co-ions).

A nonpolarized nanopore (*u* = 0) can be empty or filled with ions, depending on the pore ionophilicity. An empty or almost empty pore inevitably charges by adsorbing counterions, i.e., its charging mechanism is counterion electrosorption (or simply adsorption). For a pore that is not empty at zero voltage, two additional charging mechanisms can take place: (i) co-ion desorption and (ii) swapping of co-ions for counterions from the bulk electrolyte (Figure 1). The ion swapping mechanism operates when the rate of adsorption and desorption are equal. In practice, the charging mechanism is a combination of swapping and either adsorption or desorption.

To identify these charging mechanisms, Forse et al.^39^ introduced the charging parameter
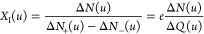
6where Δ*N* = *N*(*u*) – *N*(0) is the change in the total number of ions in the pore and Δ*N*_±_ = *N*_±_(*u*) – *N*_±_(0) is the change in the number of cations and anions at an applied potential difference *u* compared to the uncharged pore. The last expression in eq 6 applies to monovalent ions.

For an exact one-to-one exchange of co-ions with counterions, one has Δ*N* = 0 and, hence, the charging parameter *X*_I_ is zero. When the charging is driven by pure electrosorption, as, e.g., for ionophobic pores, then Δ*Q* = *e*Δ*N* and *X*_I_ = 1, while for pure co-ion desorption, Δ*Q* = −*e*Δ*N* and hence *X*_I_ = −1. The charging parameter can also be larger than unity or smaller than −1. *X*_I_ > 1 means that co-ions are adsorbed into a pore along with counterions, while *X*_I_ < −1 implies that the counterions are expelled from a pore together with the co-ions. Such charging is rare; however, it occurs, e.g., in the case of phase transitions between ordered and disordered ionic liquid phases as predicted by two-dimensional lattice models,^40^ mimicking ions in ultra narrow slit confinements (section 4.2.2). Recently, Bi and Salanne^41^ reported with molecular dynamics simulations that desorption (*X* < 0) is the primary mechanism of charging pores formed by layered metallic molybdenum disulfide (1T-MoS_2_), which they attributed to the high electronegativity of sulfide anion.

Breitsprecher et al.^42^ introduced a differential version of *X*_*I*_
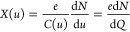
7aSimilarly to *X*_I_, the value *X* = 0 corresponds to swapping and *X* = ±1 to pure adsorption/desorption. Anagolously to the differential capacitance, *X*(*u*) characterizes the thermodynamic state of a system at an applied potential *u* and can be obtained from charge-density fluctuations^43^
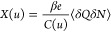
7bwhere , with  being the total number of ions in the pore for microscopic configurations corresponding to a given thermodynamic state and  the average number of ions.

In the presence of solvent, one can introduce an additional parameter^44^
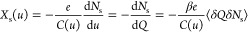
8where  with  being the average number of solvent molecules in the nanopore and  the number of solvent molecules in microscopic configurations corresponding to a given thermodynamic state. This parameter quantifies the contribution of the solvent to the charging process due to excluding or freeing the volume for ion adsorption or desorption. For instance, for the charging parameter *X* = 1, the value *X*_s_ = 1 indicates that the charging is determined fully by ion–solvent swapping.

## 3 Superionic State in Conducting Nanoconfinement

### 3.1 Screening of Electrostatic Interactions

**Figure 2:**
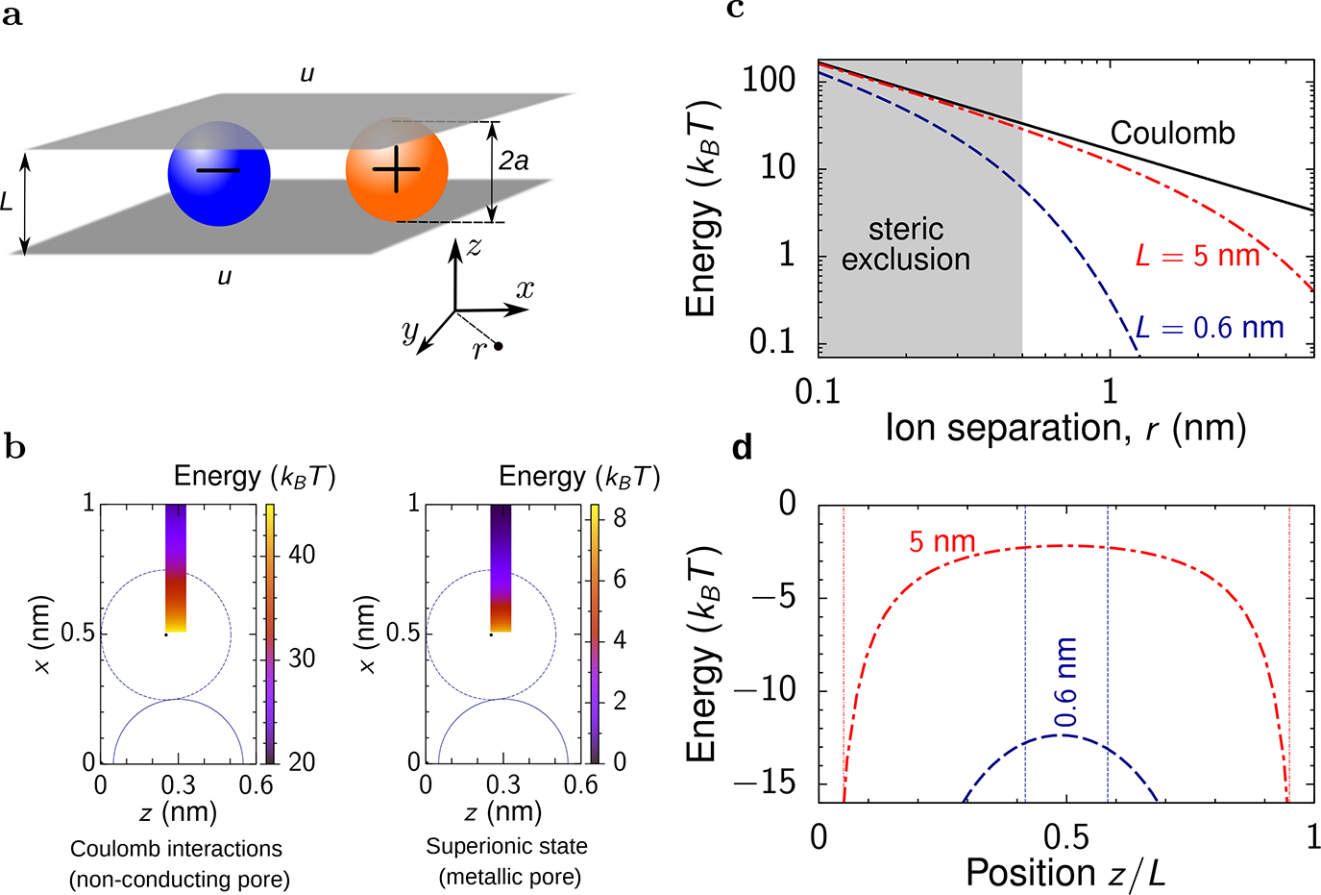
Superionic state in slit nanopores. (a) Model of a slit-shaped metallic pore. The slit width *L* is comparable to the ion diameter *a* = *a*_±_. A potential difference *u* is applied to the pore walls with respect to bulk electrolyte (not shown). (b) Absolute value of the electrostatic interaction energy between two ions in a nonconducting and metallic slit. The circles exemplify the locations of the ions. The interactions energy is shown only in the regions allowed by steric exclusion. Ion diameter 2*a* = 0.5 nm and the pore width *L* = 0.6 nm. (c) Absolute value of the Coulomb interaction energy and the interionic interactions in the superionic state for two slit widths. The ions reside on the symmetry plane of the slit, and *r* is the distance between them. The interaction energy is given by eq 9. The shaded area shows the region of steric exclusion. (d) Effective image-charge ion-pore wall attraction given by eq 12, shown for two indicated pore widths The thin vertical lines show the locations of the center of an ion that are closest to the pore walls. Note that the are four vertical lines, two for each slit width; the pore walls are at *z* = 0 and *z*/*L* = 1. In all plots, the in-pore relative permittivity ε = 2.5 and temperature *T* = 293 K.

A superionic state means the reduction of interionic interactions under conducting confinement. To demonstrate the superionic state, we consider two point charges α and γ (α, γ = {+,−}), in a slit-shaped, charged nanopore formed by two infinitely extended, perfectly metallic walls (Figure 2a). Assuming, for simplicity, that the charges reside on the symmetry plane of the slit, the electrostatic interaction energy between them is (in SI units)^23^
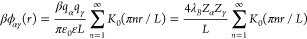
9where *q*_α_ and *q*_γ_ denote the ion charges and *Z*_α_ = *q*_α_/*e* and *Z*_γ_ = *q*_γ_/*e* their valencies, *K*_0_(*x*) is the zero-order modified Bessel function of the second kind, *L* is the slit width, and ε_0_ is the vacuum permittivity. The relative permittivity inside the pore, ε, is determined by the electronic and rotational degrees of freedom of ions (and due to a solvent if present) and is reduced compared to the bulk relative permittivity. The value of ε is not precisely known, but one might expect it to vary between 2 for simple ions and 5 or more for more bulky ions or in the presence of a solvent.^23^ Furthermore, we note that the dielectric response in the directions lateral and perpendicular to the pore walls differ for narrow confinements. For water as a solvent, this asymmetry becomes particularly pronounced for nanopores below 1 nm.^45−47^ Schlaich et al. showed with MD simulations that for a slit nanopore, the lateral component of the dielectric response slightly increased over the bulk value, while the perpendicular component drastically decreased due to anticorrelated polarization of neighboring water dipoles.^45^

To avoid complications with different systems of units, it is convenient to write the interaction potentials in dimensionless form using the Bjerrum length (see the last expression in eq 9). The Bjerrum length λ_B_ = β*e*^2^/(4πε_0_ε) (or λ_B_ = β*e*^2^/ε in Gaussian units) is the distance between two elementary charges at which their Coulomb energy is equal to the thermal energy (= *k*_B_*T*). For typical ionic liquids in bulk, the Bjerrum length is of the order of a few nanometres, while λ_B_ ≈ 0.7 nm for aqueous electrolytes at room temperature. Because ε in confinement differs from its value in bulk, one gets, using the values of ε mentioned above, that the Bjerrum length varies from about 10 to 25 nm.

For large ion–ion separations, *r* ≫ *L*, eq 9, reduces to
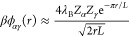
10

Equation 10 shows that the electrostatic interaction energy between two ions in a narrow metallic slit is exponentially screened with the decay length determined by the distance between the plates divided by π (the factor π is important as it reduces the decay length). This screening arises from the response of the conduction electrons (and holes) to the presence of the ions. The resulting interaction energy differs from that between charges confined between nonconducting walls, where the ionic interactions are Coulombic. Figure 2b,c compares the Coulomb interaction potential with the interaction potential given by eq 9. Taking a typical ion diameter 2*a* = 0.5 nm, the slit width *L* = 0.6 nm, and using ε = 2.5 (corresponding to λ_B_ ≈ 22 nm at room temperature), the interaction energy at contact is ϕ(2*a*) ≈ 8*k*_B_*T*. The corresponding unscreened interaction energy is ϕ_C_(2*a*) ≈ 45*k*_B_*T*, i.e., the ionic interactions in a narrow metallic slit are a few times reduced. For instance, if we double the distance between the ions, the electrostatic interactions energy becomes , while the Coulomb energy ϕ_C_(4*a*) = ϕ_C_(2*a*)/2 ≈ 22.5*k*_B_*T*. This estimate suggests that the interactions between next-nearest ions in a metallic slit can be neglected, at least in the first approximation, motivating several analytically solvable models for charge storage in ultranarrow pores (section 4). Such strong screening of interionic interactions allows easier unbinding of “ion pairs”^48^ and tighter packing of ions of the same sign. This effect has been used^49^ for rationalizing the anomalous increase of capacitance in ultranarrow pores^18−20^ (cf. Figure 5).

Screening of interionic interactions occurs also in other pore geometries and for nonideal metallic walls.^50^ In particular, for two ions residing on the symmetry axes of a a cylindrical pore inside a metal, the electrostatic interaction energy is^24,50^
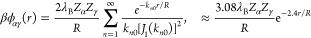
11where *R* is the pore radius, *J*_*n*_ is the Bessel function of the first kind and *k*_*n*0_ is the *n*th positive root of *J*_0_. The last expression in eq 11 is for large ion–ion separations (*r* ≫ *R*) and shows that, again, the interactions are exponentially screened. Note that for a cylinder, the screening length (*R*/2.4) is shorter than in a slit (*L*/π = 2*R*/π, eq 9) because the cylinder provides a stronger confinement. This observation is consistent with the results of molecular dynamics simulations, showing that highly confined ions store electrical energy more efficiently.^51^

Goduljan et al.^52^ used quantum density functional theory (qDFT) to investigate the screening in carbon and gold nanotubes. They demonstrated that the ionic interactions in both nanotubes are indeed strongly reduced, but the effect is more substantial in the gold nanotube due to its metallic nature. Their calculations suggested that eq 11 provides a reliable approximation also for carbon nanotubes; for a gold nanotube, the effective value of *R* fitting eq 11 to the qDFT calculations was slightly smaller than the physical pore radius, likely due to a quantum spillover of the electronic cloud from the pore wall to the pore interior.

### 3.2 Image-Charge Attraction between an Ion and the Pore Walls

In addition to weakening the ion–ion interactions, the interaction of ions with the conducting electrons of a confining solid leads to an effective attraction between the ions and the pore. Such attraction arises due to charge–image charge interactions, which are also present at flat electrodes but are amplified due to confinement. In the case of a slit confinement, the image–charge interaction energy is
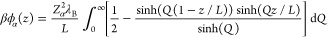
12where *z* is the position across the slit. This potential is plotted in Figure 2d for two values of slit width *L*, showing that it is negative and decreases as an ion approaches the pore wall. It means that it is favorable for an ion to reside inside a pore and to be as close to the pore wall as possible. Note that ϕ_α_ diverges for *z* → 0 and *z* → *L*, hence accounting for hard-core interactions between an ion and the pore wall is essential to prevent these divergences. In the middle of a slit, eq 12 reduces to ϕ_α_(*z* = *L*/2) = −*Z*_α_^2^λ_B_ ln(2)/*L*, hence ϕ_α_ decays to zero with increasing the slit width as 1/*L*.

Goduljan et al. studied the insertion of alkali ions into carbon and gold nanotubes and estimated that the image–charge attraction could well compensate for the loss of the solvation shell.^52−54^ Again, the effect was stronger for gold nanotubes. Interestingly, they found that the most favorable position for a sodium ion was at the pore wall, as predicted by eq 12 (Figure 2d), but a chloride ion preferred to be at the nanotube center, unlike predicted by the image–charge attraction; this result is likely due to nonelectrostatic interactions.^53^

## 4 Analytical Models

While a superionic state emerges in any conducting nanoconfinement, analytical expressions for interionic interactions can be obtained only for a few simple geometries. Particularly important are metallic cylinders and slits (eqs 11 and 9), which motivated several one-dimensional (1D) and two-dimensional (2D) analytical models for nanopore charging. Such models are valuable to develop new physical insights and provide grounds for in-depth analyses of the charging behavior at the nanoscale.

### 4.1 Single-File Cylindrical Pores

We start by analyzing the charge storage in single-file pores. While such pores are kinetically problematic because ions cannot swap inside the pore (but see section 4.5.2), they can be mapped onto known 1D problems of statistical physics that often have exact analytical solutions.

#### 4.1.1 Lattice Models

Such models are popular in studying physical and physicochemical problems, as they are easier to deal with in simulations and often offer analytical solutions. Lattice models have been used to investigate ionic fluids in bulk^55^ and between two flat electrodes,^56−59^ in part inspiring the work on confined ionic liquids.^24,60^

**Figure 3:**
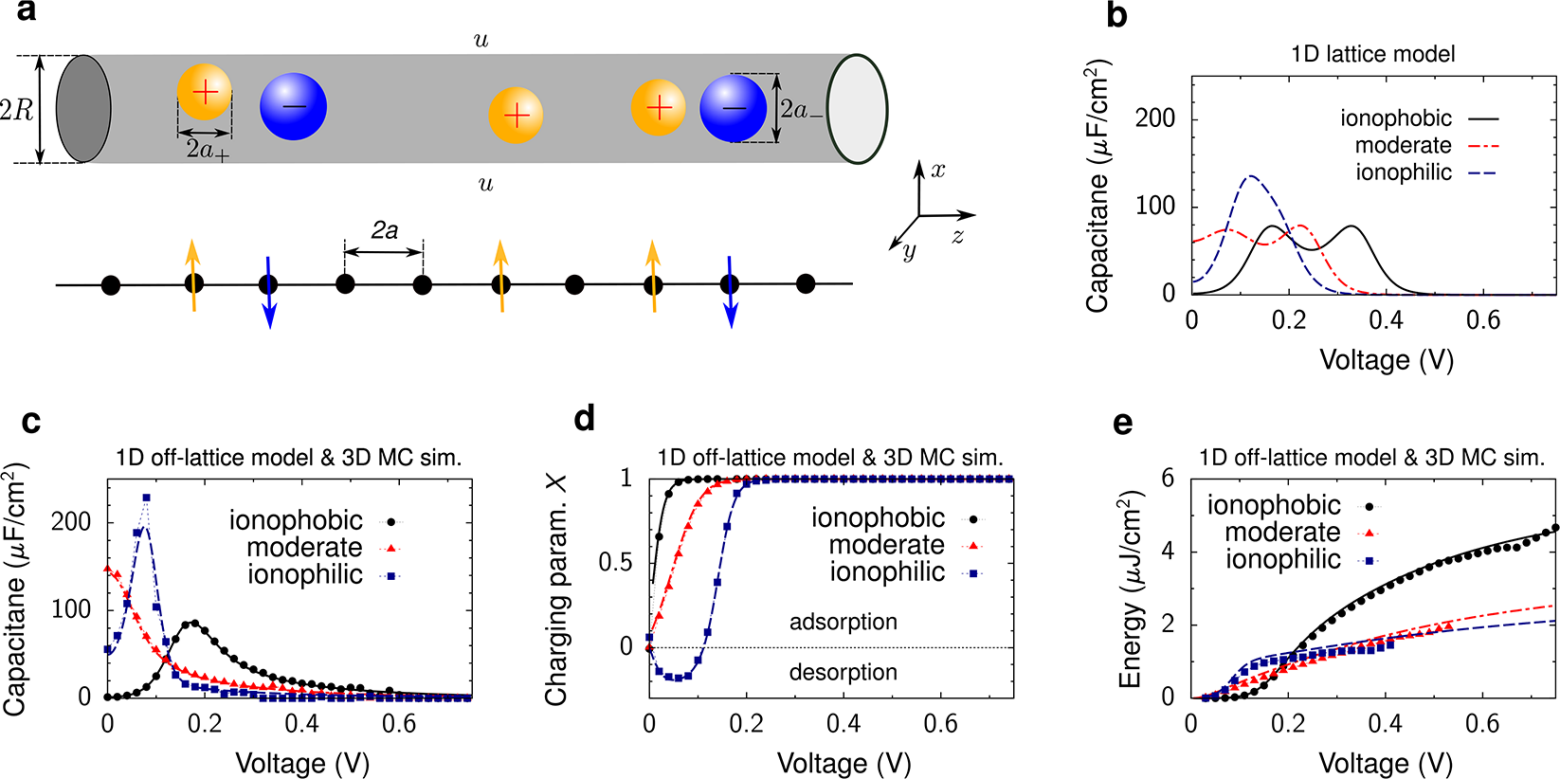
Charge storage in single-file pores. (a) Model of a metallic cylindrical nanopore of radius *R*. An electrostatic potential difference *u* is applied to the nanopore with respect to bulk electrolyte (not shown). The ion radii are *a* = *a*_±_ < *R*. In 1D lattice and off-lattice models, the centers of ions and solvent molecules (if any) are located on the symmetry axis of the nanotube. In the lattice model, the ions are located on the lattice sites. The lower cartoon shows the lattice model with the arrows symbolizing the spins oriented up or down, corresponding to the cations and anions in the nanotube shown above. (b) Results of 1D spin–lattice mode given by eq 18 (lower cartoon in (a)). Capacitance is shown as a function of applied potential difference for strongly ionophilic (chemical potential μ_IL_^(lat)^ = −0.961 eV) and ionophobic pores , and for a pore moderately filled with ions . (c–e) Results of 1D off-lattice model (lines) and 3D MC simulations (symbols). (c) Capacitance, (d) charging parameter *X*, 7, and (e) stored energy density are shown as functions of voltage. Parameter *X* > 0 (*X* < 0) corresponds to the charging driven by a combination of ion swapping and counterion adsorption (co-ion desorption). The chemical potentials were adjusted so as to provide the same in-pore ion densities as the lattice model in panel (b): μ_IL_ = −0.8 eV and μ_IL_ = −1.1 eV for the ionophilic and ionophobic pores, and μ_IL_ = −0.95 eV for the moderately filled pore. The plots have been created using the data from ref (44). In all plots, the ion radius *a* = 0.25 nm, the pore radius *R* = 0.26 nm, the in-pore dielectric constant ε = 2.5, and temperature *T* = 293 K.

Perhaps the simplest lattice model for ions in strong confinement is a one-dimensional “spin” model, as first introduced in ref (24). In this model, a classical spin *S*_*i*_ at site *i* can take values +1 or −1, corresponding to a cation or anion located inside a single-file pore at position *z* = *i* × *d*, where *d*(≈2*a*) is the lattice constant (Figure 3a). The Hamiltonian for this model is
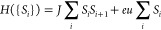
13where *u* is the applied potential measured with respect to bulk electrolyte and *J* = ϕ_++_(*d*) is the coupling constant, with ϕ_++_(*z*) given by eq 11. Note that eq 13 assumes that only ions from the neighboring sites interact. This assumption seems reasonable due to the exponential screening of interionic interactions (eq 11).

The Hamiltonian in eq 13 is the celebrated Ising model,^28^ well-known in the theory of magnetism (note that *h* = *eu* plays the role of an external magnetic field in magnetism). The partition function for the Ising model is
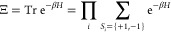
14and the expectation value of an observable *A* is obtained via thermal (ensemble) averaging
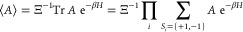
15Due to the assumption of vanishing interactions between next-nearest and higher-order neighbors, the partition function and hence all thermodynamic properties of the model can be computed analytically using a 2 × 2 transfer matrix.^61^ In particular, the accumulated charge, which is proportional to an average spin ⟨*S*⟩, is
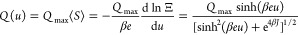
16where *Q*_max_ = *e*/(4π*Ra*) is the maximum accumulated charge per surface area (for monovalent ions). The differential capacitance can be computed from *Q*(*u*) or from charge fluctuations (spin response function)
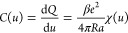
17awhere
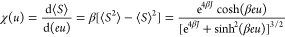
17bis the response function.^61^ This model predicts that the zero-voltage capacitance is nonzero (and finite), exhibits a peak and decays exponentially to zero for increasing voltage (see the ionophilic pore in Figure 3b). Note the differences between the differential capacitance, *C*(*u*) given by eqs 17, and the integral capacitance *C*_I_(*u*) = *Q*(*u*)/(*u*-*u*_PZC_) with *Q*(*u*) given by eq 16.

The Hamiltonian defined by eq 13 has been extended to take into account solvent effects,^60^ size-asymmetric ions,^62^ and multifile nanopores.^63^ In particular, introducing spin zero, *S*_*i*_ = 0, to describe solvent (or void), one can write for the Hamiltonian^60^
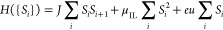
18where μ_IL_ is the ion chemical potential, which controls the in-pore ion density at zero polarization (*u* = 0). We included in μ_IL_ the image–charge energy and the energy of transfer of an ion from a pore to bulk electrolyte (i.e., outside of the pore). The Ising model, eq 13, corresponds to the limit of μ_IL_ → *∞*, that is, to the case of strongly ionophilic pores, fully occupied by ions at any applied potential.

The Hamiltonian defined by eq 18 represents the Blume–Capel (BC) model of magnetism.^64,65^ Similarly to the Ising model, the BC model can be solved exactly in 1D using the transfer matrix formalism, however, the solution is much lengthier.^60^Figure 3b shows the capacitance versus voltage for three values of μ_IL_, corresponding to ionophilic (≈ Ising model), moderately filled and ionophobic pores. There is a transition from one-peak to two-peak capacitance as the pore ionophobicity increases. For ionophobic pores, the first peak appears when the system overcomes an energy barrier for ions to enter a pore; clearly, this peak is absent for pores filled with ions at zero polarization. The second peak emerges when the system overcomes the repulsion between the counterions as their density increases. Such a two-peak capacitance can be related to a particle–hole duality (i.e., the formal correspondence between an ion and void) of the lattice model emergent in the limit of strong ionophobicity.^60^

#### 4.1.2 Off-Lattice Model

In recent work, Verkholyak et al.^44^ have proposed an off-lattice model based on the exact solution of a 1D multicomponent mixture of interacting particles.^66−68^ In this model, the ions’ positions are not restricted to lattice sites, but the ions can interact only with their nearest neighbors. This assumption allows applying the transfer-matrix approach to compute the partition function analytically, conceptually similar to the lattice models. The analytical solution exists in a parametric form,^44^ but it is lengthy and is not presented here.

Figure 3c shows the capacitance of ionophobic, moderately filled, and ionophilic pores obtained by using the off-lattice model. This figure demonstrates a remarkable agreement of the analytical results with 3D Monte Carlo simulations (section 6.1.1). Unlike the lattice model, the off-lattice model and MC simulations predict a single peak in the capacitance, either at zero voltage (bell-shaped capacitance) or at a nonzero voltage (camel-shaped capacitance). For the ionophobic pore, the second peak at a larger voltage, predicted by the lattice model, is lacking due to the absence of a particle-hole duality in continuous approaches.

The off-lattice model predicts a transformation from a camel-shaped capacitance to a bell-shaped capacitance as the pore ionophilicity increases. Such a transformation also occurs at flat electrodes.^69^ Perhaps surprisingly, for strongly ionophilic pores, which are nearly fully occupied by ions, the capacitance again becomes camel-shaped. This behavior could be related to co-ion desorption (charging parameter *X* < 0, see Figure 3d), which is unlikely to occur at flat electrodes.^44^ We note that both lattice and off-lattice models overestimate the capacitance, predicting strong peaks reaching hundreds of μF/cm^2^, about an order of magnitude higher than the measured values (see, e.g., Figure 5). Apart from the numerous simplifications enabling analytical solutions, which affect the comparison with experiments, several studies showed that the distribution of pore sizes could reduce the capacitance to the values comparable to those obtained in experiments.^24,70,71^

The stored energy can be computed from the differential capacitance using eq 4. Figure 3e shows that at large voltages, the energy stored in the ionophobic pore is more than two times larger than the energy stored in the ionophilic pores. This is because ionophobic pores extend the region of “active” charging (i.e., nonvanishing capacitance) to higher voltages, leading to higher stored energies (see eq 4).^60,71^

The off-lattice model has been recently used to study the effect of quantum capacitance on energy storage^72^ and optimize nanoporous electrodes for waste heat to energy conversion.^73^

#### 4.1.3 Schmickler’s Model

An interesting model has been proposed by Schmickler,^74^ who considered ions of such different sizes that only one ion type (counterions) could enter a pore. Schmickler proposed the following phenomenological expression for free energy
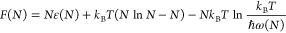
19where *N* is the number of counterions,  is the density-dependent electrostatic energy ( is the pore length) and  is the frequency of ions’ oscillations around their equilibrium positions determined by the interaction energy (*ℏ* is the Planck constant divided by 2π). In eq 19, the first term is the electrostatic energy, the second term is the entropic contribution and the last term is the contribution due to ion vibrations (oscillations) along the nanopore axis. For the interaction energy ϕ(*r*), Schmickler used the results of quantum density functional calculations,^52,53^ consistent with the superionic state (section 3). Thus, the Schmickler model is an improved mean-field model, which considers lateral oscillations of ions around their equilibrium positions, in addition to the classical mean-field description.

**Figure 4:**
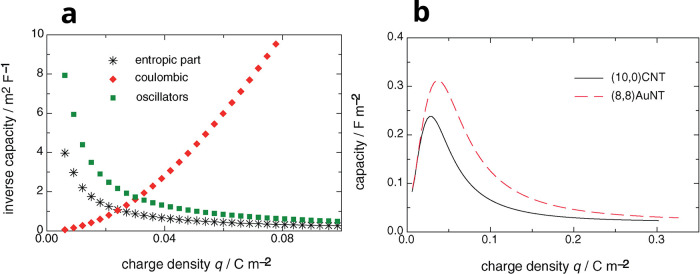
Model of Schmickler for single-file pores accommodating only one type of ions. (a) Entropic, Coulombic, and oscillation contributions to inverse capacitance predicted by the model given by eq 19. (b) Capacitance as a function of accumulated charge for carbon and gold nanotubes. Reproduced with permission from ref (74). Copyright 2015 Elsevier.

Examples of the three contributions to the inverse capacitance are shown in Figure 4a for a (10,0) carbon nanotube as a function of the (accumulated) charge density *q* (∼*N*). The entropic and oscillation contributions diverge as *q* → 0 and the Coulomb part vanishes because of the vanishing number of particles in the tube.

Figure 4b shows the differential capacitance for carbon and gold nanotubes. In both cases, the capacitance has a peak and decays to zero at high pore fillings, corresponding to high voltages, similarly to the Ising model and off-lattice results (Figure 3). Note that the capacitance in Figure 4b is shown as a function of the accumulated charge rather than applied potential difference.

For short tubes, the Schmickler model shows that the charging proceeds in steps multiple of the elementary charge (not shown here), similarly to quantum dots.^74^ Such quantized charging should, in principle, be observed for any sufficiently short (a few ion diameters long) single-file pore.

### 4.2 Single-Layer Slit-Shaped Pores

Similarly to 1D models applied to single-file pores, the charging of ultranarrow slit-pores, accommodating one layer of ions, can be studied by 2D models. Clearly, the 1D lattice models can be straightforwardly extended to two dimensions. We start, however, from a continuum model, first introduced^23^ to explain an anomalous increase of capacitance measured for subnanometre pores.^18−20^

#### 4.2.1 Mean-Field Continuum Model

Assuming that the ions reside on the symmetry plane of a slit, one can derive the following equation for the free energy density of ions in a 2D confinement^23^
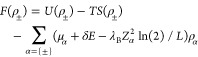
20where ρ_±_ are 2D ion densities, μ_±_ = μ_IL_ ± *eu* are the electrochemical potentials of ions, with *u* being the voltage applied to a slit with respect to bulk electrolyte; δ*E* is the resolvation energy describing (partial) loss of the solvation shell when an ion is transferred from the bulk electrolyte into a pore, and the third term in the parentheses comes from the image–charge attraction (see eq 12 and below it).

For a 2D system of ions, ref (23) used a 2D lattice–gas entropy
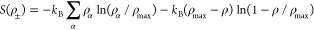
21where ρ = ρ_+_ + ρ_–_ is the total ion density and ρ_max_ = 3*L*η_max_/(4π*a*^3^) the maximum density, with η_max_ being the maximum packing fraction (η_max_ ≈ 0.64 for random close packing^75^). The internal energy was approximated by^23^
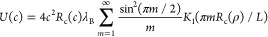
22where *c* = ρ_+_ – ρ_–_ is the charge density (in units of the proton charge) and *K*_1_(*x*) is the first-order modified Bessel function of the second kind. This expression was derived by calculating the electrostatic energy of ions by using eq 9 for the interionic interaction potential ϕ_αγ_(*r*) and applying the cut-out disk approximation with the density-dependent cut-out radius *R*_c_(ρ) = (πρ)^1/2^. eq 22 can be obtained from classical density functional theory (cf. section 5.1.1) in two dimensions by setting for the direct correlation function .

**Figure 5:**
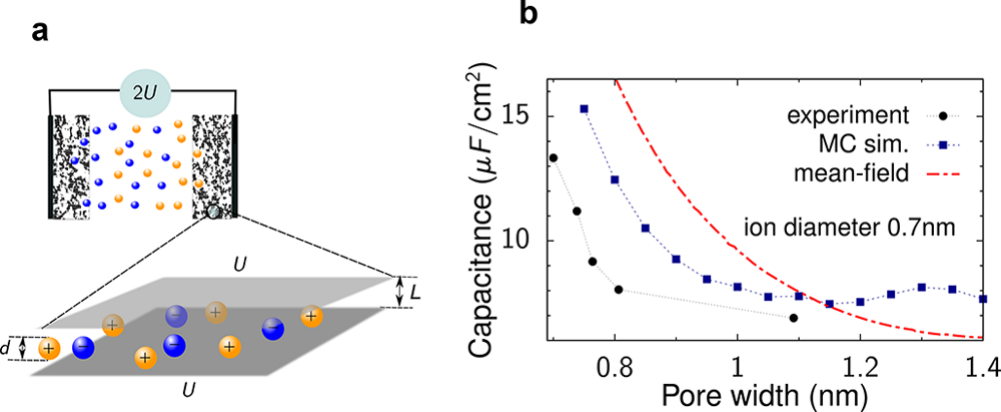
Anomalous capacitance and mean-field model. (a) Model of a slit-shaped metallic pore as a part of a supercapacitor’s electrode. Slit width *L* is comparable to the ion diameter 2*a* = 2*a*_±_. Potential difference *u* is applied to the pore walls with respect to bulk electrolyte. (b) Zero-voltage capacitance obtained by the mean-field model, eq 20, qualitatively agrees with Monte Carlo simulations^49^ (see section 6) and experiments:^20^ While the mean-field model overestimates the capacitance, it captures the anomalous capacitance increase. The plot has been created using the data from refs (20), (23), and (49).

This model is highly nonlinear, and its analytical solution is not known. Nevertheless, it can be straightforwardly analyzed numerically. Figure 5 shows that the capacitance (taken at zero polarization) increases with decreasing the slit width. This is in line with experiments^18−20^ and Monte Carlo simulations of the same model^49^ (see section 6.1.1). Although the mean-field theory overestimates the capacitance values, it captures the general trends well. Equations 20–22 have been applied to study transitions between co-ion rich and co-ion deficient phases^23,76^ (section 4.2.3) and charging mechanisms.^71^

#### 4.2.2 Lattice Model

**Figure 6:**
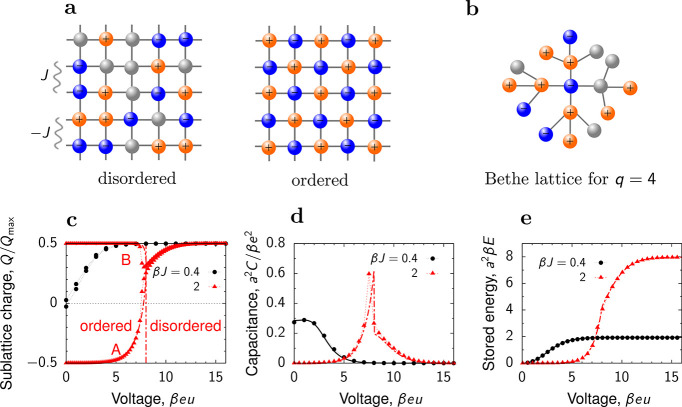
Lattice model for slit confinements. (a) Ions on a square lattice in a quasi 2D slit confinement. The model given by eq 23 predicts the existence of ordered and disordered phases. The disordered phase consist of a homogeneous mixture of ions and voids, while the ordered phase has cation and anions located on two sublattices (say A and B). *J* is the coupling constant describing the interaction between the ions. (b) Bethe lattice with coordination number *q* = 4, corresponding to the square lattice, has been used to calculate the charging properties analytically.^40,43,77^ (c) Charge on sublattices A and B, (d) capacitance, and (e) stored energy density as functions of the voltage. The symbols are the results of Monte Carlo simulations and the lines denote the Bethe-lattice solution. An ionic liquid with β*J* = 2 (red lines and triangles) is in the ordered state at low voltages and experiences a first-order transition to the disordered state at β*eu* ≈ 8 (red vertical line). An ionic liquid with β*J* = 0.2 (black lines and circles) is in the disordered state at all voltages. Adapted with permission from ref (43). Copyright 2021 American Chemical Society under CC-BY (https://creativecommons.org/licenses/by/2.0/).

A straightforward generalization of the single-file lattice model (section 4.1.1) is a 2D confinement. In this case, the Blume–Capel Hamiltonian reads
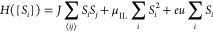
23where ⟨*ij*⟩ denotes nearest neighbor sites and *J* = ϕ_++_(*d*) with ϕ_++_(*r*) given by eq 9 (instead of eq 11). Note that eq 23 is identical to eq 18, except that the sum in eq 18 runs over the single index of a 1D lattice. In 2D, the BC model depends on the lattice type (square, triangular, etc.), and, unlike 1D, there is no known exact solution. However, an approximate analytical solution can be obtained using a Bethe-lattice approach. Within this approach, the partition function is evaluated on a Bethe lattice with the same coordination number *q* (i.e., the number of nearest neighbors) as the original lattice (Figure 6a,b). In particular, *q* = 3, 4, and 6 for the square, honeycomb, and triangular lattice, respectively. The partition function on the Bethe lattice can be computed analytically. A group of researchers led by Oshanin applied this approach to ionic liquids in nonpolarized slit pores^77^ and to nanoslit charging.^40,43^ They found that an ionic liquid can be in two states: ordered and disordered. The ordered state consists of two differently charged sublattices, while the disordered phase is a homogeneous mixture of ions and voids/solvent molecules (Figure 6a). Surprisingly, the Bethe-lattice approximation showed an excellent quantitative agreement with Monte Carlo simulations of the same model (symbols and lines in Figure 6c–e).^43^ Most recently, Groda et al.^78^ revealed that next-to-nearest and higher neighbor interactions in eq 23 can lead to the emergence of large-scale mesophases that have not been reported before.

#### 4.2.3 Phase Transitions

An exciting feature that distinguishes 2D confinements from single-file pores (1D confinements) is the possibility of phase transitions, which cannot occur in 1D.^79,79^ An example is shown in Figure 6c for the lattice model, eq 23. At low potential differences, an ionic liquid (with coupling constant β*J* = 2) is in the ordered state, characterized by positive and negative charges on two sublattices. As the voltage increases, there is a phase transition to a disordered state with equal ionic charges on both sublattices, corresponding to a homogeneous mixture of ions and voids/solvent (Figure 6c). The transition occurs when the voltage is sufficiently strong to break the ordered structure. Notably, the capacitance vanishes in the ordered state (low voltages) and exhibits a peak at the transition (Figure 6d). In contrast, for an ionic liquid with weaker coupling (β*J* = 0.2), the capacitance is nonzero at low voltages but vanishes as the voltage increases. Thus, ionic ordering at zero voltage shifts the region of “active charging”, i.e., the charging characterized by a nonzero capacitance, to higher voltages, which enhances energy storage considerably (Figure 6e). This effect may find practical applications.

**Figure 7:**
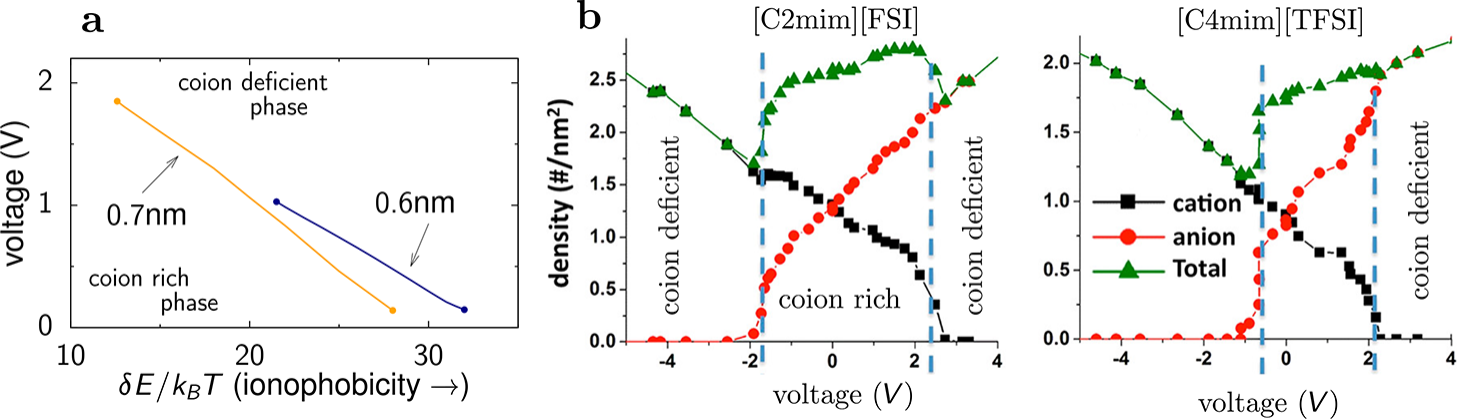
Phase transitions in slit confinement. (a) Phase diagram in the plane of voltage and pore ionophobicity (the energy of insertion of an ion into the pore) showing voltage-induced transitions between cation-rich and cation-deficient phases. The lines of first-order transitions end at critical points denoted by filled circles. Adapted with permission from ref (76). Copyright 2016 American Physical Society under CC-BY (https://creativecommons.org/licenses/by/3.0/). (b) Results of molecular dynamics simulations showing an abrupt drop in the co-ion density, indicating a first-order transition between cation-rich and cation-deficient phases (positive voltages) and between anion-rich and anion-deficient phases (negative voltages). The blue vertical lines denotes the locations of the transitions. Adapted with permission from ref (80). Copyright 2015 American Chemical Society.

A different type of transition has been reported in refs (23) and (76), for the continuum model, described by eq 20. The authors of ref (23) revealed that charging could proceed discontinuously via an abrupt expulsion of co-ions from a pore. They found a line of voltage-induced first-order phase transitions that ends at a critical point (second-order transition) as the slit width decreases. Lee et al.^76^ investigated how such transitions depend on the pore ionophilicity, i.e., an energy of transfer of an ion from the bulk into the nanopore. They found that, for a fixed slit width, a line of first-order transitions ends with two critical points at high and low ionophilicities (Figure 7a). The existence of first-order phase transitions between co-ion rich and co-ion deficient phases have been confirmed by atomistic molecular dynamics simulations of [C_2_MIM][FSI] and [C_4_MIM][TFSI] ionic liquids in slit nanopores^80^ (Figure 7b). Similar voltage-induced transitions between low- and high-density phases have been reported by Kiyohara et al.,^81−83^ who used their approximate Grand Canonical constant-potential Monte Carlo simulations.^84^ More recently, Mossa^85^ revealed phase transitions between liquid-like (disordered) and crystal-like (ordered) phases in uncharged slit pores using molecular dynamics simulations. We note, however, that this author considered nonconducting pores (i.e., constant charge simulations, see sections 6.1.6 and 6.1.7).

### 4.3 Large-Voltage Asymptotic Behavior

Using the exact solution of the 1D off-lattice model (section 4.1.2), Verkholyak et al.^44^ have found that, perhaps surprisingly, the leading-order terms of the large-voltage asymptotic expansion of the differential capacitance do not depend on interionic interactions. This observation motivated a simple phenomenological model to describe the asymptotic behaviors at large voltages. Considering that only counterions are present in a pore, ref (44) assumed for the free energy density
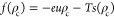
24where ρ_c_ is the counterion density and *s*(ρ_c_) the entropy density. Note that eq 24 does not contain electrostatic or any other interactions. It also does not include an ideal gas entropy, −*k*_B_ρ_c_ ln(ρ_c_/ρ_max_), which is finite at nonzero densities and does not contribute to the asymptotic behavior. Using the exact expression for the entropy of a 1D hard-rod fluid,^66^
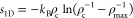
25where ρ_max_ = (2*a*)^−1^ is the maximum achievable density, gives for the capacitance (per unit length) in the leading order in *u* (ref (44))
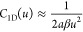
26For spherical ions, asymptotic behavior in slit pores is complicated by transitions to hexatic and solid phases.^86−89^ However, it is not clear whether such phases exist for ILs. Verkholyak et al.^44^ assumed that at high voltages, the counterions are in a fluid state, which is reasonable for packing fractions η = πρ_c_*a*^2^ ≲ 0.7. They then used in eq 24 the scaled-particle results for the entropy of a 2D hard-disk fluid,^90−92^
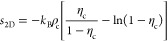
27which gives for the capacitance per surface area in the leading order in *u*
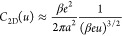
28Thus, for slit pores, the decay of capacitance for increasing voltage is slower than for single-file pores. We recall that for planar electrodes, the capacitance *C* ∼ *u*^–1/2^,^69^ showing the slowest decay among all three geometries, as one may expect.

Plugging eqs 26 and 28 in eq 4, one obtains that in the limit of large voltages, *u* → *∞*, the stored energy (per unit length) increases logarithmically with voltage,
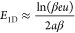
29for single-file pores, while for slit pores, the energy (per surface area) grows as a square-root of voltage,
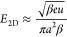
30This result means that at large voltages, slit pores are more efficient in storing energy than single-file pores. It contrasts with low voltages, where the screened electrostatic interactions, rather than entropic interactions, determine the system behavior. In the low-voltage regime, the screening is stronger in single-file pores (section 3.1), implying that such pores store energy more efficiently.^51^

### 4.4 Quantum Capacitance

Quantum capacitance emerges in nonideally metallic electrodes due to the finite density of states of electrons such as graphite and carbon nanotubes (CNT). Gerischer^93^ pointed out that for graphite electrodes, the leading contribution to the measured capacitance (particularly at low applied voltages) is due to a “space-charge” (or quantum^94^) capacitance of graphite, rather than due to an electrical double layer (EDL) capacitance. The total measured capacitance is^93^
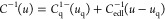
31where *C*_q_ and *C*_edl_ are the quantum and EDL capacitances and *u*_q_ is the potential drop across the electrode (which was graphite in ref (93)). Note that *C* = *C*_edl_ in the rest of the review, where we have assumed *C*_q_ = *∞* as for an ideal metal electrode. Equation 31 suggests that a finite quantum capacitance is detrimental to capacitive energy storage, as it tends to decrease the total capacitance.

**Figure 8:**
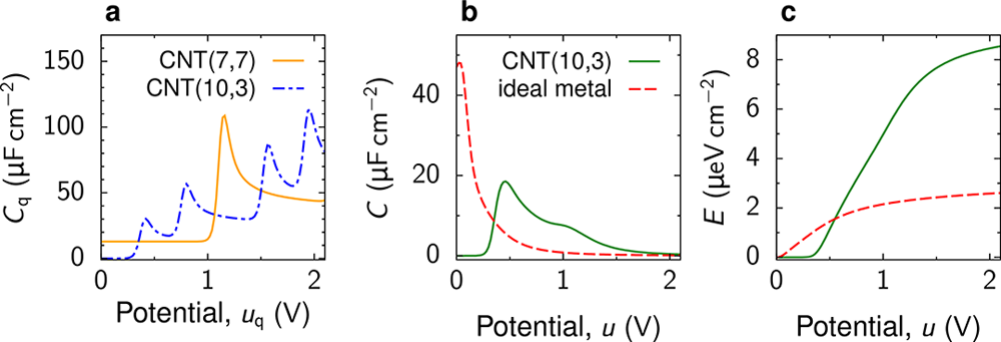
Effect of quantum capacitance. (a) Quantum capacitance of a metallic (chiral indices 7,7) and semiconducting (10,3) carbon nanotubes (CNTs) obtained using the Mintmire–White formula.^102^ (b) Total capacitance and (c) stored energy density of the (10,3) CNT and of a nanotube of the same size as the CNT but made of the ideal metal (i.e., assuming *C*_q_ = *∞*). Adapted with permission from ref (72). Copyright 2022 American Chemical Society under CC-BY (https://creativecommons.org/licenses/by/2.0/).

Quantum capacitance appears in many carbon-based nanoporous electrodes, particularly in an emerging class of novel low-dimensional electrode materials. Nevertheless, the effect of quantum capacitance still needs to be better understood. Most studies have focused on flat electrodes^95−97^ and ionic liquids outside carbon nanotubes (CNT),^98−101^ repeatedly showing that *C*_q_ reduces the total capacitance. To our knowledge, only a recent work^72^ studied the role of quantum capacitance for confined ionic liquids. In particular, Verkholyak et al.^72^ considered CNTs and used the exact analytical solution to compute *C*_edl_ (section 4.1.2) and the Mintmire–White formula^102^ to calculate *C*_q_. Two examples of *C*_q_ for metallic and semiconducting CNTs are shown in Figure 8a. The quantum capacitance is low and nearly constant for a metallic CNT, exhibiting a peak at the van Hove singularity, a plateau before the second van Hove singularity, and so on. The semiconducting CNT shows a similar behavior, except the capacitance vanishes around zero voltage (in the band gap), and the peaks are at different positions.

The analytical solutions allowed Verkholyak et al.^72^ to analyze the capacitance in a wide range of voltages systematically. They revealed that, surprisingly, a low quantum capacitance could enhance the total capacitance at intermediate to high voltages, where the EDL capacitance vanishes due to pore saturation (i.e., when *C*_edl_ → 0). A comparison of the semiconducting CNT and a nanotube of the same size but made of the ideal metal (*C*_q_ = *∞*) is shown in Figure 8b. Note that the total capacitance of the CNT vanishes at low voltages (within the band gap), and hence the stored energy calculated with eq 4 vanishes too. However, the CNT delivers even a few times higher stored energy densities at moderate and high voltages. For metallic CNTs, the behavior is similar, but the low-voltage capacitance and stored energy are nonzero.^72^

This surprising and encouraging result suggests that reducing the quantum capacitance can enhance energy storage in the case of nanopore saturation. However, more work must be done to better understand and benefit from such effects.

### 4.5 Dynamics of Charging

**Figure 9:**
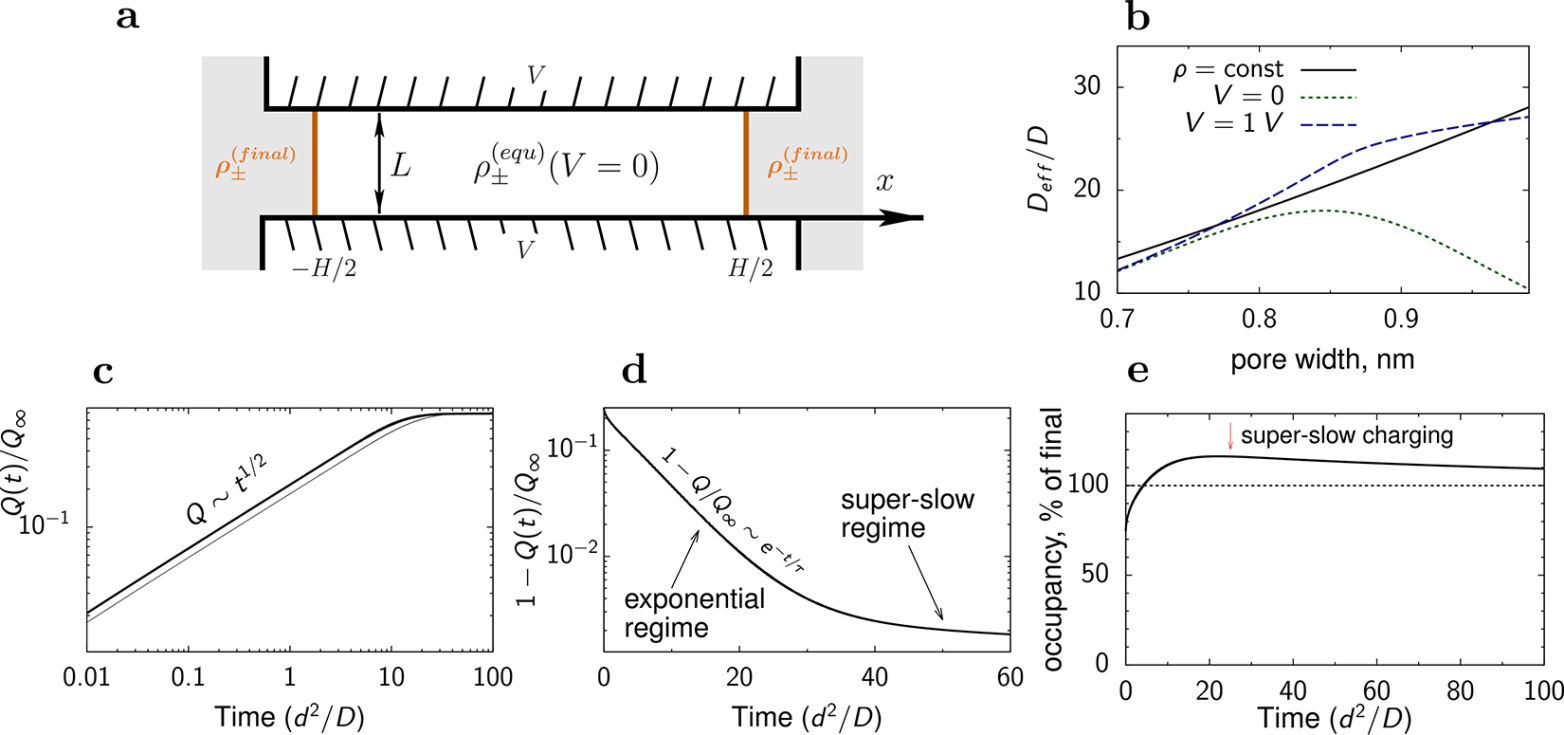
Charging narrow slit nanopores. (a) Model of an open, slit-shaped metallic nanopore. Slit width *L* is comparable to the ion diameter, so that an ionic liquid is approximated as a 2D fluid (not shown). The in-pore ion densities at *t* = 0 are equilibrium densities at zero voltage. The ion densities at the pore entrances are the equilibrium densities at potential *V* applied to the slit walls with respect to bulk electrolyte (i.e., outside of the pore); these densities are kept constant at all times during charging. (b) Effective diffusion coefficient, eq 35, as a function of pore width for constant density and for two values of the applied potential *V*; the dependence of *D*_eff_ on *V* is through the ρ(*V*) dependence. (c,d) Accumulated charge as a function of time. The charge grows as a square-root of time at short times (c). At late times, there are two exponential regimes (d). (e) Pore occupancy with respect to the equilibrium occupancy at voltage *V*. Time is expressed in units of *d*^2^/*D*, where *D* is the diffusion coefficient and *d* the ion diameter. (b) Adapted with permission from ref (112). Copyright 2013 American Chemical Society. (a,c–e) Adapted with permission from ref (113). Copyright 2014 Springer Nature.

Despite its importance, studies of charging dynamics in ultranarrow pores are still scarce, particularly with analytical models. The main focus so far has been on electrodes with pores substantially wider than the ion diameter, often wider than the thickness of an electrical double layer^103−108^ or with overlapping double layers but dilute electrolytes.^109,110^ To study the charging of single-file and ultranarrow slit pores, refs (111) and (112) assumed that ion dynamics outside the pore is sufficiently fast to maintain the near-equilibrium ionic densities at the pore entrance at all times during charging (Figure 9a). Molecular dynamics simulations showed that this assumption is reasonable.^113^

The evolution of ion densities can be determined from the continuity equation
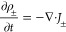
32where *J*_±_ are the ionic currents. The dynamical models rely on deriving or constructing suitable expressions for *J*_±_. The main questions to address are how quickly the charge propagates inside such pores and how the charging dynamics depends on the pore properties, particularly on pore’s width and ionophilicity.

#### 4.5.1 Narrow Slit Pores

In the spirit of a dynamical density functional theory (section 5.2.8), ref (112) assumed that *J*_±_ are proportional to the gradients of the chemical potentials, which are variational derivatives of the free energy with respect to ionic densities, i.e.,
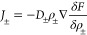
33where *D*_±_ are transport diffusion coefficients of cations and anions, further assumed equal and density-independent, for simplicity. To compute currents, ref (112) used free energy (eq 20), extended to a free energy functional accounting for spatially varying densities. This procedure leads to an equation for ionic densities that needs to be solved numerically. However, by assuming that the total ion density does not change during charging, i.e., that the charging mechanism is exclusively ion swapping (section 2.3), this equation simplifies to a diffusion equation for the charge density *c* = *e*(ρ_+_ – ρ_–_) (ref (112))
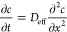
34where
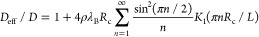
35is the effective diffusion coefficient describing the collective modes of charging due to the interionic interactions in the superionic state. Here *K*_1_ is the first-order modified Bessel function of the second kind and *R*_c_ is the disk cut-out radius (see section 4.2.1). Figure 9b shows *D*_eff_ as a function of the slit width *L* and demonstrates that it increases with increasing *L* in most cases considered. This implies that charging is faster for wider pores, opposite to the capacitance, which decreases with increasing the pore width (Figure 5b) (the capacitance also shows small oscillations for pores larger than 1 nm, cf. section 5.2.1 and Figure 26).

Solving eq 34 for a pore of length  shows that at short times, the charge grows as a square-root of time^112,113^
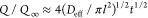
36and at late times, the charge saturates exponentially
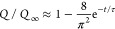
37where  is the decay constant and *Q*_*∞*_ is the equilibrium charge (at *t* = *∞*). Numerical solution of the full eq (eq 32) revealed^112^ that, in addition to these two regimes, there is also a “superslow” regime when the charge saturates exponentially but with a larger decay constant (Figure 9c,d). The superslow regime is related to pore overfilling, occurring at an intermediate stage of charging (Figure 9e). These three charging regimes have been confirmed with time-dependent density functional theory (section 5.2.8 and Figure 17). Molecular dynamics simulations, in addition, predicted a linear regime at early times (section 6.4.2 and Figure 30), which is not captured by the analytical theory.

Numerical analyses also showed that ionophobic pores, which are empty at no polarization, charge in a front-like manner, with the charge growing as a square-root of time, similarly to ionophilic pores.^112^ The calculations showed that ionophobic pores charged faster than ionophilic ones, but the effect within this approach was not as drastic as later unveiled by molecular dynamics simulations of a similar model^113^ (section 6.4.4 and Figure 32).

#### 4.5.2 Quasi Single-File Pores

Charging single-file pores is tricky. An empty (ionophobic) pore can charge by adsorbing counterions, but if a pore is filled with ions at zero voltage, virtually all ions need to leave the pore before charging can even commence. Of course, this is not a very satisfactory scenario. However, real ions are often nonspherical, while the pores are not ideally cylindrical, and their radii may slightly vary along the pore. All this may allow the ions to pass each other during charging even in otherwise single-file pores (we shall call such pores quasi single-file). This process of ion swapping has been taken into account by Lee et al.,^111^ who introduced a microscopic master equation and performed its coarse-graining on the mean-field level. They arrived at the following equation for ionic currents ( and )
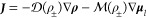
38where  and  are the diffusion and mobility matrices, which depend on the diffusion and swapping rates and ion densities, and  is the long-range part of the excess chemical potential determined by the screened electrostatic interactions. The equations for ,  and **μ**_*l*_ are lengthy and not reproduced here (see ref (111)). Similarly to slit pores, the charging dynamics is dictated by the continuity equation, eq 32. Numerical solution of this equation revealed^111^ that ionophobic pores charge diffusively, i.e., the accumulated charge grows as a square root of time at short times and saturates exponentially at late times. For moderately filled pores, there is a front of co-ions leaving a pore, while for ionophilic pores, there is overfilling (ion crowding) at the pore entrance which hinders fast charging, similarly to slit pores. The calculations also showed that cyclic charging proceeds via overfilling even for ionophobic pores, which occurs when the voltage is reversed. However, overfilling could be avoided by using the charging cycle *u* → 0 → ± *u* instead of *u* → – *u* → *u*.

#### 4.5.3 Equivalent Circuit Models

**Figure 10:**
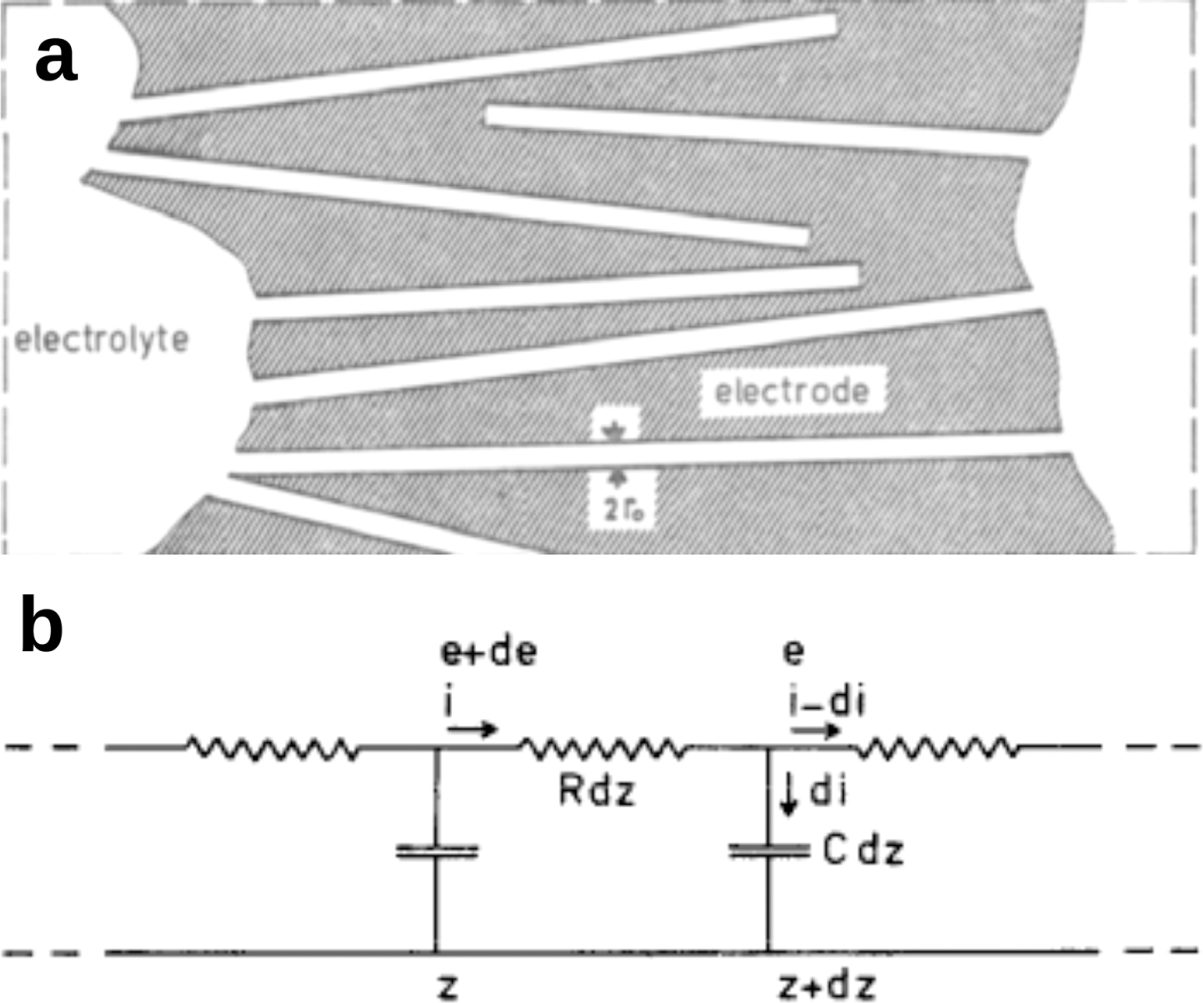
Equivalent circuit model. (a) Schematic of a cross-sectional view of a porous electrode. The electrode surface consists of circular cylinders of radius *r*_0_ much larger than the ion size and double layer thickness. (b) A small section of an equivalent circuit of a pore with uniformly distributed resistance *R* and double layer capacitance *C* (per unit length); *z* is the distance from the pore entrance, and *e* and *i* denote the potential and current, respectively. Reproduced with permission from ref (103). Copyright 1963 Elsevier.

Equivalent circuit models (ECM) rely on using electrical components to describe electrochemical processes and are particularly popular in studies of the dynamics of charging porous electrodes (see ref (114) for review). de Levie^103,115^ constructed a so-called transmission line model (TLM) with the pore’s capacitances and ionic resistances distributed uniformly over infinitesimally small capacitors and resistors arranged along the pore (Figure 10). Assuming voltage and position independent capacitance *C* and resistance *R* (both per unit length), de Levie obtained a diffusion-like equation for the electrostatic potential along the pore^103^
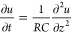
39This model has variations,^114^ such as thin nanoporous electrodes consisting of just two equivalent capacitors and resistors (Figure 31a).^116,117^ The TLM has also been extended to arbitrary-length pores^108,118^ and electrosorption with redox materials.^119^

The TLM can be derived from the Nernst–Planck equation for ion dynamics combined with the Gouy–Chapman model for an electrolyte.^106,120,121^ For instance, Biesheuvel and Bazant^106^ expanded these equations in a small parameter λ = λ_D_/*L*, where λ_D_ is the Debye length and *L* is the pore width, and obtained the TLM equation for small applied potentials (below thermal voltage) in the first order in λ, i.e., for the double-layer thickness much smaller than the pore width. This derivation shows that an ECM amounts to splitting the charging into capacitive and resistive processes, which appears natural for pores wider than the Debye length. However, it might no longer be valid for narrow pores,^122^ where the capacitive and resistive processes are likely strongly coupled.

Nevertheless, ECM, TLM, and their modifications have frequently been applied to nanoporous electrodes.^116,117,123,124^ As noted in refs (113) and (125), the partial success of the TLM is probably due to its mathematical similarity with the diffusion-like mean-field equation for the charge density in subnanometer pores (eq 34). The physical meaning of the time scales appearing in the two equations differs, however. Instead of the RC time, which depends on the bulk resistivity and zero-voltage capacitance of a flat electrode (eq 39), there is an effective diffusion coefficient due to the reduced electrostatic interactions in narrow confinement (section 4.5.1).

While the TLM could describe the square-root and exponential diffusive regimes, similarly to eq 34, it failed to predict the superslow regime (Figure 30). However, a modification of the TLM showed a linear regime for a large bulk resistance.^121,126^ In recent work, Mo et al. used the TLM model for an electrode consisting of two slit pores. These authors showed that, unlike capacitance, the “resistivities”, obtained by fitting the simulation results to the TLM, do not uniquely describe the pores and depend on how the charging proceeds in other pores, casting doubts on the immediate applicability of the TLM to nanopores. We also note that most ECMs assume that the capacitance and resistivities are voltage-independent, while numerous computational studies (Figures 3, 4, 6, 13, 15, and 26) and some experiments^70^ report voltage-dependent capacitance.

## 5 Classical Density Functional Theory

### 5.1 Density Functional Theory for Classical Fluids

Classical density functional theory (DFT) originates from the quantum DFT, which is rooted in the celebrated Thomas–Fermi model^127,128^ for calculating the electronic structures of many-body quantum systems. The modern quantum DFT was established in the mid 1960s by Hohenberg and Kohn,^129^ who showed that the electron density uniquely determines the ground-state properties of such systems. They defined an energy functional and proved that it is minimized by the system’s electron density, offering a variational approach to calculating it. Kohn and Sham^130^ developed approximate methods to treat inhomogeneous systems and Mermin^131^ extended the theory to nonzero temperatures. In 1998, Walter Kohn received a Nobel Prize in Chemistry “for his development of the density-functional theory”.^132^

Ideas to apply variational density functionals to classical fluids appeared around the same time. In 1964, van Kampen^133^ introduced a functional of space-dependent particle density to investigate condensation of a classical gas with long-ranged attractive interactions. However, it was not until mid 1970s that classical density functionals received more attention. In 1976, Ebner et al.^134,135^ formulated a density functional based on the Mermin^131^ work and applied it to calculate surface tension and density profiles for a Lennard-Jones fluid at a hard wall. Three years later, Evans^136^ derived a density functional by applying Mermin’s work^131^ to classical fluids and used it to study liquid–gas interfaces. Nordholm and Haymet^137^ formulated their version of (van der Waals) density functional and Johnson and Nordholm^138^ applied it to the adsorption of inert gases at a flat wall. Haymet and Oxtoby^139^ developed a molecular density functional and used it to study solid–liquid interfaces.^140^ These works collectively founded the classical DFT that is now widely used to study various systems, including confined ionic liquids.

In the classical DFT, the key quantity is the grand potential functional
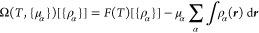
40where ρ_α_(***r***) is the three-dimensional density of species α and
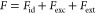
41is the free energy functional. Here
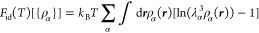
42is the free energy of the ideal gas mixture (λ_α_ is the de Broglie wavelength of particles of species α),
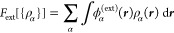
43is the free energy due to external potential φ_α_^(ext)^(***r***), and (see, e.g., ref (136))
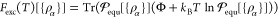
44is the “excess” free energy. In eq 44, Φ describes intermolecular interactions,  is the equilibrium probability density, which depends functionally on φ_α_^(ext)^ and hence on the particle densities, and
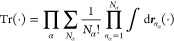
45is the configurational part of the usual “classical” trace (compare eq 14).

Evaluated at the equilibrium densities ρ_α_^equ^(***r***) corresponding to given temperature *T*, chemical potentials μ_α_ and external potentials φ_α_^(ext)^(***r***), eq 40 gives the equilibrium Grand Canonical free energy Ω_equ_. In the spirit of the quantum DFT,^129−131^ it can be shown (see, e.g., ref (136)) that Ω_equ_ corresponds to a minimum of Ω with respect to ρ_α_, which means that the variational derivative of Ω with respect to densities vanishes, i.e.,
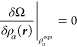
46The solution of eq 46, therefore, gives the equilibrium density
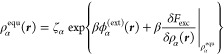
47where  is the fugacity of species α.

Formally, eqs 40 and 47 are exact. However, the equilibrium distribution  needed to calculate *F*_exc_ is generally not known and approximations are necessary. Below we discuss a few approximations relevant for (and frequently applied to) confined ionic liquids.

#### 5.1.1 Approximations for the Excess Free Energy

For hard sphere systems, a reliable approximation is the fundamental measure theory (FMT) introduced by Rosenfeld^141^ in 1989. He started from the low density expansion
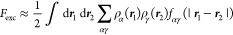
48where *f*_αγ_(*r*) = 1–exp{−βψ_αγ_} = 1 for *r* < *d*_αγ_ = (*d*_α_ + *d*_γ_)/2 and zero otherwise, *d*_α_ and *d*_γ_ are the ion diameters and ψ_αγ_ is the hard-core interaction potential between species α and γ. Rosenfeld noticed^141^ that these Mayer functions could be decomposed into the convolution of scalar and vectorial fundamental geometrical measures *w*_α_^*i*^ (*i* = 0, ..., 3) and ***w***_α_^*i*^ (*i* = 1, 2), associated with volume, surface area, curvature and Euler characteristics of particles (see ref (142) for definitions). To account for high densities, Rosenfeld used weighted densities approach and derived the excess free energy functional for a hard sphere system^141,142^
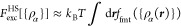
49where
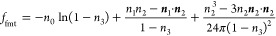
50and
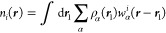
51and similarly for vectorial quantities ***n***_1_ and ***n***_2_ by replacing *n*_*i*_ and *w*_α_^*i*^ with ***n***_*i*_ and ***w***_α_^*i*^ in eq 51. The Rosenfeld’s FMT turned out successful in describing the structure and thermodynamics of hard-sphere mixtures but failed at predicting hard-sphere crystals. Several versions of the FMT have been proposed to correct this deficiency and improve the accuracy.^143−149^ The most recent and probably the most popular one is the White Bear mark II version proposed by Hansen-Goos and Roth.^149^ For the details of the FMT theory, see a review by Roth.^142^

For soft potentials, a useful approximation can be obtained from a Taylor expansion of *F*_exc_ around homogeneous bulk densities ρ_α_^bulk^. Keeping only the second-order terms gives the Ramakrishnan and Yussouff^150^ approximation (note the differences with eq 48)
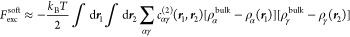
52where *c*_αγ_^(2)^ is the direct correlation function for αγ species evaluated at the bulk densities. The direct correlation functions are not known in general and further approximations are necessary. Frequently, though not exclusively, one uses the existing (bulk) solutions for *c*_αγ_^(2)^ obtained within the random-phase or mean-spherical approximations (RPA and MSA, respectively).

For systems where both hard-core and long-range interactions are essential, as for ILs, one often combines the FMT for hard-core with the MSA or RPA (or mean-field) approximations for soft interactions, such as electrostatic and/or Lennard-Jones, i.e.,
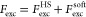
53taking the direct correlation functions of the full system. We note that eq 53 can be derived using a perturbation expansion with respect to densities.^151^

Despite several approximations, some of which might be questionable, classical DFT is a convenient and computationally inexpensive method to study ionic systems taking into account ion sizes and (some) ionic correlations. For a planar electrode, the DFT results obtained within these assumptions compared favorably with molecular dynamics simulations.^152−157^ A comparison for ILs confined in single-file pores was also favorable,^158^ but deviations were reported for slit-shaped pores.^159^

#### 5.1.2 Time-Dependent Density Functional

One can construct DFT-based equations for densities’ time evolution as follows.^160^ For given density profiles ρ_α_(***r***, *t*), the free energy (eq 41) defines the thermodynamic force acting on particles α, that is, ***f***_α_ = −∇δ*F*/δρ_α_. Then the total current of particles α is ***j***_α_ = β*D*_α_ρ_α_***f***_α_, where *D*_α_ is a transport diffusion coefficient (not to be confused with self-diffusion coefficient). The local conservation law dictates that the total change in the particle density at time *t* is determined by the gradient of the current, i.e., ∂ρ_α_/∂*t* = −∇·***j***_α_, giving
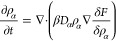
54We note that *D*_α_ in general depends on densities, although it is often assumed density-independent. Using the ideal gas free energy for *F* (eq 42) gives the diffusion equation
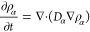
55as one may expect.

There have been several attempts to derive a dynamical density functional from microscopic equations.^161−166^ Starting from the Langevin stochastic equation, Dean^162^ derived an equation similar in form to eq 54 but with a different (mean-field like) expression for *F* and containing stochastic noise. Marconi and Tarazona^164,167^ used the same approach but averaged over the noise and arrived at the deterministic equation (eq 54), making the first direct connection with the equilibrium DFT. Archer and Evans^165^ derived the dynamical DFT starting from Smoluchowski equation. Español and Löwen^166^ derived a generic dynamical density functional using the projection operator technique and showed that it reduces to the standard dynamical DFT (eq 54) only for a dilute suspension of colloidal particles. This conclusion is consistent with the derivations based on the Langevin and Smoluchowski equations.

Unlike static DFT, which is formally exact, the dynamical DFT relies on various assumptions. The essential assumption is that the correlation functions of a nonequilibrium system are equal to the correlation functions of the system in equilibrium corresponding to the same particle density.^164^ This assumption implies local equilibrium and that the densities change over time infinitely slowly (quasistatically), which is not always the case.^168^ For the details of the time-dependent DFT and its applications, see a recent comprehensive review by te Vrugt et al.^34^

### 5.2 Application to Ionic Liquids under Narrow Conducting Confinement

Being computationally inexpensive (compared to simulations) and accounting for ion sizes and some correlations, static and dynamic DFTs have become a popular tool to study various phenomena in confined ionic liquids, from anomalous capacitance increase^169^ and solvent effects^170−172^ to charging dynamics^173−175^ and temperature effects.^159,176,177^ In this section, we briefly describe the main phenomena studied with DFT. We refer the interested readers to a review by Härtel,^178^ which covers the application of DFT to confined ILs before 2017, with a particular focus on technicalities. There is also a more recent review by Lian and Liu,^179^ who mainly focused on their own contribution.

#### 5.2.1 Anomalous Capacitance and Capacitance Oscillations

**Figure 11:**
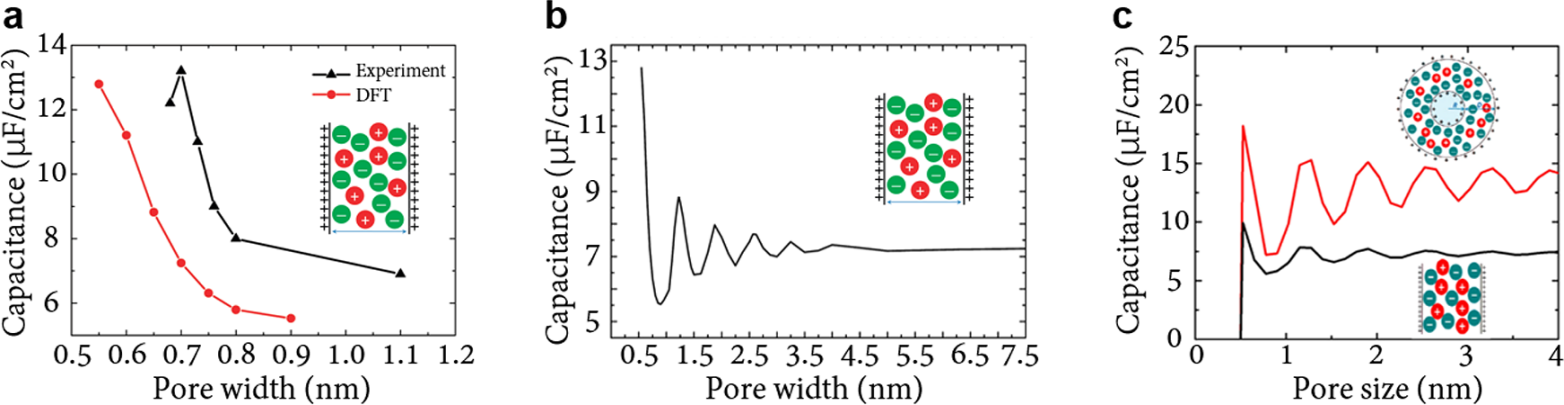
Anomalous capacitance increase and oscillating capacitance in nanopores from classical DFT. (a) Capacitance as a function of slit width: classical DFT vs experiments of ref (20). (b) Capacitance shows oscillations as a function of slit width. Reproduced with permission from ref (169). Copyright 2011 American Chemical Society. (c) Convex pore shapes increase capacitance compared to slit pores. Reproduced with permission from ref (180). Copyright 2016 American Chemical Society. In DFT calculations, a voltage of 1.5 V was applied with respect to the bulk electrolyte.

Perhaps the first application of classical DFT to confined ILs has been to study anomalous increase of capacitance in ultranarrow pores reported in refs (18−20). Jiang et al.^169^ considered an infinitely extended slit pore and a restrictive primitive model (RPM, that is, charged hard spheres) for ions. These authors calculated the integral capacitance as a function of the slit width at potential difference 1.5 V with respect to the bulk (Figure 11a, compare Figure 5). They found a fairly good qualitative agreement with experiments, although the calculated values of the capacitance were generally lower than experimental ones. Possible reasons are the integral vs differential capacitance, simplified IL/pore models, distribution of pore sizes, etc. Given these factors, however, the agreement is encouraging.

Jiang et al. also predicted oscillations of the integral capacitance as a function of pore size for pores wider than the ion diameter^169^ (Figure 11b). They related this behavior to the interference of the overlapping electrical double layers originating from two pore walls. A similar oscillatory behavior has been found by MD simulations^181,182^ (see section 6.3). Lian et al.^180^ showed that the oscillatory behavior persists also for curved pores, which generally provide higher capacitances compared to slit pores (Figure 11c).

#### 5.2.2 Ionophobic Pores

Motivated by earlier analytical and simulation work,^60,71,76^ Lian et al.^183^ studied how the ionophobicity of pores affects capacitance and energy storage for nonaqueous electrolytes and ILs. These authors considered slit pores, an RPM for ions, and effective dielectric medium for solvent. They found that the shape of the capacitance as a function of voltage changes from bell-like to camel-like upon increasing the ionophobicity. This behavior has some similarities with single-file pores, which show camel-shaped capacitance for strongly ionophilic and ionophobic pores and bell-shaped capacitance for moderately filled pores^44^ (Figure 3b,c). However, Lian et al.^183^ did not consider strongly ionophilic pores. Nevertheless, these authors confirmed that ionophobic pores provide higher stored energy densities at large voltages, in accord with ref (71). Interestingly, the stored energy density was practically independent of the bulk IL density (in the concentration range from 1 to 3.5 M).

#### 5.2.3 Effect of Solvent

**Figure 12:**
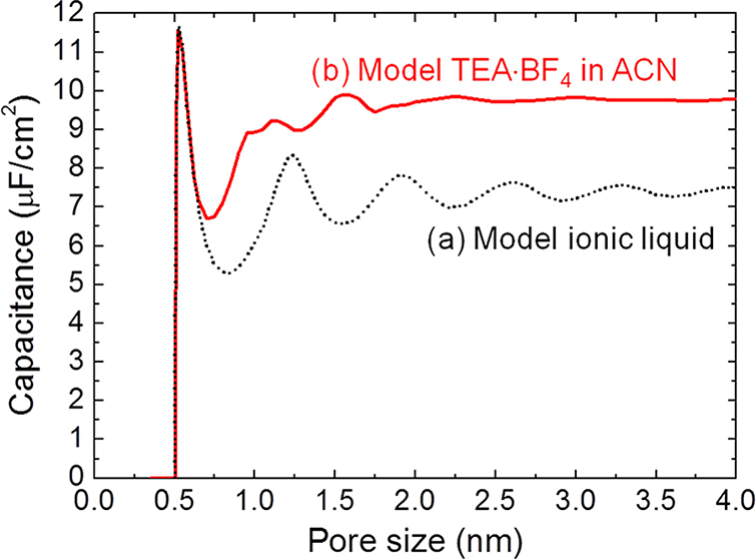
Effect of solvent on capacitance from classical DFT. Integral capacitance of an organic electrolyte ([TEA][BF_4_] in acetonitrile) flattens compared to the capacitance of neat ionic liquids. Reproduced with permission from ref (184). Copyright 2013 American Chemical Society.

The effect of solvent has been extensively studied with classical DFT. Jiang et al.^170^ considered two connected charged hard spheres as a model for polar solvent. These authors found that polar solvents significantly reduce the anomalous increase and oscillatory behavior of capacitance with decreasing pore size (Figure 12). They showed that the “flattening” of the capacitance-pore size curve is likely due to the orientational structuring of the solvent. Combined with pore-size distributions of carbon electrodes, this result may explain the controversies in experiments demonstrating a “regular pattern”^21^ instead of the anomalous increase^18−20^ of capacitance. In follow-up work, Jiang and Wu^185^ showed that polar solvents could be used to maximize capacitance. For a 4 nm wide pore, they found a peak in capacitance for the solvent’s dipole moment ≈ 4 Debye, slightly larger than acetonitrile. Liu and Wu^186^ showed that even a minor concentration (≈10^–4^ molar fraction) of a small polar solvent could boost the integral capacitance two-fold.

Liu et al.^171^ studied how nonpolar solvents (which they called “impurities” considering only low bulk molar fractions = 10^–4^) affect the electrical double layer structure and integral capacitance. They found that strong solvent adsorption at the pore walls reduced the oscillatory behavior of the capacitance and systematically lower its value (at applied potential 1.5 V with respect to the bulk). This effect emerges because the solvent dominates the interfacial layers at the pore walls. Notably, the binding energies at which such flattening occurred were above 20 *k*_B_*T*. In contrast, the formation of strong solvation layers around the ions could enhance the oscillatory behavior and increase the capacitance because it facilitates ion ordering inside the pores due to entropic effects. Again, the binding energies considered by Liu et al.^171^ were relatively high and amounted to 40 *k*_B_*T*. Lian et al.^172^ studied a higher solvent concentration (0.01 molar fraction) and found that the strong affinity of the pore walls to solvent could enhance energy storage at high voltages. This is because the solvent could play the role of an “ionophobic agent”,^62^ shifting the charging to higher voltages and thus increasing the energy density.^71^

#### 5.2.4 Effect of Shape

Dyatkin et al.^188^ studied the effect of cation chain length using [C_2*n*+2_MIM][[TFSI] ionic liquid (*n* = 2, 4, 6). Their DFT calculations showed that, similar to electrochemical experiments, the capacitance decreased with increasing *n*. However, in a follow-up work, Gallegos et al.^189^ demonstrated that the dependence on the chain length is more subtle. These authors found that the integral capacitance changes nonmonotonically with *n* and could increase or decrease depending on the pore size. They related this behavior to the structuring of the cation chains inside the pores.^189^

**Figure 13:**
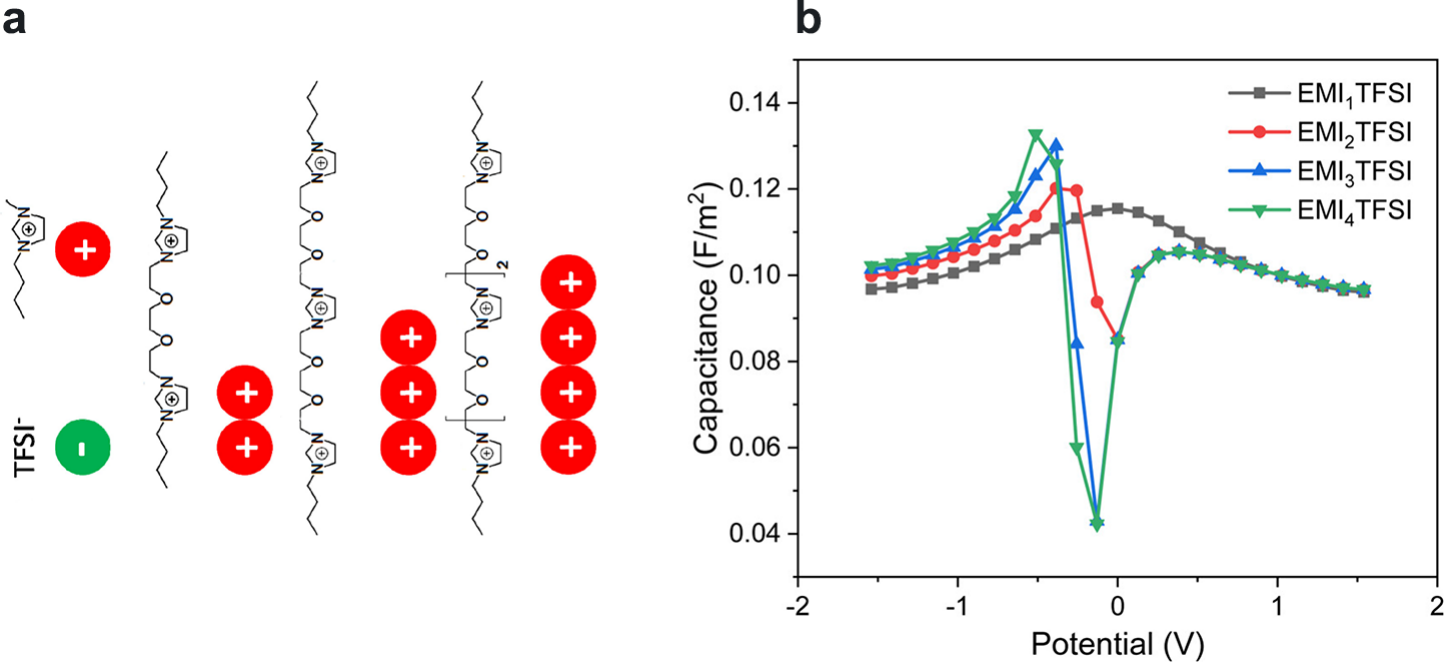
Effect of ion shape and valency on capacitance from classical DFT. (a) [TFSI]^–^ and “oligomerized” [EMIM]^+^ ionic liquids. (b) Differential capacitance of [TFSI]^–^ and oligomerized [EMIM]^+^ as a function of applied potential difference. Reproduced with permission from ref (187). Copyright 2018 American Chemical Society.

Motivated by the work of Matsumoto et al.,^190^ who studied oligomeric ionic liquids for planar electrodes in electrical double layer transistors, Lian et al.^187^ investigated model oligomeric cations in slit pores (Figure 13). These authors found that oligomerizing cations decreased the in-pore ion density, thus providing a means to create effectively ionophobic pores. As the result, they observed a transformation of the differential capacitance from bell-shaped (*n* = 1) to asymmetric camel-shaped for *n* > 2 (Figure 13, compare Figure 3c). However, this transformation provided only a small increase of the stored energy density at negative potentials.^187^

#### 5.2.5 Ionic Liquid Mixtures

Wang et al.^191^ considered a mixture of [EMIM][BF_4_] and [TMA][BF_4_] and showed with electrochemical measurements that it could simultaneously enhance the energy and power density of supercapacitors. Classical DFT calculations backed up these results concerning energy storage.^191^ The authors of ref (191) modeled [BF_4_]^–^ and [TMA]^+^ as charged spheres and [EMIM]^+^ as a dumbbell consisting of a charged sphere, mimicking the imidazolium ring, and a neutral sphere, imitating the ethyl group. They used square-well potentials to mimic van der Waals interactions between the ions. In line with experiments, they found that the capacitance increased with increasing the bulk concentration of [TMA]^+^ for mesopores, but it decreased for micropores. Their calculations suggested that this result is connected to the different van der Waals interactions between the ions rather than entropic effects.

**Figure 14:**
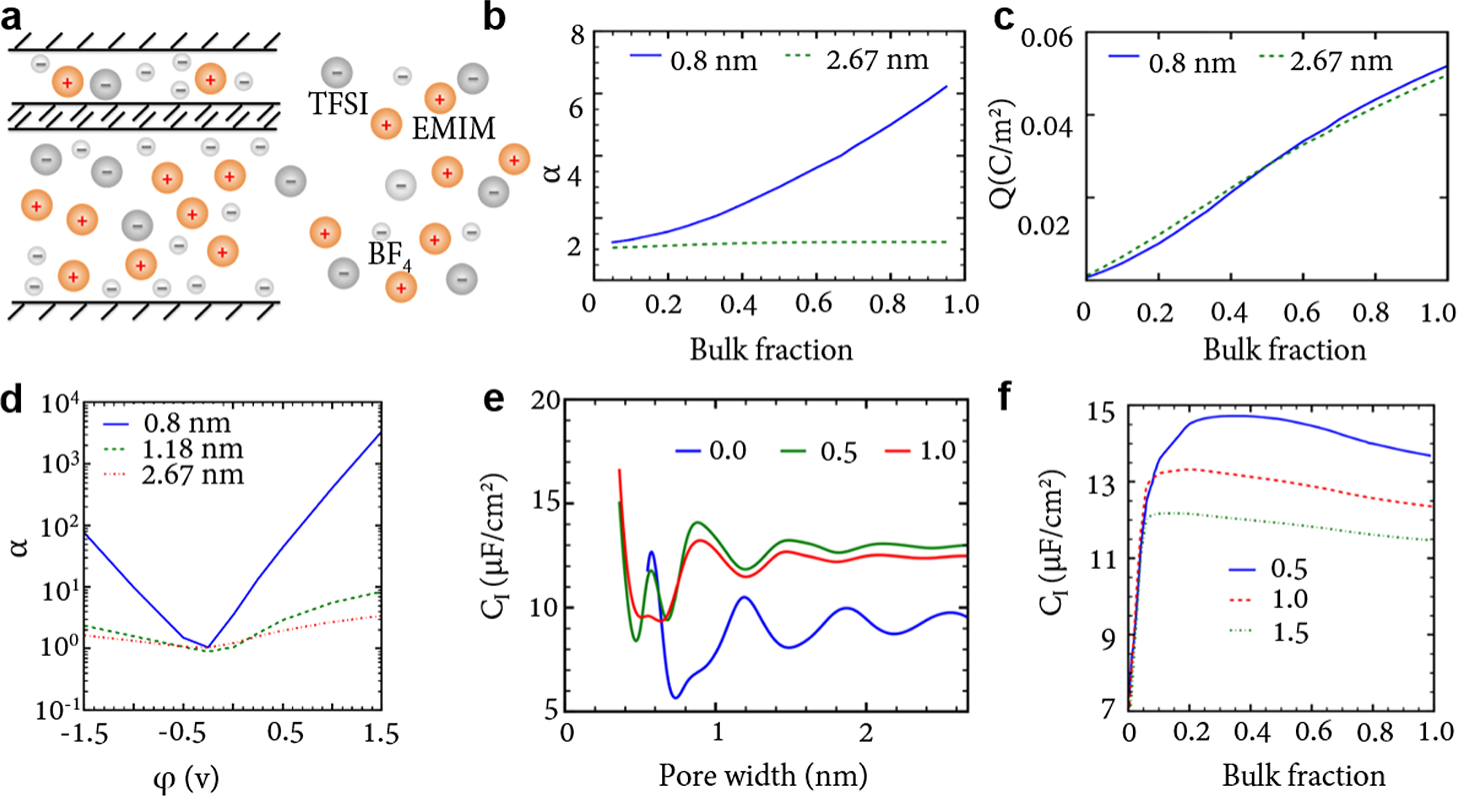
Effect of IL mixtures from classical DFT. (a) Schematics of micro- and mesopores and a mixture of ionic liquids. (b) Ion selectivity and (c) surface charge as a function of the bulk fraction *x* of [BF_4_]^–^ in a mixture of [EMIM]^+^, [TFSI]^–^, and [BF_4_]^–^ for micro- and mesopores. Reproduced with permission from ref (192). Copyright 2017 American Physical Society. (d) Ion selectivity as a function of applied potential difference with respect to the bulk electrolyte for a few pore widths and the same IL mixture as in the other panels. Reproduced with permission from ref (193). Copyright 2017 AIP Publishing. (e) Integral capacitance as a function of the pore width *H* in a mixture of [EMIM]^+^, [TFSI]^–^, and [BF_4_]^–^ for a few molar fractions of [BF_4_]^–^. (f) Integral capacitance as a function of the bulk fraction *x* of [BF_4_]^–^ in a mixture of [EMIM]^+^, [TFSI]^–^, and [BF_4_]^–^ for a few applied potential differences and pore width 0.8 nm. Reproduced with permission from ref (194). Copyright 2018 Elsevier.

Neal et al.^192^ studied ion selectivity in unpolarized meso and micropores for a mixture of [EMIM]^+^ cations and [TFSI]^–^ and [BF_4_]^–^ anions. These authors modeled [EMIM]^+^ and [TFSI]^–^ as charged hard spheres of the same diameter 0.5 nm and [BF_4_]^–^ as a smaller charged sphere of diameter 0.3 nm. They found that micropores were highly selective, preferring the smaller [BF_4_]^–^, as indicated by the selectivity parameter (in analogy with binding selectivity in chemical reactions)
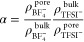
56where ρ_γ_^pore^ and ρ_γ_^bulk^ are densities of  anions in the pore and bulk, respectively. Neal et al. reported high values of α in micropores (high [BF_4_]^–^ concentrations) and α ≈ 1 in mesopores (Figure 14b). We note that these authors used a dielectric constant ε = 1, which does not take into account the electronic and other degrees of freedom, not considered explicitly in their DFT model; increasing ε reduced the effect.^192^ Interestingly, the surface charge at zero applied potential difference was practically the same for micro- and mesopores (Figure 14c). Neal et al.^192^ claimed that this effect is due to the oscillatory structure of IL inside pores. A likely reason is the similarity of the IL structure at the pore walls in both cases, giving similar surface charges. However, the bulk region developed inside mesopores reduced α compared to micropores.

In follow-up work, Neal et al. studied ion selectivity^193^ and capacitance^194^ in polarized pores. They found that the selectivity could increase by a few orders of magnitude under applied voltage, meaning an abundance of smaller [BF_4_]^–^ anions in micropores (Figure 14d). This behavior is due to entropic effects arising from the smaller-sized [BF_4_]^–^ adsorbed into the pore at positive voltages. For negative voltages, the reason is not so apparent. In another work, Neal et al.^194^ showed that there is an optimal bulk fraction of [BF_4_]^–^ maximizing the integral capacitance (Figure 14e,f). While the increase in capacitance at low molar fractions is due to the increased number of smaller anions, which increases the surface charge because of entropic effects, the reason for the capacitance decrease at large molar fractions of [BF_4_]^–^ (Figure 14f) is not straightforward.

#### 5.2.6 Phase Transitions

**Figure 15:**
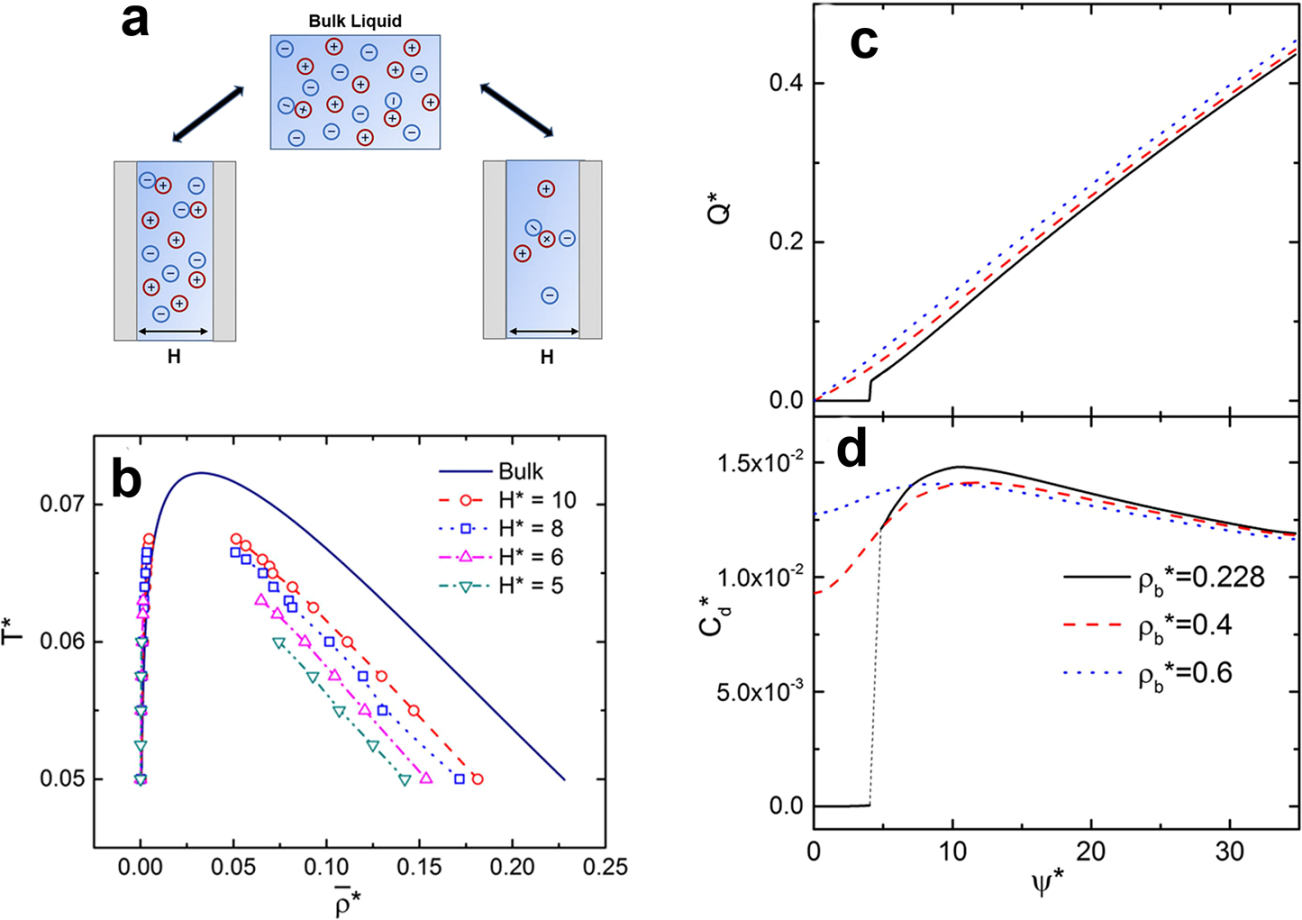
“Capillary evaporation” transition in confined IL from DFT calculations. (a) Schematic of slit-shaped pores filled with an ionic liquid in the low-density (gas) and high-density (liquid) phases. (b) Phase diagrams of an ionic liquid in bulk and under confinement in the temperature-density plane where T^*^ = λ_B_/*d* and ρ^*^ = ρ*d*^3^, and *d* is the ion diameter. The ionic liquid is in the homogeneous state above critical point *T*_c_ (the tip of the bell-shaped curves, not indicated) and phase separates into a low and high density phase below *T*_c_, in analogy to liquid–gas diagram of simple fluids. The slit width H^*^ is called to the ion diameter *d*. (c,d) Accumulated charge and differential capacitance as functions of applied potential difference for a few values of bulk ion density ρ^*^. Slit width H^*^ = 3.5 and temperature T^*^ = 0.05. Reproduced with permission from ref (195). Copyright 2017 AIP Publishing.

Similarly to simple fluids, an IL can separate into a high-density (liquid) and low-density (vapor) phases. Liu et al.^195^ have used classical DFT to study the behavior of confined ionic liquids in the vicinity of the phase coexistence (Figure 15a,b). Figure 15b compares phase diagrams in bulk and under confinement, demonstrating topologies similar to simple fluids. There is a region of temperatures above the critical temperature *T*_c_ (≈0.72 in bulk, see Figure 15b), where the IL is homogeneous at any density. For temperatures below *T*_c_ and bulk densities inside the bell-shaped curves of Figure 15b, the IL phase separates into a high-density and a low-density phase. Figure 15b shows that confinement reduces the region where the IL phase separates and shifts the critical point to lower values, which is consistent with earlier theoretical work^196^ and DFT calculations.^197^

In Figure 15c,d, we show the charge and differential capacitance as functions of applied potential difference for conditions under which the IL concentration inside a pore practically vanishes (capillary evaporation). By applying a voltage to the pore, the in-pore IL density and the accumulated charge abruptly increase, manifesting a first-order phase transition. Clearly, at the transition, the differential capacitance experiences a jump. In follow-up work, Liu and Wu^198^ reported similar effects also for size-asymmetric ILs. Phase transitions in ILs confined in conducting slit-shaped pores have been found earlier for ultranarrow pores using continuum mean-field theory^23,76^ and lattice models^40,43,77^ (see section 4.2.3), and Monte Carlo^81,82^ and molecular dynamics^80^ simulations. More recently, Cruz et al.^199^ found a “capillary ionization” transition between IL-rich and IL-poor phases close to the IL-solvent demixing in bulk,^199^ but these authors used a simple mean-field theory.

#### 5.2.7 Reversible Heat Production

**Figure 16:**
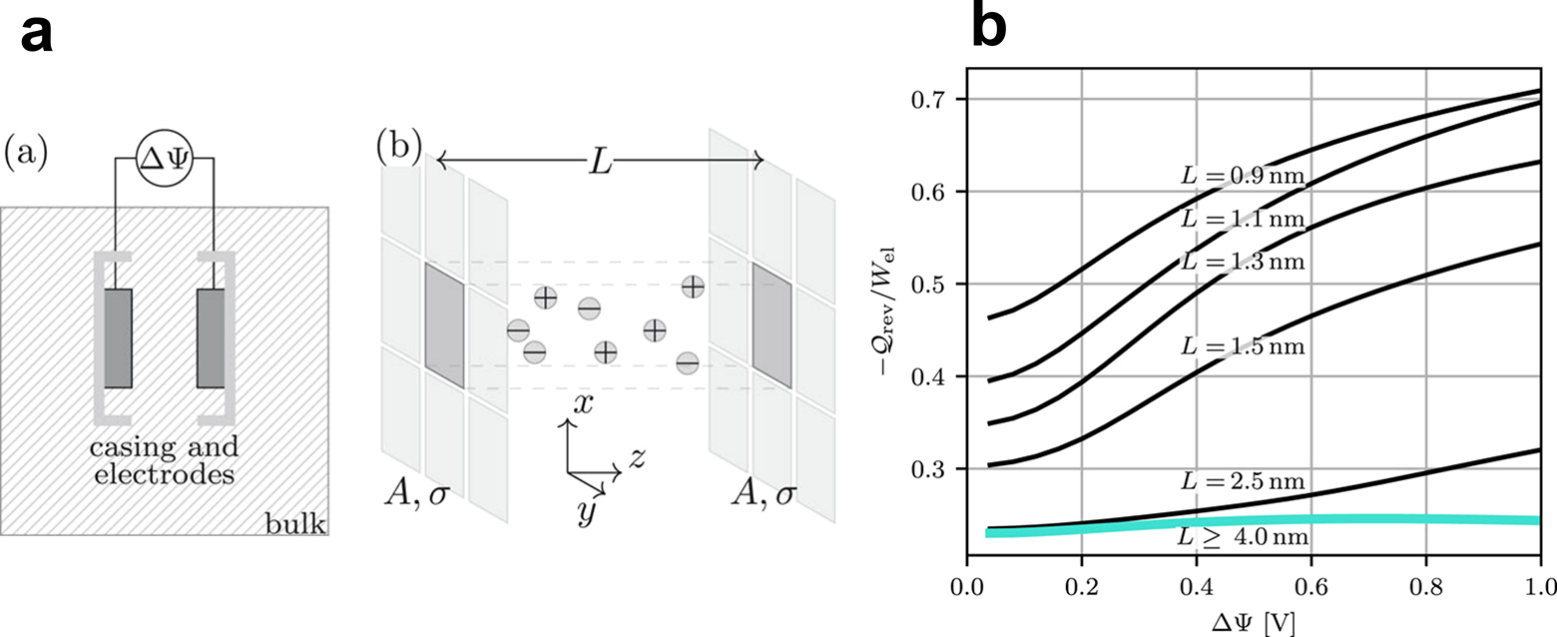
Reversible heat production. (a) Schematic of a supercapacitor with two porous electrodes (left) and of a single nanopore of width *L* (right). The potential difference ΔΨ is applied between the electrodes. (b) Voltage-dependence of the reversible heat production  expressed in terms of the work done to the system (= stored energy density) for a few values of the width of slit-shaped nanopores. Reproduced with permission from ref (159). Copyright 2021 AIP Publishing.

An isothermal charging of a nanopore produces a reversible heat flow in or out of the system,^200,201^ which is given by
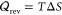
57where Δ*S* is the entropy change during charging. Thus, measuring *Q*_rev_ grants the direct access to the entropic contribution to the grand potential cost of charging. Janssen et al. have measured  for microporous carbons filled with aqueous NaCl and found this contribution to be about 25% for a step-voltage charging at 1 V.^202^ Using thermodynamic relations and applying classical DFT to charged hard spheres and slit pores, Glatzel et al.^159^ calculated the ratio  of reversible heat flowing into nanopores of various widths, where
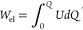
58is the electrical work done to the system (=energy stored by the system, cf. eq 4). In line with experiments, these authors found that  is negative, i.e., the positive heat flows out of the nanopore and increases with increasing the applied voltage and decreasing the pore size (Figure 16). For ultranarrow pores and high voltages, the heat flow becomes almost comparable to *W*_el_. For pores wider than about 4 nm,  becomes relatively small and pore-size independent, because in this case the double-layers practically do not overlap. In follow-up work, Pelagejcev et al.^177^ showed that hydration shell, ion-size asymmetry, and steric ion–solvent interactions could significantly affect the reversible heat production. They even found a situation where one electrode could be heated and the other one cooled during the charging of a two-electrode system.^177^

#### 5.2.8 Ion Dynamics in Nanopores

**Figure 17:**
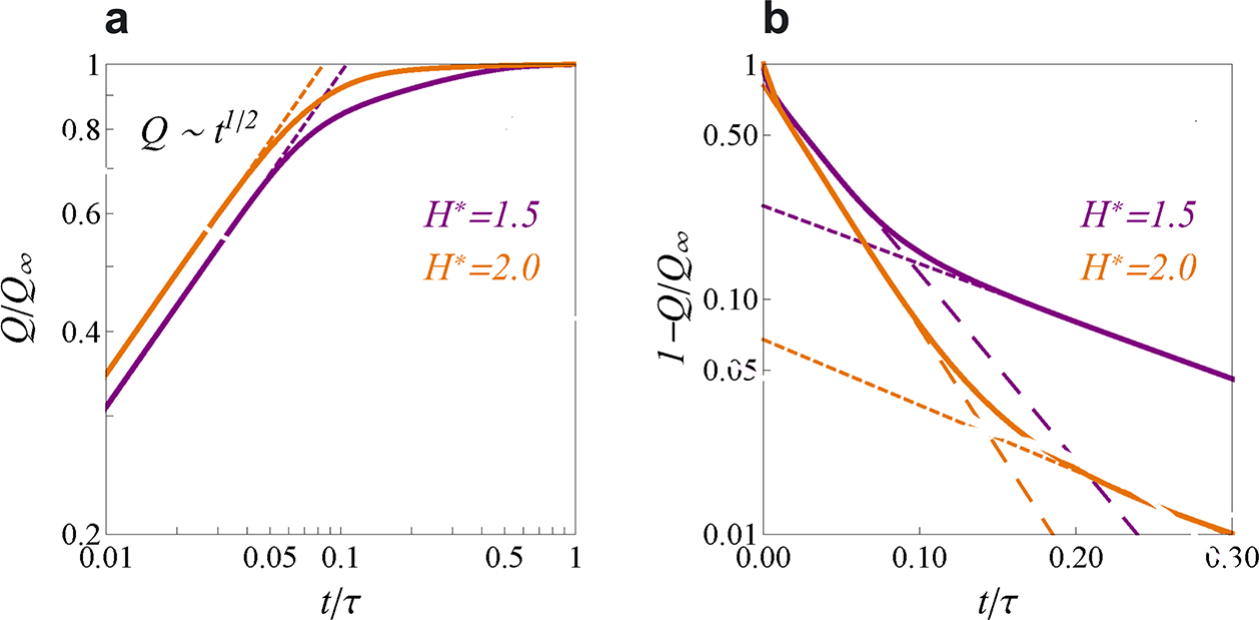
Dynamics of charging slit nanopores from time-dependent DFT. (a) Total charge accumulated in a pore as a function of time for two pore widths *H*/*d* = 1.5 and *H*/*d* = 2, where *d* is the ion diameter, assumed the same for cations and anions. (b) Charging in the linear-log scale showing two exponential regimes denoted by long-dashed and short-dash lines. Initially, the charge grows as a square root of time and follows by two exponential regimes at intermediate and late times, in line with ref (112) (section 4.5.1, Figure 9) and molecular dynamics simulations^113,125^ (section 6.4.2, Figure 30). In all plots, applied potential *U* = 10 and , where  is the pore length. Reproduced with permission from ref (174). Copyright 2022 MDPI under CC BY (https://creativecommons.org/licenses/by/2.0/).

Dynamical DFT has been applied to confined ILs only recently. Aslyamov et al.^174^ developed a dynamic model for an electrolyte inside slit-shaped nanopores based on the time-dependent classical DFT. They found a square-root and two exponential regimes of charging (Figure 17), as previously predicted by the mean-field model^112^ (section 4.5.1, Figure 9) and molecular dynamics simulations^113,125^ (section 6.4.2, Figure 30). Their calculations showed that the slowest exponential regime is associated with co-ion desortion and plays a significant role only for pores narrower than two ionic diameters. We note that these authors did not predict the linear regime (see the inset in Figure 30c) because their model did not consider the bulk electrolyte.

Tomlin et al.^173^ used a similar approach and derived a “reduced-order limit model” from time-dependent DFT to investigate the impedance response of ILs confined in long slit-shaped pores. Furthermore, they demonstrated that electrostatic correlations are vital for a physically correct description and could drastically modify the high and low-frequency response times for pores narrower than two ion diameters. They found that a size-asymmetric ionic liquid and ultranarrow pores (of width close to the smallest ion) could help avoid a trade-off between power and energy density, thus simultaneously optimizing both quantities.

This issue has been recently scrutinized by Qing and Jiang,^175^ who applied the dynamical DFT to study charging with size-symmetric and asymmetric ILs. In line with ref (173), their calculations indicated that size-asymmetric ionic liquids may simultaneously increase power and energy density. They found that for symmetric ILs, the charging time τ_charging_ and the capacitance are positively correlated, while for asymmetric ILs, τ_charging_ is inversely proportional or independent of the capacitance, depending on the bulk ion concentration. The reason for this behavior is not clear. The authors of ref (175) also reported on transient “kinetic charging inversion” occurring for asymmetric ILs, where the sign of the charge at short times was opposite to the sign of the charge in equilibrium. A similar charging inversion was reported earlier for planar electrodes with nanoscale electrode separation (around three ion diameters) by Jiang et al.,^203^ who related this behavior to the oscillatory structure of an ionic liquid at the confining walls.

Lian et al.^204^ combined dynamical DFT and the Navier–Stokes equation to study ionic conductivity inside nanopores. They found that the conductivity depends on the applied gate voltage and behaves nonmonotonically with the slit width, showing a maximum at about 1 nm. For a relatively high gate voltage (0.5 V), the conductivity exhibited an oscillatory structure, which the authors related to ion layering in confinement. Modeling the same system within the Poisson–Boltzmann approach predicted neither layering nor oscillations in the conductivity.^204^

## 6 Simulations

### 6.1 Types of Simulations and Challenges

#### 6.1.1 Equilibrium Simulations

Monte Carlo (MC) and molecular dynamics (MD) simulations are widely used to investigate RTILs under confinement. Simulations provide exact results for specific models allowing the study of highly complex systems (see examples below sections 6.2–6.4). Molecular simulations align perfectly with the nanometer length scales, making it possible to perform truly “in silico” experiments. Both MC and MD simulations have been used widely in the area of electrochemical energy storage and dynamics of the electrical double layer formation (see, e.g., ref (184) for an early perspective and sections sections 6.2–6.4). Canonical (*NVT*) and Grand Canonical (μ*VT*) ensembles are often used. The latter is suitable for applications discussed below, such as dynamic charging effects or electrotunable friction, because it allows modeling the ion exchange between confined/bulk systems that emerge naturally in these processes. Grand Canonical MC has been used to investigate charged hard spheres (restrictive primitive model, RPM), addressing anomalous properties of ionic liquid-based supercapacitors^49^ and providing support to the predicted superionic state (see section 3).

Implementations of Grand Canonical MD (GCMD) simulations might involve the use of fluid reservoirs,^182,206−211^ which results in large systems sizes, needed to accommodate both the reservoir and the confined region, as well as explicit particle insertion/deletion, which allows for the simulation of smaller systems. The latter simulations are more suitable for adsorption calculations, as the insertion/deletion process involves Monte Carlo steps that perturb the system dynamics. Canonical simulations are in general computationally more efficient as they focus on the confined region only.^212−216^ Systematic analyses of nanopores of different widths must be performed to ensure the chemical potential of the liquid is constant upon varying the pore width. Constant chemical potential can be achieved using the GCMD simulations if dynamic information is required (see section 6.1.2 for a discussion of this approach).

One major issue of molecular dynamics simulations is time-scale limitations, which can be a significant problem in nanoconfined spaces, as the diffusion coefficient decreases 1 order of magnitude from the bulk to the surface.^216^ Hence long simulation times spanning 10–100 nanoseconds are required. While these time scales are accessible with state of the art supercomputers and Verlet integration algorithms, they can still be challenging when considering polarizable and fully atomistic molecular force fields,^217^ as these may require shorter timesteps, ∼0.5 fs, than nonpolarizable force fields, to integrate the fast degrees of freedom emerging from intramolecular interactions. Multiple-step time-reversible algorithms^218,219^ furnish a route to accelerate the simulations significantly, particularly in systems involving very different intramolecular and intermolecular time scales. Such algorithms have been successfully employed to simulate RTILs under confinement.^220^

Molecular dynamics simulations often rely on employing thermostats to maintain the system’s average temperature. The Nosé–Hoover chain thermostats,^221,222^ and canonical v-rescale thermostats^223^ are popular choices because they reproduce the canonical ensemble fluctuations. The Berendsen thermostat^224^ also allows efficient thermostatting if fluctuations are not required. All these thermostats have been used in a number of simulations of RTILs under confinement.^182,212,213,220,225−227^

#### 6.1.2 Nonequilibrium Simulations

**Figure 18:**
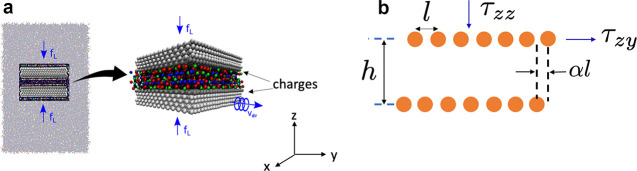
Grand Canonical nonequilibrium molecular dynamics (GC-NEMD) simulations. (a) Simulation shapshot of a typical GC-NEMD setup used to investigate electrotunable friction of ionic liquids. The arrows next *f*_L_ indicate the direction of the load applied to the solid slabs. The atomic layer in direct contact with the ionic liquid contains point charges that can be varied to define a specific surface charge density. Adapted with permission from ref (233). Copyright 2020 American Chemical Society. (b) Definitions of the relevant thermodynamic quantities for the confined fluid. See text for further details.

Nanofriction phenomena have been often interpreted using the Prandtl–Tomlinson model,^228^ which considers a point mass sliding on a one-dimensional sinusoidal potential, and the Frenkel–Kontorova^229^ model, which considers a 1D chain of atoms coupled by harmonic oscillators, and subjected to a sinusoidal potential.^228^ These simple models provide a conceptual framework to investigate frictional phenomena at the nanoscale (nanotribology), however, it is not clear how to apply these models or their extensions to describe electrotunable (EL) lubrication in ionic liquids. Because of this, nonequilibrium simulations have been widely used in the context of RTIL lubricity. So far, theoretical developments have focused on MD simulations, mainly using the Grand Canonical nonequilibrium MD (GC-NEMD) method.^206,207,209,210,226,230–234^ In GC-NEMD, the confined region is in contact with a bulk reservoir that fixes the chemical potential of the confined liquid (see Figure 18a). The confined liquid is subjected to a normal load and shear. Hence, the GC-NEMD implementation mimics the SFA and AFM experiments, allowing the exchange of molecules in the confined regions to respond to shear and changes in the applied load, *f*_L_, and surface charge. Friction forces in ionic liquid films between uncharged surfaces have also been computed using a constant number of particles in the confined region.^215,225,236^ The GC-NEMD approach offers the advantage of simulating explicitly “squeeze out” processes upon increasing normal loads, allowing the study of the mechanisms determining film thinning and providing a direct connection with AFM and SFA experiments.

The relevant thermodynamic ensemble of the GC-NEMD method is the grand isostress ensemble. The grand isostress potential is derived from the fundamental relation governing the internal energy, *U*,^237,238^
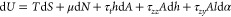
59where *T*, *S*, and *N* are the temperature, entropy, and the number of particles, respectively, τ_*t*_ = 0.5(τ_*xx*_ + τ_*yy*_)/2 is the transverse stress, *A* is the area of the confined region, *h* is the distance between the surfaces, τ_*zz*_ is the normal stress acting on the slabs, τ_*zy*_ represents the work of shearing, and *l* and α are the unit cell parameter and the registry, i.e., the relative shift of the slabs with respect to each other (see Figure 18b). For instance, the two slabs are in full registry when α = 0. The fundamental relation for the grand isostress ensemble follows from the application of two Legendre transformations, Ω = *U* – *TS* – μ*N* and Φ = Ω – τ_*zz*_*Ah*, where Ω and Φ represent the grand potential and the grand isostress potential, respectively, which gives
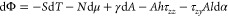
60and γ is the interfacial tension of the liquid–solid interface. The grand isostress potential has been used to evaluate the stability of thin films as a function of film thickness.^237^

The actual implementation of the shear process in the GC-NEMD method involves attaching a virtual spring with force constant *k* to a slab that moves at a constant speed *v*_dr_ (Figure 18). The friction force, *F*, is calculated following the spring extension, namely *F*(*t*) = −*k*(*r*(*t*) – *v*_dr_*t*), where *r*(*t*) is the center-of-mass position of the slab at time *t* (see Figure 18a). The time-dependent stress can be calculated using *F*(*t*)/*A*, where *A* is the area of the sliding slab. During the shear process, a fraction of the mechanical energy is lost into heat, increasing the temperature of the entire system. Thermostats are applied to the substrates to prevent the system’s heating.^239^ The thermostatting of both the substrate and fluid can trigger nonequilibrium transitions^240^ and, therefore, should be avoided.

**Figure 19:**
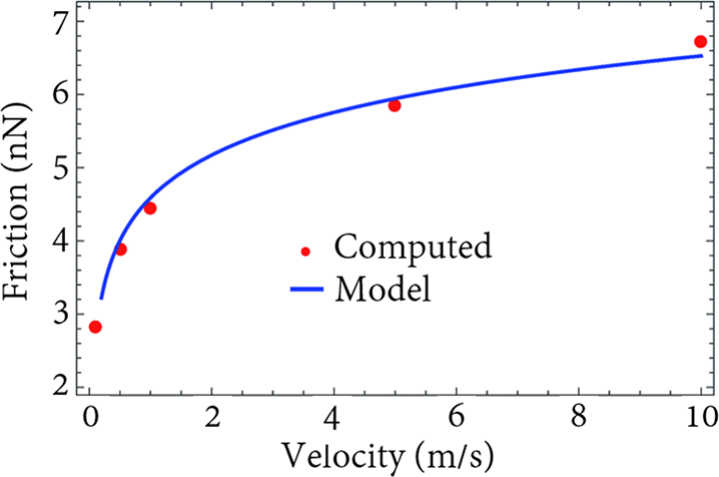
Variation of the friction with sliding speed. Friction force as a function of speed. Reproduced with permission from ref (242). Copyright 2017 Royal Society of Chemistry. The full line in panel is a fitting to eq 61.

GC-NEMD simulations involve typical system sizes of about 10^4^ atoms, which are needed to model the confined and reservoir regions. The simulated sliding speeds usually vary between 10^–2^ and 100 m/s,^215,225,226,241^ much higher than those employed in the AFM and SFA but of the order of the speed using in state-of-the-art tribometers in industrial applications. The friction force in lubricated systems usually increases logarithmically with the sliding speed (Figure 19). This dependence has been modeled using an Eyring activation model,^242^ resulting in the following equation for the friction stress
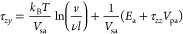
61where *V*_sa_ and *V*_pa_ represent the stress activation and pressure activation volumes of molecules attempting jumps of length *l* and frequency ν. *E*_a_ is the activation energy in the absence of normal pressure, τ_*zz*_. This model describes the friction force dependence with sliding speed, and from the analyses of simulation data one can extract activation energies.^242^

#### 6.1.3 Force Fields

**Figure 20:**
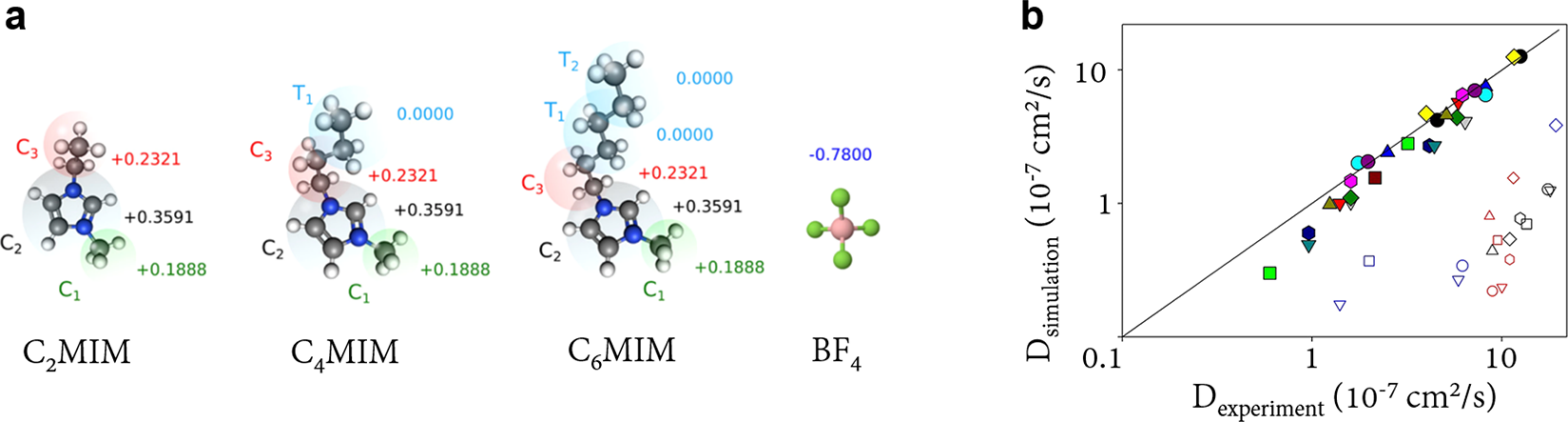
Force fields and benchmarks of dynamics properties of RTILs. (a) Examples of coarse grained and atomistic models of RTILs. Reproduced with permission from ref (233). Copyright 2020 American Chemical Society. (b) Comparison of simulated and experimental diffusion coefficients for imidazolium, pyrinidium, pyrrolinidium, alkylamonium, and piperidonium RTILs. The open and filled symbols represent results obtained with nonpolarizable and polarizable models, respectively. Reproduced with permission from ref (217). Copyright 2019 American Chemical Society (https://pubs.acs.org/doi/10.1021/acs.chemrev.8b00763 further permissions related to the material excerpted should be directed to the ACS).

The force field, *U*(*r*_1_, ..., *r*_*N*_), is a key ingredient to perform any simulation. For RTIL under confinement, different levels of sophistication have been considered. So called primitive models map the complex geometries of the ions into single spheres and provide the simplest representation. These models are helpful to test theoretical approximations (see section 4) by furnishing exact data for simple theoretical models. Coarse grained models (CG) (see Figure 20a) incorporate chemical specificity and map several atoms into beads that interact through intramolecular interactions.^212,243−246^ All atom models (Figure 20a) provide the highest level of atomistic description of the ion structure,^247−249^ however, the simulation of these models is computationally expensive.

The general form of the interaction potential is described by intramolecular and intermolecular terms *U* = *U*_intra_ + *U*_inter_, with the intramolecular part defined by bond, bending, and diheadral terms. For the Optimized Potentials for Liquid Simulations (OPLS) force field,^250^ which has been applied extensively to investigate ionic liquids,^247^ the functional form is,^250^
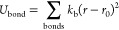
62
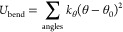
63
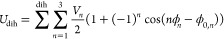
64where *k*_α_ and *V*_α_ represent force constants, *r*_0_ and θ_0_ the equilibrium bond lengths and angles, ϕ the dihedral angles, and ϕ_0,*n*_ phase angles.

The intermolecular pair interactions are modeled with a combination of Coulombic and dispersion interactions, with the Lennard-Jones potential being the most common choice for the latter, i.e.
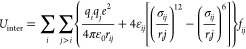
65where  is defined in terms of the effective diameters of the atoms *i* and *j*, and the cross interaction (*i* ≠ *j*)  in terms of the interaction strength between atoms of type *i* and *j*. *f*_*ij*_ is a “fudge” factor equal unity for intermolecular interactions and *f*_*ij*_ = 0.5 for 1,4-dihedral interactions. eqs 62–65 are the basis of nonpolarizable empirical force fields and widely used to model ionic liquids fully atomistically. The relevant terms in CG models are eqs 62–65), while only eq 65 is needed for primitive models.

*Ab initio* molecular dynamics (AIMD) simulations allows “on-the-fly” computations of intermolecular forces by solving the Schrödinger equation during a simulation. AIMD are also applicable to RTILs and provide an approach to investigate reactivity, an aspect that cannot be addressed using classical simulations straightforwardly. Moreover, the polarization effects discussed above are included by default because the electronic problem is solved every time step. AIMD often targets simulations sizes of tens to hundreds of ions pairs over 100 ps time scales. These simulations have focused mostly on structural data.^251−254^ The systems sizes used in the early simulation warrant some examination due to finite size effects and how they affect the liquid dynamics, in particular, the self-diffusion coefficient (see section 6.1.5). Generally, *ab initio* methods are useful to parametrize intramolecular potentials.^255^

#### 6.1.4 Electrostatic Interactions

Electrostatic interactions play a crucial role in determining the structure and dynamics of RTILs. The 3D Ewald summation method and different implementations, such as PME^256^ or PPPM,^257^ provide an efficient and accurate approach to compute the electrostatic interactions. Confinement breaks the bulk symmetry, at least in one direction, and requires special consideration. This issue has motivated modifications of the standard 3D method. The Ewald method was extended to systems that are periodic in two dimensions and finite in the third dimension^258−260^ and employed to investigate capacitance in nanoporous carbon electrodes^261^ and the standard parallel plate capacitor.^258^ The main message from these and other studies is that the indiscriminate use of the 3D Ewald method in 2D systems can lead to inaccurate results. To address these issues, Yeh and Berkowitz^262^ proposed a correction to compute the electrostatic interactions of charged systems confined in slab geometry. The correction is easy to implement in the standard 3D Ewald codes. Following the Ewald method, the electrostatic energy of a system consisting of *N* particles is given by
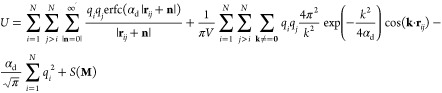
66where damping parameter α_d_, image cell number **n**, and reciprocal vectors **k** are adjusted to ensure good numerical accuracy and computational efficiency, and *S* is a factor taking into the shape of the simulation box. The correction to the 3D Ewald method emerges from the summation geometry, *S*(**M**), which, in turn, depends on the total dipole moment of the simulation box, **M** = ∑_*i* = 1_^*N*^*q*_*i*_**r**_*i*_. For the commonly used “tinfoil” boundary conditions, namely, the simulation box is surrounded by a medium with infinite dielectric constant, *S*(**M**) = 0. For rectangular plates, such as those present in the slab geometry, the shape term is^263^
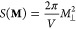
67where *M*_⊥_ is the dipole moment component perpendicular to the slab plane. This term contributes a force to each charge, *i*, given by
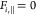
68
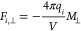
69where ∥ represents the component parallel to the slab plane. The equations above can be used along with the standard 3D Ewald method to simulate 2D systems because eq 69 subtracts the interactions emerging from the Ewald method when replicating the system in 3D. This 3DC method is very efficient and converges to the correct 2D solution when the slab is surrounded by vacuum and the box length in the direction perpendicular to the slab is at least *L*_⊥_ = 3*L*_∥_. This approach has been used in a number of *NVT* simulations of aqueous solutions and RTILs confined between parallel slabs.^216,264−267^ The 2D and 3D Ewald methods have also been extended beyond point charges, to model Gaussian charges, which are relevant in the modeling of metallic surfaces.^268^ Recently, Coretti et al.^269^ discussed the implementation of electrostatic interactions to investigate electrochemical interfaces in the framework of the Ewald summation method. The approach discussed in that work accounts for the electrode geometry, the polarizabilty of electrolytes and metallic electrodes.

#### 6.1.5 Challenges with Modeling Ion Dynamics

Fully atomistic and CG simulations provide an accurate account of the thermophysical properties of ionic liquids. However, modeling the diffusion coefficients of ionic liquids is challenging. Simulation of small systems (often used in fully atomistic simulations either classical or *ab initio*) may result in undesirable finite size effects associated with long-range hydrodynamic interactions. Yeh and Hummer and Dünweg and Kremer^270,271^ derived an equation based on a hydrodynamic model of a particle immersed in a solvent of viscosity η, with the particle moving on a periodic simulation cell,
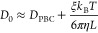
70where *D*_0_ is the diffusion coefficient for an infinite (macroscopic) system, *D*_PBC_ is the diffusion coefficient obtained in a simulation box of length *L* and ξ = 2.837297 for a periodic cubic box. This equation has been tested recently in molecular dynamics simulations of reactive force fields of water^272^ and to obtain shear viscosities from the finite size effects of *D*_PBC_, for a variety of fluid mixtures, including one RTIL ([BMIM] [NTf_2_]).^273^ The finite size correction alone cannot account for the large underestimation of the diffusion coefficient observed in several RTILs force fields, which is reflected in an underestimation of the simulated ionic conductivities (about 1 order of magnitude) of imidazoium RTILs.^274^

Hydrodynamic effects also influence the lateral diffusion coefficients, *D*_∥_, of fluids under confinement. Simonnin et al.^275^ have discussed this problem recently and derived the following expression using the Stokes equation in a slit pore
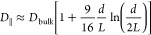
71where *D*_bulk_ is the diffusion coefficient in the bulk, *d* is the diameter of the confined molecules, and *L* is the slab width. This continuum hydrodynamic equation models accurately the dependence of the lateral diffusion coefficient of Lennard-Jones fluids with *L*. The correction is important to account for the dynamics of confined fluids in nanopores with very large lateral sizes (much larger than the pore width). The impact of such finite size effects become less important in narrow pores, where the molecular structure of the confined fluid is important.^276^

The large underestimation of diffusion coefficients has motivated a large number of works aimed to developing polarizable models, as a speed up of ion dynamics upon including polarization effects was observed in the initial studies.^217,277−281^ The polarization models built upon the fluctuating charge model, the classical Drude oscillator model, induced point dipoles and higher-order induced moments. Bedrov et al.^217^ provide an excellent introduction and discussion to polarizable force fields, and we will not discuss these further. Technical aspects connected to the computation of electrostatic interactions using polarizable ions were discussed by Coretti et al.^269^ We note, however, that good agreement between simulation and experiment was obtained using nonpolarizable models by reparametrizing atomistic force fields,^248,282^ as well as using coarse grained models.^212,244,245^

The accurate modeling of the dynamic properties is also desirable to reproduce the right times scales associated with the cooperative behavior of RTILs and dynamical processes such as lubrication in nanoscale gaps. The characteristic dynamic properties of ionic liquids impose a lower limit to the simulation time scales needed to obtain well equilibrated trajectories. RTILs feature high viscosities (30–100 times higher than that of water), low diffusion coefficients (2 orders of magnitude lower than water, 10^–11^m^2^s), and therefore long relaxation times (greater than 20 ns). Hence, long trajectories 0.1–1.0 μs are often needed in state of the art simulations. These time scales can be accessed easily using primitive and coarse grained models, at a fraction of the computational cost required by atomistic models, but also at the cost of ignoring atomistic details. The long-time scales are needed to analyze RTILs with high molecular weights and to investigate the mesoscopic structure of RTILs, which emerges from the amphiphilic character of the molecular ions and the self-assembly of the molecules into hydrophilic (polar) and hydrophobic (nonpolar region chain) domains. Such mesoscopic structures were observed using atomistic and CG models.^243,245,283^ For obvious reasons, these structures cannot be modeled using primitive models, as the amphiphilic character of the ions is not included. The description of rotational diffusion and rotational degrees of freedom, particularly aromatic heterocycles under confinement, is relevant, as it can drive the in-plane structural order, hence justifying the adoption of more detailed models.^233,284,285^

#### 6.1.6 Constant Potential Simulations

Electrons of a conducting metallic surface respond to the motion of nearby ions to maintain a constant potential at the surface by dynamically adjusting an electrostatic field that, in turn, affects the behavior of ions. For ions confined into narrow pores, such metal wall–ion interactions, lead to an exponential screening of electrostatic interactions between the ions (section 3.1, Figure 2). For simple confining geometries, there are analytical expressions for interionic interaction potentials (see, e.g., eqs 9 and 11) that one can directly employ in MD or MC simulations. Such approaches have been used to study ionic liquids in infinitely extended slit^22,42,49,70,76,77,286−288^ and cylindrical^44,60,62,289,290^ pores. A similar strategy was taken by Girotto et al.^291^ and dos Santos et al.,^292^ who employed periodic Green functions to derive an efficient algorithm for simulations of ionic fluids in polarizable slit confinement. They applied this algorithm to simulate ions confined between two flat electrodes using lattice models.^58,59^

Because analytical expressions for interionic potentials can be obtained only for simple geometries (e.g., eqs 9 and 11), alternative approaches are necessary for more generic or more complex porous structures. Perhaps the most straightforward one is to solve the Poisson equation with appropriate (metallic) boundary conditions to find the electrostatic field acting on the ions.^264^ This procedure must be performed at every simulation step, which slows down simulations. Other approaches rely on putting countercharges alongside an electrolyte–electrode interface and adjusting their values each simulation step to reflect metal polarizability. Siepmann and Sprik have introduced variable-charge Gaussian charges, whose magnitude is changed on the fly according to a variational procedure designed to maintain the constant potential on an electrode.^259,294,296^ This method has been used in several MD simulations studies, particularly for modeling electrodes with complex nanoporous networks, such as carbide-derived carbons.^48,51,116,261,298−301^ Recently, it has been implemented in the open-source software package Metalwalls.^269,302^ A similar method has been implemented in the well-known LAMMPS simulation package^265^ and more recently extended to fully periodic systems.^304^ Most recently, Ahrens-Iwers et al.^305^ developed a package ELECTRODE for LAMMPS for efficient simulations of solid–liquid interfaces using constant potential method, able to deal with heterogeneous and curved electrodes.

Tyagi et al. developed an induced counter charge (ICC*) algorithm that allows an arbitrary number of regions of arbitrary shapes characterized by different dielectric constants in one-, two-, and three-dimensions.^306^ The ICC* approach is implemented in the Espresso MD simulation package (https://espressomd.org) and has also been used to study charging process.^42,125,307^ Metal polarizability can also be modeled through atomic polarizability.^308,309^ Geada et al.^309^ has recently parametrized the Lennard-Jones model to simulate metallic surfaces with the Drude model, i.e., with the charges of opposite sign located at each atom and bonded through harmonic potentials. The bond constants were fitted to reproduce the atomic polarizability: refs (310) and (217), provide a critical discussions of different algorithms for constant potential simulations.

**Figure 21:**
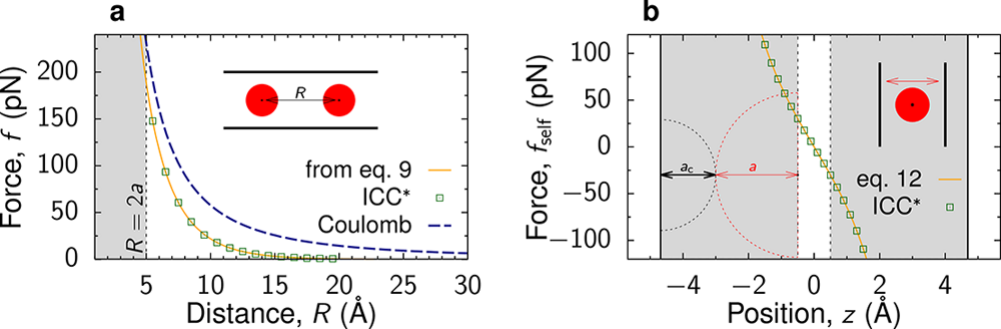
Superionic state in molecular simulations. (a) Electrostatic forces acting between two ions in a metallic slit obtained with the induced charge ICC* method for constant potential simulations (available in the Espresso MD package) and calculated directly from eq 9. For comparison, the force due to the Coulomb interaction potentials is also shown. The shaded area shows the region of steric exclusion, where *a* = 0.25 nm is the ion radius. (b) Image–charge forces acting on an ion confined into a metallic slit obtained with the ICC* method using the Espresso MD package and calculated directly from eq 12. *a* = 0.25 nm and *a*_c_ = 0.337 nm are the ion and carbon radii. The slit width *L* = 0.6 nm. Reproduced with permission from ref (42). Copyright 2017 AIP Publishing.

Figure 21 demonstrates the consistency of the analytical expressions (eqs 9 and 12) and constant-potential simulations. Figure 21a shows the force acting between two ions inside a slit obtained with the ICC* algorithm and calculated directly from eq 9. Figure 21b shows the force acting on an ion due to image–charge interactions with the slit walls obtained with the ICC* method and calculated from the analytical expression, eq 12.

#### 6.1.7 Constant-Potential vs Constant Charge Simulations

Constant-potential simulations are computationally expensive and hence there have been attempts to model constant-potential surfaces with constant charge simulations without dielectric contrast and with the potential difference determined *a posteriori* from the charge distribution. Merlet et al.^48^ have compared constant-potential and constant-charge simulations for flat and CDC electrodes. They found that constant-charge simulations do not accurately describe the behavior of ions at polarizable interfaces, particularly their dynamic properties. However, significant differences in the local ionic structure were observed only at high voltages (above 4 V), which are close to or above the limit that many ionic liquids at electrodes can withstand without oxidation or reduction. Haskins and Lawson^312^ and Ntim and Sulpizi^216^ arrived at similar conclusions. For instance, Haskins and Lawson^312^ considered planar graphite electrodes and polarizable [EMIM][BF_4_] and found that for voltages below 2 V, the electrode polarizability led to only minor increase in the ion concentration in the surface layers and did not influence the alignment of ions.^312^ This is likely because steric rather than electrostatic interactions determine the local structure at typically high ionic densities. However, they found that the effect of polarizability on differential capacitance was more substantial, with significant differences observed at all applied voltages.^312^ Breitsprecher et al.^307^ performed coarse-grained simulations of [BMIM][PF_6_] at different electrodes. For smooth walls (no atomistic structure), they found noticeable differences only for negative potentials, while for graphite-like walls, the differences were noticeable, albeit small, in the whole voltage range.

In a very recent work, Gäding et al.^314^ have investigated the effect of pore wall polarizability on ion diffusion for 1-butylpyridinium bis(trifluoromethane) sulfon-imide [BuPy][NTf_2_] ionic liquid. They found that for polarizable pore walls, the diffusion coefficient was about 20% lower for a 4.1 nm slit but more than 80% higher for a 1.65 nm slit compared to nonpolarizable (constant charge) pore walls.

Lecce et al. found that polarization of metallic surfaces had a negligible impact on frictional properties of ionic liquid films, even for films containing only a single layer of ions.^211^ They obtained fairly similar film thicknesses, friction forces, and friction coefficients in MD simulations with and without electronic polarizability of confining plates. These similarities are probably related to the minor influences of polarizability on the local ionic structure, as discussed above.

Thus, the IL structure and, consequently, friction are not significantly affected by the electrode polarizability. However, the polarizability can considerably alter the capacitive properties and particularly the dynamics of confined ILs, as discussed in this section.

### 6.2 Structure of Confined Ionic Liquids

#### 6.2.1 Ionic Liquids in Slit Nanopores

Due to their simplicity, nanoslits have been extensively used as a molecular pore model to mimic porous carbons. Slit pore models are also frequently used for analyzing experimental data, particularly in interpreting adsorption isotherms for determining pore size distributions.^18,122,316−318^ The ion packing in narrow slits differs significantly from the ion arrangement at an open electrode surface,^80,122,181,299,317^ with an IL adopting a monolayer or a bilayer structure, depending on the pore size.^122,181,318^

Wu et al.^181^ investigated the EDL structure of [EMIM][BF_4_] inside a slit pore with atomically flat walls. For polarized pores wider than two ion diameters, counterions formed two distinct layers, whereas co-ions located mainly in the pore center. For the pore size comparable to the ionic dimensions, Futamura et al.^317^ revealed through Hybrid Reverse Monte Carlo (HRMC) simulations of [EMIM][TFSI] in nanopores that the ions formed a monolayer in a 0.7 nm pore and a bilayer in a 1 nm pore. Furthermore, the Coulombic ordering, i.e., when each ion is surrounded by a solvation shell of opposite charge, was disrupted to form a structure with equally charged ion pairs.^317^ Such a non-Coulombic structure likely arises from the screening of repulsive electrostatic interactions between the co-ions due to the image charges induced by conducting pore walls, leading to a highly dense ionic structure of co-ions.^317^

**Figure 22:**
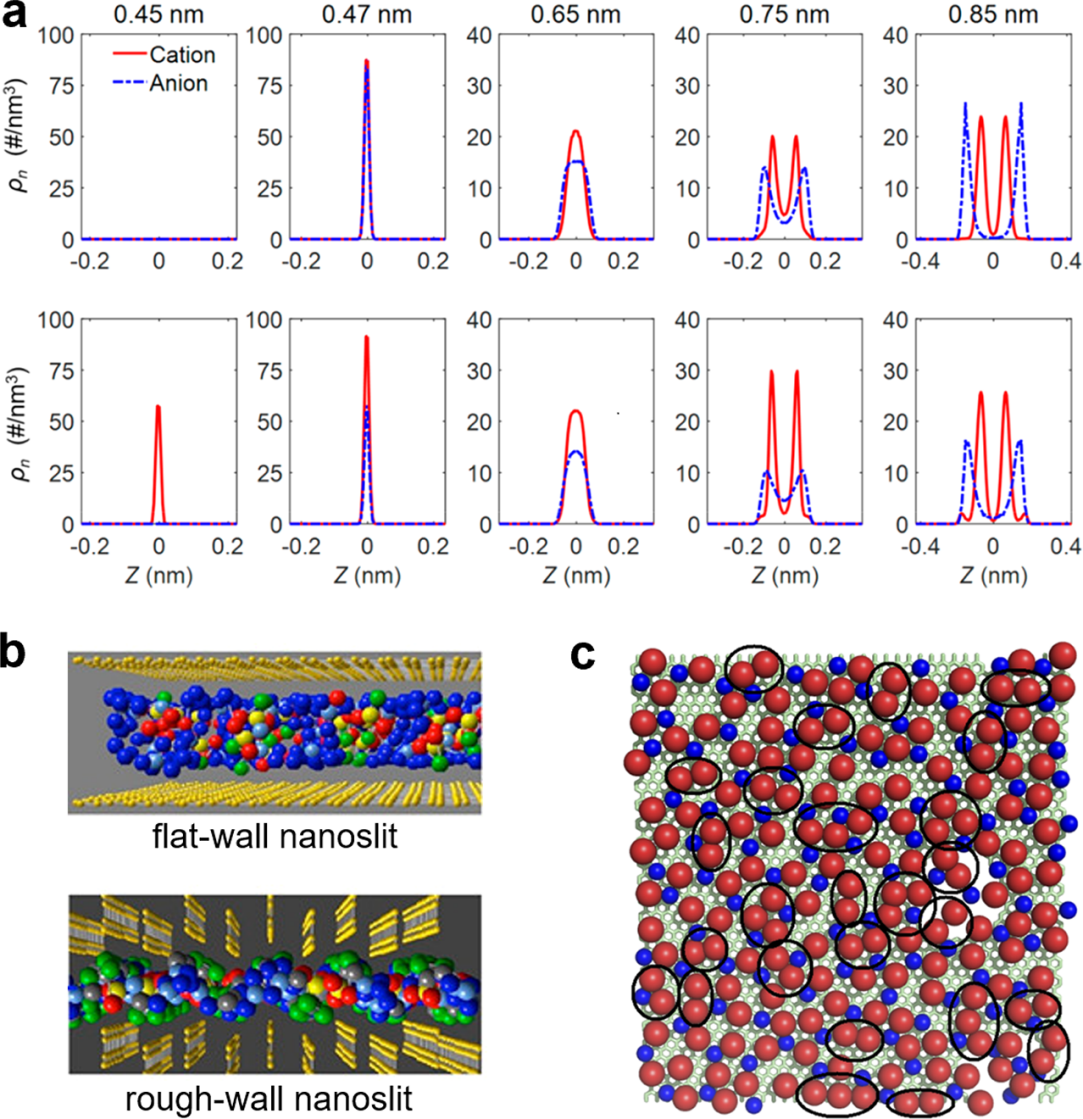
Ionic liquids in slit nanopores. (a) Number density (ρ_*n*_) of ions across slit pores with various sizes at (top) zero electrode potential and (bottom) negative polarization of 2 V. Reproduced with permission from ref (318). Copyright 2020 American Chemical Society. (b) Illustration of atomically flat and rough slit nanopores. Reproduced with permission from ref (80). Copyright 2015 American Chemical Society. (c) Snapshot of an adsorbed layer in a positively charged subnanometer pore (red and blue spheres represent the anions and cations, respectively). Reproduced with permission from ref (299). Copyright 2018 AIP Publishing.

Mo et al.^318^ found a transformation from one layer to two layers with mixing cations and anions. The transformation was observed at the pore size 0.75 nm, which is a half-integer multiple of the ion diameter (Figure 22a). They observed that such a transformation could facilitate the ion diffusion in the direction along the pore, leading to an accelerated charging process.^318^

The atomic roughness of the pore walls can also substantially affect the ion arrangement in slit nanopores. Vatamanu et al.^80^ found that atomically flat nanopores retained around 20% of co-ions and 80% of counterions in polarized pores under the applied potential difference of 3 V. In contrast, more than 99% of counterions were located at both positively and negatively polarized rough nanopores at the same voltage, leading to the enhanced integral capacitance (Figure 22b).

Mendez-Morales et al.^299^ explored the interaction of ions located on the two sides of a graphene sheet. As shown in Figure 22c, simulation results demonstrated that due to the formation of a highly localized image charge on the carbon atoms, the ions of the same sign tended to adsorb in front of each other across the graphene plane.^4000^ A similar effect was observed with quantum density functional calculations showing that the ions of the same sign attract each other when placed inside and outside a single-wall carbon nanotube. However, this phenomenon was suppressed in larger pores when the IL adopted a bilayer structure between the graphene sheets.^299^ More recent work showed that such interpore ionic interactions could profoundly affect charge storage, enhancing or reducing the stored energy density, depending on the sign of like-charge interactions.^5000^

**Figure 23:**
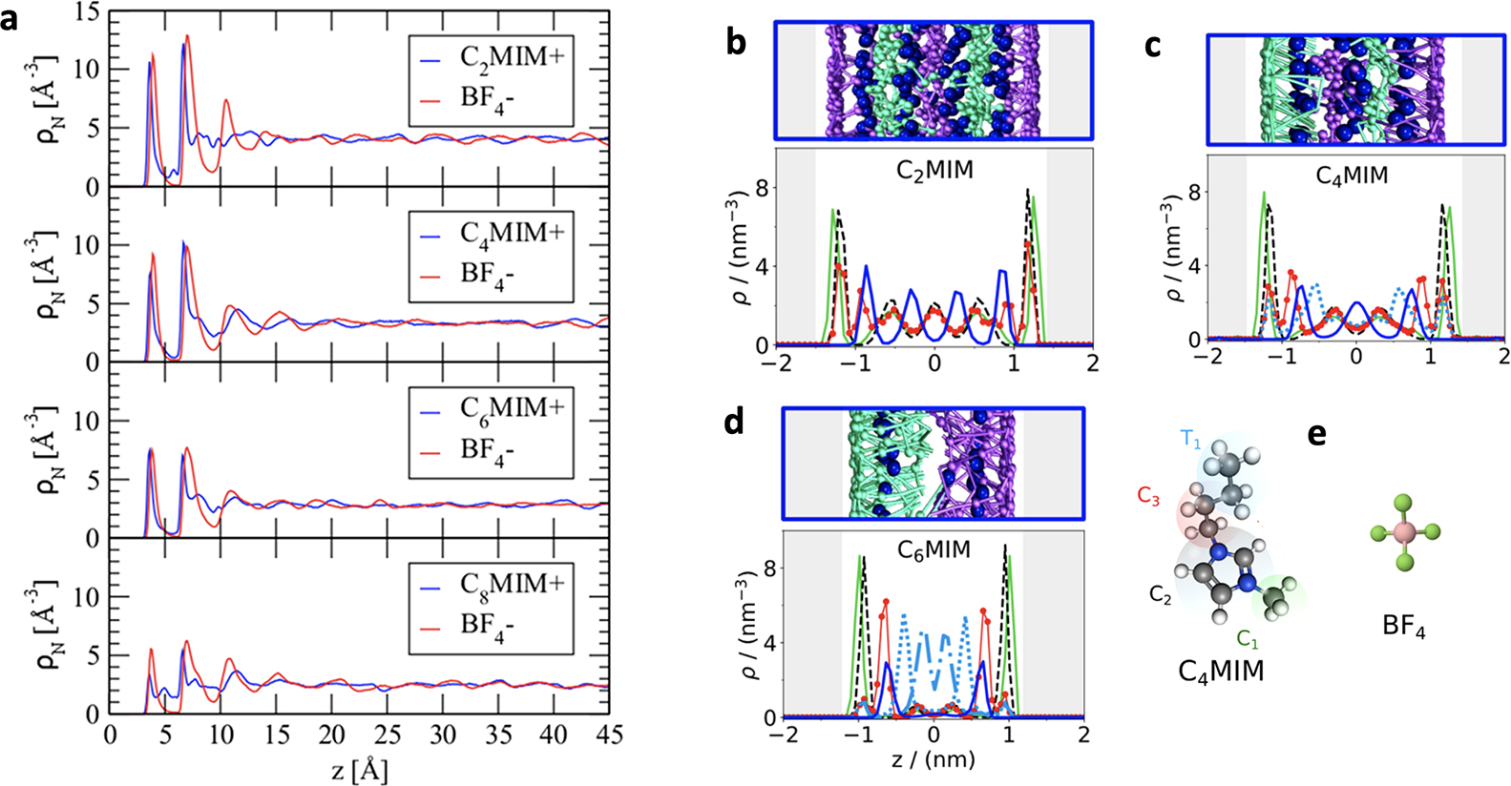
Structuring of RTILs at flat surfaces and inside slit pores. (a) Number density profile of the center of mass of [C_n_MIM][BF_4_] ionic liquids (full atomistic models) confined between two neutral graphene planes. Reproduced with permission from ref (319). Copyright 2019 American Chemical Society. (b–d) Density profiles of [C_n_MIM][BF_4_] ionic liquids (coarse grained models) confined between negatively charged surfaces (−32 μC cm^––2^). The snapshots illustrate the segregation of the aliphatic chain toward the interior of the films. The anions ([BF_4_]^–^) are represented as blue spheres, and the cations are colored according to the color scheme in (e). (b–e) Reproduced with permission from ref (233). Copyright 2020 American Chemical Society.

Nanoscale confinement between slit nanopores can induce a structuring of the ionic liquid in the direction normal to the wall. The structuring emerges from breaking the translational symmetry of the systems, resulting in the characteristic oscillations in the direction normal to the substrate with a wavelength corresponding roughly to the ion diameter^319^ (Figure 23a). The oscillations decay exponentially with the distance from the wall. For weak confinements (slit separations >10 nm) between neutral walls, the characteristic decay length is similar to that of the bulk radial distribution functions. Under stronger confinement (below 3 nm), there is a significant interference between the density oscillations emerging from both pore walls. The interference leads to structural forces (see below).

The charging of the surface triggers important changes in the structure and dynamics of confined ionic liquids. Charged surfaces induce charge oscillations (Figure 23b) with overscreening settling in as soon as the substrates are slightly charged. At high surface charges, typically above |σ|∼ 32 μC/cm^2^, e.g., for mica surfaces, the RTIL layer in contact with the surface might fully compensate the surface charge, depending on the substrate and ion compositions.^320^

Confinement induces an in-plane structure in RTILs too. This effect is of particular interest in friction (see discussion in section 6.4.7 and Figure 36). Crystallization under confinement at neutral and charged substrates has been observed in simulations of primitive, coarse-grained, and atomistic models, using in the latter case small cations ([EMIM]^+^ and [BMIM]^+^).^226^ When the ionic liquid coats the surface completely, the ions might arrange into crystalline hexagonal 2D structures.^210,227,266^ The crystallization of RTILs containing larger cations (e.g., imidazolium cations with long aliphatic chains) conveys a higher entropic cost, and these large ions do not form crystalline structures, instead disordered non-polar and polar/charged domains have been reported.^319^

#### 6.2.2 Structural Forces in Slit Confinements

**Figure 24:**
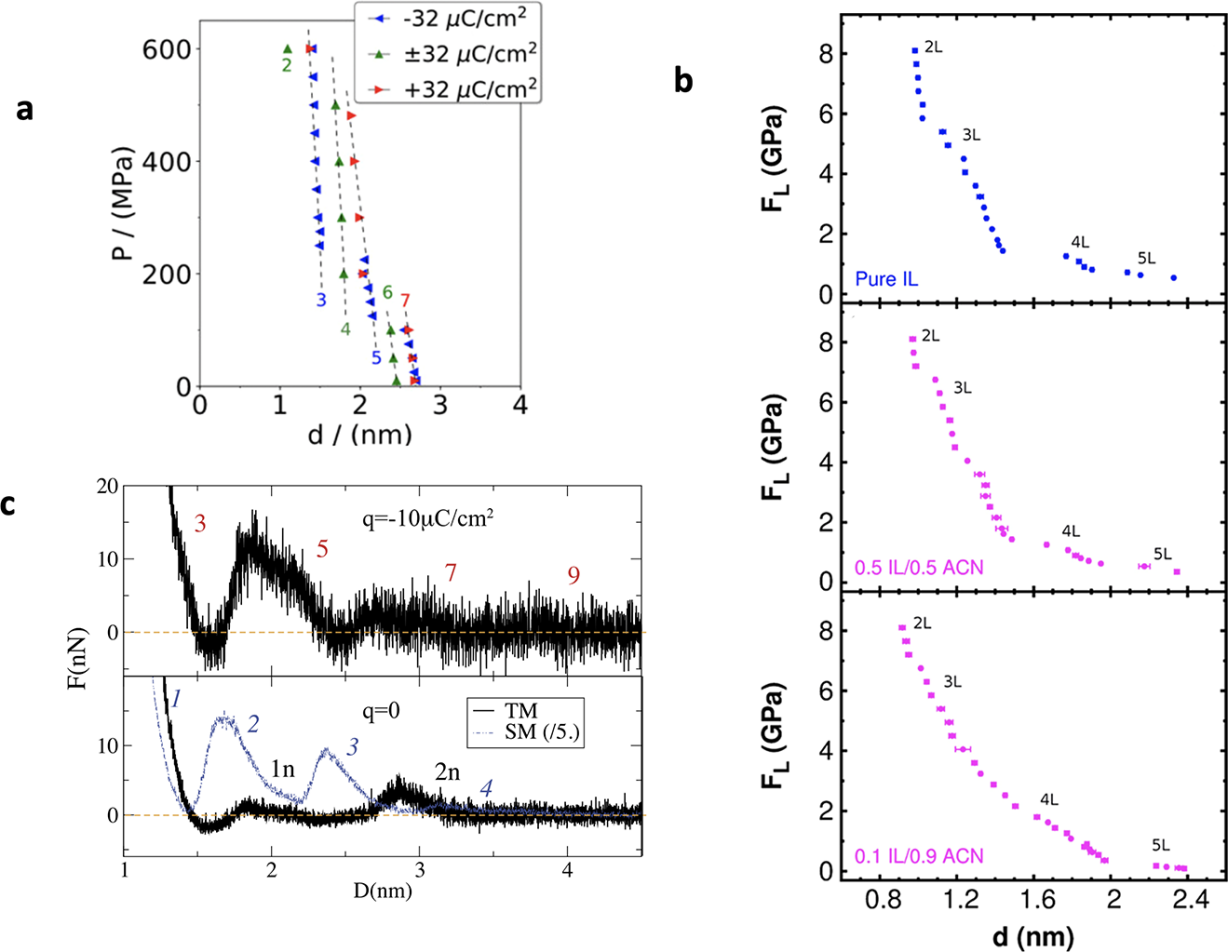
Structural forces obtained from GC-NEMD simulations. (a) CG models of imidazolium BF_4_ RTIL confined between substrates with positively or negatively charged substrates with surface charge density σ = ±32 μC/cm^2^. Reproduced with permission from ref (233). Copyright 2020 American Chemical Society. (b) Structural forces of pure CG RTILs and RTILs dissolved in acetonitrile (molar ratios 0.5:0.5 and 0.1:0.9) and confined between charged plates with charge density σ = −32 μC/cm^2^. Adapted with permission from ref (321). Copyright 2019 American Chemical Society. (c) Force–distance curves for coarse grained models of ionic liquids. (top panel) Ionic liquid confined between negatively charged surfaces with σ = −10 μC/cm^2^ on both surfaces. (bottom panel) Ionic liquid confined between neutral plates. SM and TM refer to models of spherical ions and coarse grained ions with tails, respectively. Reproduced with permission from ref (209). Copyright 2015 American Chemical Society.

GC-NEMD simulations provide a direct route to calculate structural forces (Figure 24) and to model the squeezing-out of ionic layers with increasing normal load, hence establishing a direct connection with SFA^323^ and AFM^324^ experiments. The surface charge in nanopores induces alternating charge layering in confined RTILs, with the RTIL layers in direct contact with the surface being enriched in cations (negatively charged surfaces) or anions (positively charged surfaces). The boundary conditions, imposed by the charged walls, result in the formation of an odd or even number of alternating charged layers for like and unlike charged surfaces, respectively. Hence, upon applying an external load, the thinning of the film proceeds usually by expelling two layers of opposite charge at a time for like charged pores, with an odd number of confined layers, 3, 5, 7, ... (Figure 24a (blue symbols) and Figure 24c (red labels)). When the surfaces have opposite charge, the nanopore contains an “even” number of confined layers (2, 4, 6, ...) (Figure 24a (green symbols and lines)).

The addition of organic solvents to RTILs modifies the layering in the confined nanofilms, and at high dilution of IL (below 10% molar fraction) and high electrode charges, the layered structure of the nanofilm is less pronounced.^321^ Correspondingly, these diluted RTILs (e.g., [BMIM][BF_4_] in acetonitrile) feature a different squeeze-out mechanism, which proceeds by ejecting a single layer at a time (see Figure 24b), highlighting the significant impact of solvents on the RTIL film structure under confinement. Importantly, simulations of imidazolium RTILs dissolved in acetonitrile demonstrated that the ions adsorb preferentially at the charged electrodes, even at 10% RTIL content.^321^

#### 6.2.3 Ionic Liquids in Cylindrical Nanopores

The ability to synthesize carbon and inorganic nanotubes with a wide range of diameters has motivated the investigation of the structure of RTILs confined in cylindrical geometries.^326−328^ In this context, carbon nanotubes (CNTs) have been frequently used in molecular modeling. CNTs are obtained by wrapping a graphene strip and characterized by a pair of indices (*n*, *m*), where *n* and *m* are two integers representing the number of unit vectors along two directions in the honeycomb lattice of graphene, defining the graphene strip.^329^ The diameter of an (*n*, *m*) CNT is , where *a* = 0.142 nm is the carbon–carbon bond length.^330^ Typically, an armchair (*n*, *n*) CNT is metallic, an (*n*, *m*) CNT with *n* – *m* multiple of three is quasi-metallic, and all other CNTs are semiconducting;^330^ we note, however, that there are exceptions.^330−332^

**Figure 25:**
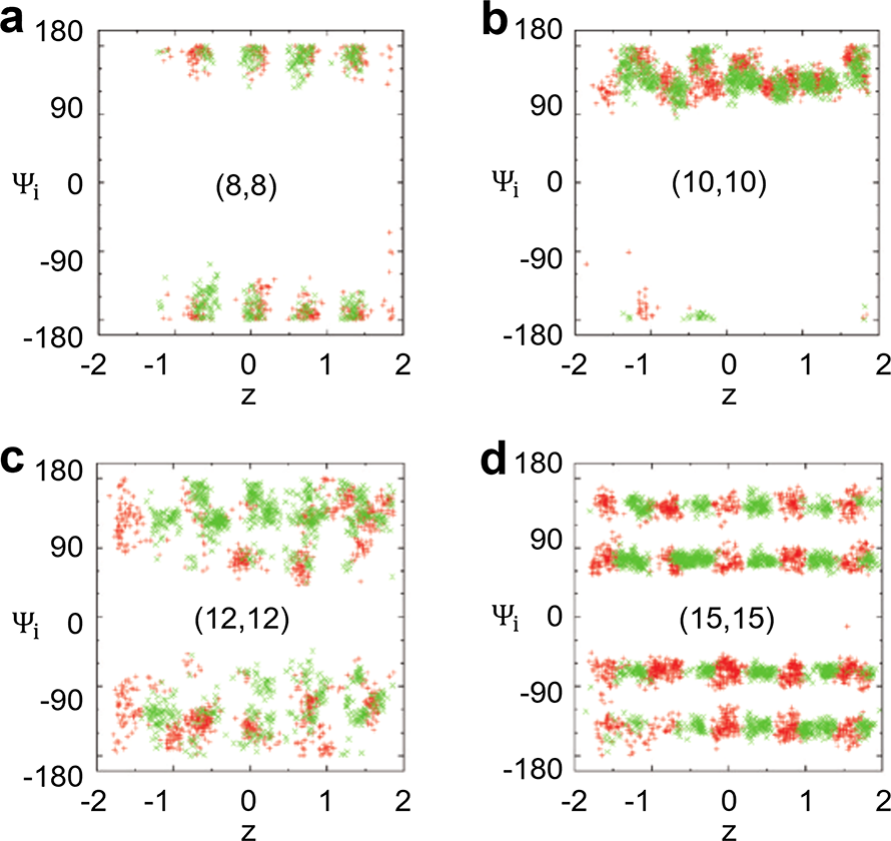
Structure of ionic liquids confined in carbon nanotubes. (a–d) Rotational angle Ψ_*i*_ between two neighboring ions in the first internal solvation shell of nanotubes. Red and green markers represent cation and anion, respective. Reproduced with permission from ref (325). Copyright 2009 American Chemical Society.

Shim and Kim^325^ investigated the solvation structure of [EMIM][BF_4_] confined in neutral single-walled carbon nanotubes (SW-CNTs) in armchair (*n*, *n*) configuration with MD simulations. These authors demonstrated that a smeared-out cylindrical shell-like structure was formed outside of SW-CNTs with a primary and a secondary peak located around 0.35 and 0.8 nm from the exterior nanotube surface.^325^ Ascribed to the π-stacking, the imidazole ring of cations in the first external solvation shell tends to be parallel to the SW-CNT surface, allowing bulky cations to approach the nanotube surface more closely than corresponding anions in the first solvation shell; nevertheless, the arrangement of the ring was essentially isotropic relative to the radial direction.^325^ A slight modulation of the nanotube diameter could cause a significant change in the solvation organization inside the SW-CNTs. Shim and Kim^325^ evaluated the solvation structure quantitatively by calculating the rotational angle (Ψ) between two neighboring ions (Ψ_*i*_ = ϕ_*i*+1_ – ϕ_*i*_, where *i* labels the ion inside nanotubes, and ϕ_*i*_ is the angle between the ring normal and the radial vector from the nanotube axis). A distribution without the sign change of rotation angle corresponds to a chiral structure, and a distribution with the rotational angle at around ±180° indicates a zigzag structure. As depicted in Figure 25, the arrangement of cation and anion inside (*n*, *n*) SW-CNTs varied from zigzag distribution with ion pairing (*n* = 8), to chiral distributions (*n* = 10) and to more disordered configurations for *n* ≥ 12.^325^

### 6.3 Capacitance and Energy Storage

#### 6.3.1 Slit Nanopores

**Figure 26:**
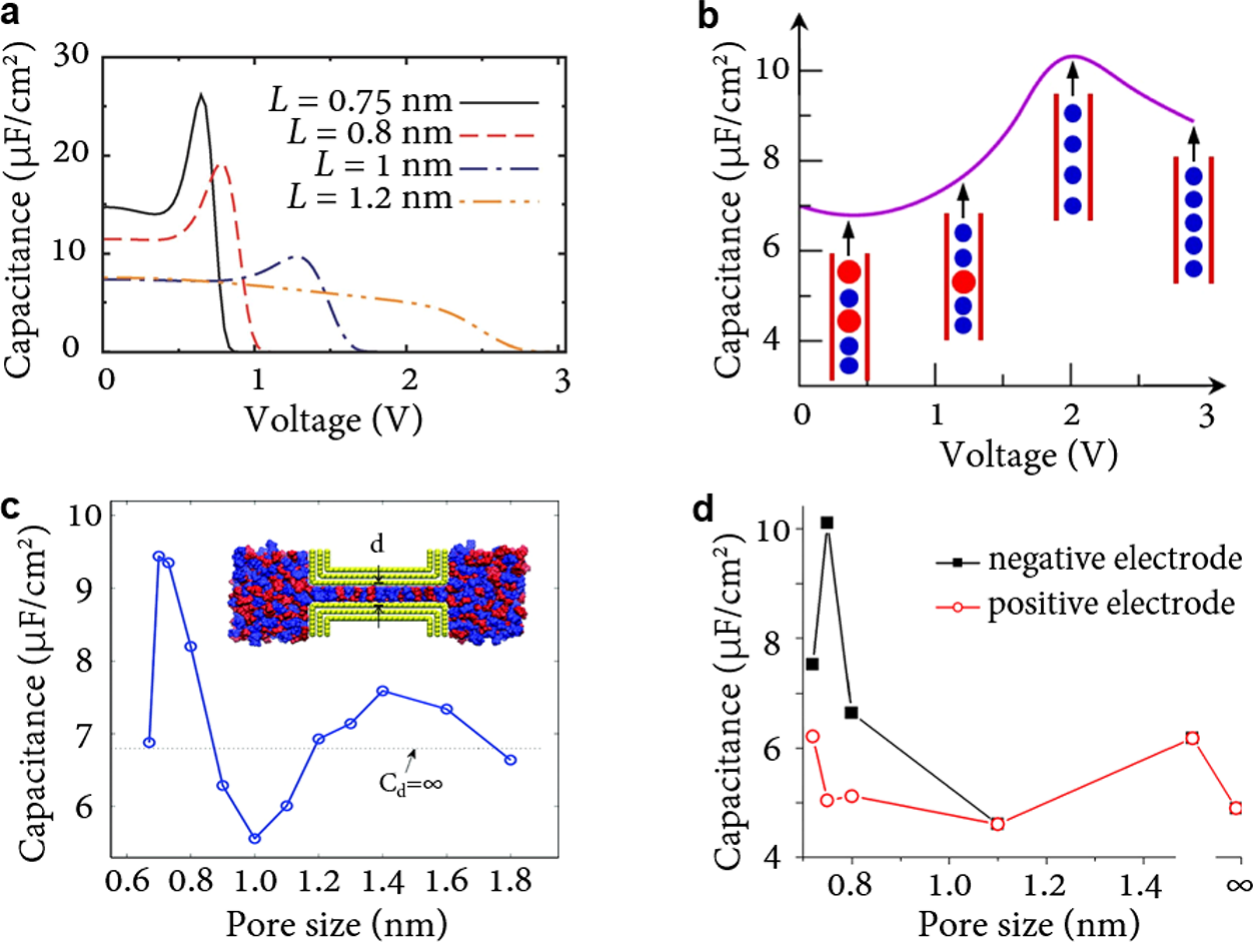
Capacitance of ionic liquids in slit nanopores. (a) Differential capacitance in nanopores as a function of the electrode potential for restrictive primitive model of ionic liquids. Reproduced with permission from ref (49). Copyright 2011 Royal Society of Chemistry. (b) Integral capacitance as a function of electrode potential for [DMIM][BF_4_] ionic liquid. Blue and red circles represent counterions and co-ions. Reproduced with permission from ref (333). Copyright 2012 American Chemical Society. (c) Integral capacitance in nanopores as a function of pore size for [EMIM][TFSI] ionic liquid. Reproduced from ref (182). Copyright 2011 American Chemical Society. (d) Capacitance of negative and positive electrodes as a function of pore size. Reproduced from ref (220). Copyright 2013 American Chemical Society.

Slit-shaped pores have been often used in molecular simulations as the simplest models for nanoporous electrodes. For instance, Monte Carlo simulations of the restrictive primitive model (charged hard spheres) of an ionic liquid showed how the superionic state leads to the anomalous increase of capacitance in nanopores^49^ (see section 3 and Figure 5). The authors of ref (49) also studied the voltage dependence of differential capacitance. They found that it is almost constant at low voltages and exhibits a peak before vanishing at large voltages when the pore saturates with counterions (Figure 26a).

Wu et al.^181^ considered a more complex ion model corresponding to [DMIM][BF_4_] ionic liquid. They showed that the integral capacitance depends on the electrode potential and related this behavior to charging mechanisms (Figure 26b). At low voltages, the charging was dominated by swapping the in-pore co-ions for counterions from the bulk electrolyte; as the voltage increased, the charging became driven by co-ion desorption and the capacitance increased as a function of the electrode potential (see also ref (71)). The capacitance reached its maximum when all co-ions were desorbed from the pore. For yet higher voltages, the charge storage became dominated by counterion adsorption and the capacitance reduced due to entropic effects.

Wu et al. also observed a damped oscillatory behavior of the (integral) capacitance with the pore size. A similar oscillatory behavior was revealed by Feng and Cummings^182^ in the case of [EMIM][TFSI]. The capacitance increased with decreasing the pore size from 1 to 0.7 nm, in line with the anomalous capacitance increase^18−20^ (sections 4.2.1 and 5.2.1); the second peak was found for pore widths between 1 and 1.8 nm (Figure 26c). The authors explained the oscillatory behavior by the interference of two double layers originating from the two walls of a slit nanopore. We note that the damped oscillatory behavior was also found with classical DFT (section 5.2.1 and Figure 11).

Xing et al.^220^ showed that the capacitance of [EMIM][TFSI] in subnanometer pores could be asymmetric in positive and negative electrodes (Figure 26d). For 0.75 nm pores, the negative electrode delivered about two times higher differential (and integral) capacitance than the positive one, with the latter being comparable to the capacitance of a planar electrode. This asymmetry in the capacitance was attributed to different atomic structures and charge distribution of [TFSI]^–^ and [EMIM]^+^, which caused a [TFSI]^–^ to interact stronger with the pore walls compared to [EMIM]^+^, leading to different pore fillings and capacitances in the two electrodes.

#### 6.3.2 Nanoslits in Studies of Capacitance and Energy Optimization

**Figure 27:**
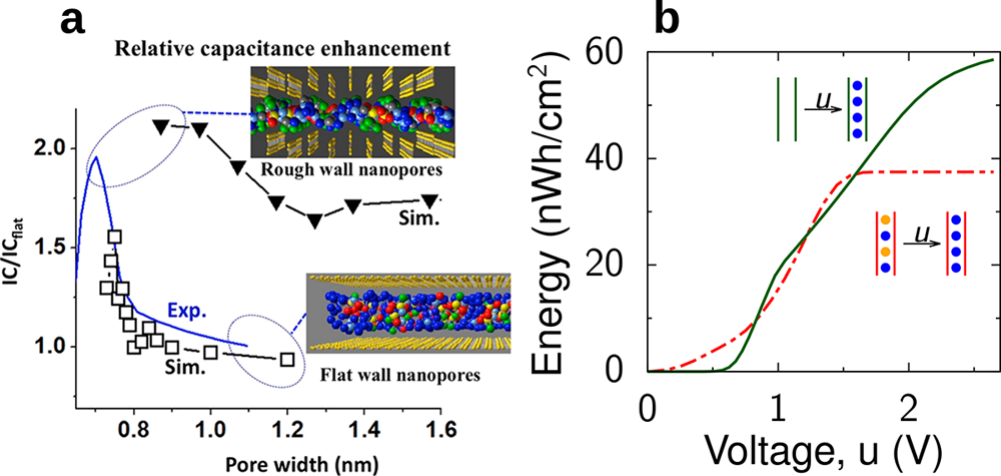
How to enhance capacitance and energy storage. (a) Pore wall roughness can increase the integral capacitance. The plot shows the results of MD simulations for slit-shaped pores and [C_2_MIM][TFSI] ionic liquid. Reproduced with permission from ref (80). Copyright 2015 American Chemical Society. (b) Ionophobic pores can store higher energy at large applied potential differences compared to conventional, ionophilic pores. The plot shows the results of Grand Canonical MC simulations for restrictive primitive model of ionic liquids confined in slit nanopores. Blue and red circles represent counterions and co-ions. Reproduced with permission from ref (71). Copyright 2016 Royal Society of Chemistry.

Slit pore models have also been used to investigate possible routes to enhance the capacitance and energy storage of nanoporous supercapacitors.^71,80,335−338^ For instance, Vatamanu et al.^80,338^ showed that surface roughness could increase the integral capacitance even two times compared to smooth pore walls (Figure 27a). They argued that rough edges of nanopore walls could increase the spatial separation between the co and counterions inside the nanopores at low and intermediate voltages. Interestingly, at large applied potential differences, rough pore walls provided higher counterion densities and boosted the integral capacitance.^80^

MC simulations of the restrictive primitive model of ILs showed that ionophobic pores could provide a higher energy density than conventional ionophilic pores^71^ (Figure 27b, see also Figure 3). As explained in section 4.1.2, the reason is that the pore ionophobicity shifts the charging to higher voltages, leading to higher stored energy densities according to eq 4. This conclusion was later confirmed by classical DFT^183^ (section 5.2.2) and MD simulations.^318,339^

By averaging the MC simulation results for slit nanopores over different distributions of pore sizes, it was shown that monodisperse nanoporous electrodes could maximize the energy storage for both ionophilic^70^ and ionophobic pores.^71^ In more recent work, ref (287) used a similar approach to analyze the connectivity of pores for typical carbons and found that well-percolated porous electrodes could substantially increase the stored energy density. While it remains to be seen how to fabricate such ideally percolated monodisperse electrodes, these theoretical results emphasize the importance of good quality nanoporous electrodes for enhancing energy storage.

#### 6.3.3 Cylindrical Pores and Carbon Nanotubes

Shim and Kim^340^ have been probably the first who performed MD simulations of charging carbon nanotubes (CNTs). They used [EMIM][BF_4_] ionic liquid and found an “anomalous” increase of the integral capacitance as the pore size was decreased from 2 to 0.9 nm. However, the capacitance was about 1 order of magnitude smaller than the experimental values. While the reason for this discrepancy is unknown, we note that these authors used constant charge simulations and analyzed the integral rather than the differential capacitance, which could contribute to the discrepancy.

Vatamanu et al.^80^ investigated the capacitance of [EMIM][TFSI] and [Pyr13][FSI] in CNTs. They found that the capacitance of [Pyr13][FSI] barely showed any enhancement in subnanopores, while the capacitance of [EMIM][TFSI] increased similarly as in the experiments of ref (20). The discrepancy in capacitance change between [EMIM][TFSI] and [Pyr13][TFSI] is owed to the chemical structure of ILs, which determines what type of transition occurs inside the nanopore. Specifically, RTILs containing [C_n_MIM]^+^cations exhibited an abrupt expulsion of co-ions from the nanopore, resulting in sharp changes in ion composition and densities inside the pore with increasing voltage; whereas in [Pyr13][TFSI], the charge separation inside nanopores was occurring via monotonic swapping ions and smooth change in composition with increasing voltage.

Pak and Hwang^341^ studied the relationship between the capacitance and charging dynamics for [EMIM][BF_4_] in CNTs open from both ends. They observed an electroneutral ionic liquid region forming in the middle of the pore, which they claimed to be detrimental to the integral capacitance. We note, however, that these authors observed nonequilibrium structures. Indeed, they found that the length of the electroneutral region depended on the voltage scan rate and decreased with decreasing the scan rate (section 6.4.4).

More recently, McDaniel^342^ modeled a supercapacitor consisting of CNT/graphene composite electrodes with [EMIM][BF_4_]/acetonitrile electrolyte. They focused on the topology of electrode–capacitance relationship and found that the intersection points where the CNTs and graphene conductors meet could serve as “hot spots” with a locally enhanced propensity for counterion adsorption, which led to the increased capacitance compared with isolated CNT or graphene surfaces. Such enhancement is ascribed to the strong electrostatic interactions with induced charges near the intersection of the CNT and graphene conductors, leading to an increasing number density of anions at larger positive voltage.^342^

Ma et al.^343^ investigated the charge storage of [EMIM][TFSI] and its mixtures with solvents in CNTs and compared it with slit pores. They observed that the integral capacitance (*C*_I_) oscillated witht the pore size more strongly in CNTs,^343^ likely due to the stronger confinement. They also found that *C*_I_ was not significantly altered by adding solvent. This conclusion is in line with an earlier work by Burt et al.^298^ on nanoporous carbons.

A few authors performed Monte Carlo simulations of the restricted primitive models of ILs in cylindrical nanopores.^44,60,62^ However, these simulations have been mainly used to validate analytical models. We, therefore, skip this discussion here and refer the readers to section 4.1.

#### 6.3.4 Nanoporous Carbons

Nanoporous carbons consist of interconnected micro- and mesopores, often of different sizes, shapes, and topologies. Examples include carbide-derived carbon (CDC), zeolite templated carbons (ZTC), and ordered mesoporous carbons, also called carbons mesostructured by KAIST (CMK) to mark the contribution from KAIST, a Korean company specializing in mesoporous carbons. Atomistic simulations of ILs under such complex confinements have become essential in energy research.^51,123,261,298,344−347^ In a computer, nanoporous carbons can be generated via MD simulations mimicking the synthesis process^348−350^ or with Gaussian random fields,^351,352^ a method introduced in late 1980 to generate bicontinuous phases and porous structures.^353,354^

**Figure 28:**
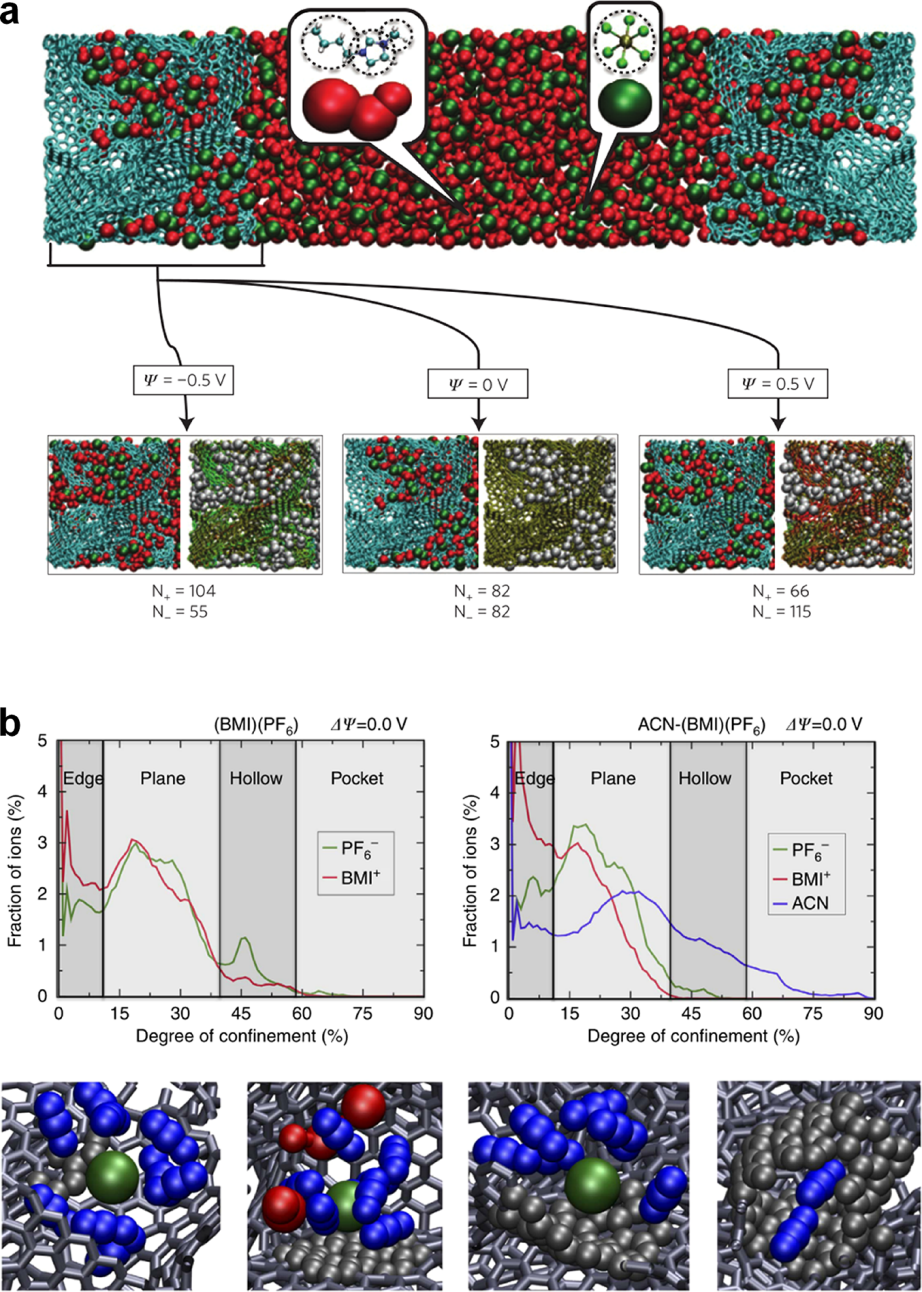
Ionic liquids under complex confinement. (a) The simulation cell consists of a [BMIM][PF_6_] ionic liquid and two porous electrodes (CDC-1200) held at a constant electrical potential.^261^ Reproduced with permission from ref (261). Copyright 2012 Nature Publishing Group. (b) Adsorption of ions in a nanoporous carbon electrode at zero potential difference, and the representative configurations of ions for the four adsorption modes: edge, plane, hollow, and pocket. The gray rods and gray spheres are the C–C bonds, and carbon atoms, respectively. The red color shows [BMIM]^+^ cations, the green colors denote [PF_6_]^–^ anions, and the blue color shows ACN molecules, respectively.^51^ Reproduced with permission from ref (51). Copyright 2013 Nature Publishing Group.

Merlet et al.^261^ have probably been the first who analyzed charging in realistically modeled CDC electrodes (Figure 28a). They used [BMIM][PF_6_] ionic liquid and found that the ions wetted the electrodes at zero voltage. In line with experiments,^18−20^ they observed an enhanced capacitance compared to flat electrodes and related it to the screening of electrostatic interactions by the conducting pore walls (i.e., the superionic state,^23^ see section 3) and the absence of overscreening in the electrolyte,^261^ resulting in better efficiency of charge storage inside microporous electrodes.^51^ Interestingly, two carbon materials (CDC-950 and CDC-1200) with similar pore size distributions provided integral capacitances that differed by about 43%. This difference indicates that fine details of the local structure (e.g., small graphitic domains) can significantly affect the capacitance.^261^

##### 6.3.4.1 Local Ion Environment

To describe the local environment experienced by confined ions in porous carbons, Merlet et al.^51^ introduced a degree of confinement (DoC) as the percentage of the solid angle around the ion occupied by carbon atoms and normalized by the maximal value taken by this quantity^51^
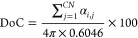
72Here the factor 0.6046 results from the fact that only about 64.46% of the closed surface is effectively covered by carbon atoms,^51^*CN* is the coordination number of carbons around ion *i* and α_*i*,*j*_ is the solid angle associated with carbon atom *j*, located at a distance *d*_*i*,*j*_ from this ion^51,355^
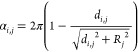
73where *R*_*j*_ is the radius of the region occupied by carbon atom *j*. Figure 28b shows several typical types of sites with the DoC ranging from 0% to 90%. These sites include (1) an edge site with a concave curvature, (2) a plane site with a local structure of a graphene sheet, (3) a hollow site with a convex curvature, and (4) a pocket where the ions are inside a subnanometer carbon pore with a cylinder-like shape. Merlet et al.^51^ found that the ions become less solvated with the increase of confinement, in agreement with experimental studies.^356,357^ These authors also showed that the local charge stored at the surface of the electrode increases as the DoC of the adsorbed ions increases.^51^

Liu et al.^358^ investigated the relationship between the geometric descriptors and average local interfacial properties for ZTCs. They found only a weak correlation between the capacitance and the commonly used geometric descriptors, such as density, void fraction, and averaged local interfacial properties, including the average distance between counterions and electrode and DoC. However, these authors introduced a new parameter, “charge compensation per carbon” (CCpC), and found that it is more strongly correlated with the capacitance.^358^ A CCpC characterizes how much of the ionic charge is compensated per electrode atom in the coordination shell. They showed that a high CCpC is associated with efficient charge storage because high partial charges of the electrode could screen the counterion charge, enabling higher ion loading and hence charge within the electrode at a fixed applied voltage. Adsorption sites with a high CCpC tended to be within pockets (cylindrical-like pores^261^) with a small radius of curvature, where the counterions could minimize their distance to the electrode surface and therefore induce stronger local partial charges.

##### 6.3.4.2 Pore Shapes

To describe the relationship between the capacitance and the electrode topology, Pak and Hwang^359^ introduced a pore shape factor (PSF) unifying the commonly used characteristics, viz., average pore width (*W*_p_), pore volume (*V*_p_), and specific surface area (*S*)
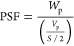
74According to eq 74, slit-shape pores have PSF = 2, cylindrical pores PSF = 4, and spherical ones PSF = 6. By comparing the areal capacitance as a function of PSF from different experimental sources, Pak and Hwang^359^ found that the maximum areal capacitance of different pores appeared at PSF ≈ 3.7, close to cylindrical pores. This result is consistent with the conclusion by Merlet et al.,^51^ showing that highly confined ions store charge more efficiently.

It has been suggested^70^ that the capacitance of a complex nanoporous electrode could be predicted by averaging the capacitance of single (isolated) pores over the distribution of electrode’s pore sizes. In a recent work, Mo et al.^122^ demonstrated such an “equivalent capacitance” principle for an electrode consisting of two slit pores of (generally) different widths. Of course, the averaging over pore sizes works in this case (and generally for a set of disconnected slit-shaped pores of different sizes). In a yet more general case, when pores are of different shapes, the pore shapes should also be taken into account, which has not been done so far, to our knowledge.

#### 6.3.5 Nanostructured Electrodes

Amorphous microporous carbons such as CDCs (section 6.3.4) have neither a regular structure with long-range order nor regular interpore connectivity, making them challenging to make explicit connections between the structure and performance. These drawbacks are avoided with zeolite templated carbons (ZTCs) and metal–organic frameworks (MOFs), which have volume-filling ordered structures and can provide large specific surface areas and custom-designed pore space. They are thus promising materials for EDLC electrodes.^360^ Nevertheless, studies of IL-filled MOFs and ZTCs are still scarce.

Liu et al.^358^ studied how regular pore geometry of ZTCs affects the charging dynamics and capacitance. They identified subtle differences in the local structure of ZTCs that led to large differences in capacitance. For instance, they found that the capacitance is higher in pores with sharp angles or low radius of curvature. Based on their simulations, they suggested using the same salt/ionic liquid as in the electrolyte when templating a ZTC to tailor it for a given electrolyte.^358^

Méndez-Morales et al.^301^ compared the performance of CDC electrodes with a series of perforated graphene electrodes with single pore sizes. In contrast to ref (70), they found that CDCs performed better than monodispersed electrodes. The ions reorganized themselves inside the CDC, allowing the co-ions to move to larger pores, reducing their negative effect on the charge storage. We note, however, that Méndez-Morales et al.^301^ considered integral capacitance (i.e., the accumulated charge). The situation might be different for differential capacitance and stored energy density.

In a recent work, Bi et al.^124^ performed MD simulations of [EMIM][BF_4_] ionic liquid in conductive MOFs to study ion structure and capacitive energy storage. They observed a transformation from a camel-shaped (two peaks) to a bell-shaped (one peak) capacitance as a function of voltage when the pore size increased from 0.81 to 2.39 nm and related this behavior to the voltage-dependent ion distribution inside the pores. They found that the energy-power density performance of MOFs compares favorably with most reported carbon-based supercapacitors and obtained a very higher power density.

### 6.4 Dynamics

#### 6.4.1 Ion Diffusion

Diffusion of confined ions is fundamentally important and often determines the rate of charging microporous electrodes. Nevertheless, studies of in-pore ion diffusion are still scarce and many questions remain unanswered, as we discuss below.

**Figure 29:**
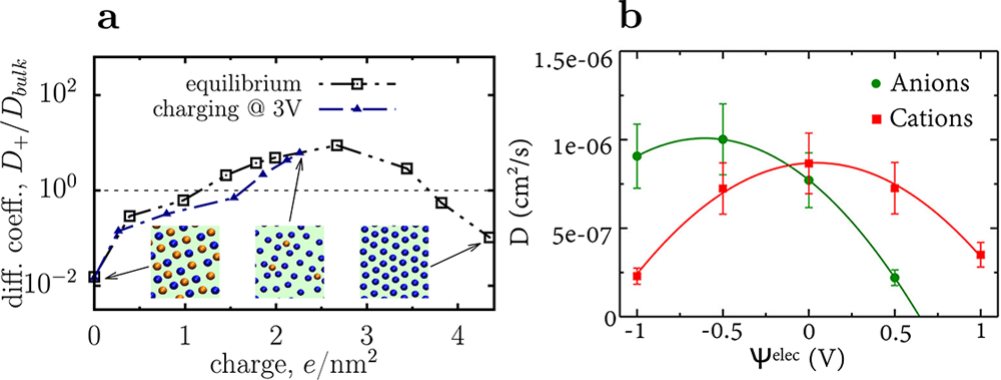
Diffusion of ions in nanopores. (a) Cation self-diffusion coefficient along the equilibrium path and along the path corresponding to the step-voltage charging. The diffusion coefficient is expressed in terms of the diffusion coefficient of a neutral bulk system (*D*_bulk_). Blue and orange spheres in the insets denote cations and anions. Reproduced with permission from ref (113). Copyright 2014 Springer Nature. (b) Ion self-diffusion in CDC electrodes (their snapshots can be seen in Figure 28a). Ψ is the voltage applied between two such electrodes. Reproduced with permission from ref (123). Copyright 2015 American Chemical Society.

For size-symmetric ionic liquids in ultranarrow slit pores, MD simulations showed that the self-diffusion coefficients *D*_±_ of cations and anions varied nonmonotonically with the charge on the pore walls^113^ (Figure 29a). For a neutral pore (corresponding to zero voltage), *D*_±_ was nearly 2 orders of magnitude smaller than in the bulk electrolyte. Such a low diffusivity was due to the ions forming a nearly crystalline two-dimensional structure. Ion diffusion speeded up with increasing the accumulated charge because the adsorbed counterions broke this nearly crystalline ionic order. For a highly charged pore, ion diffusion slowed down again; under this condition, there were mainly counterions left in the pore, which formed a locally ordered hexagonal structure, slowing down the diffusion (the insets in Figure 29a). A similar behavior has been observed in equilibrium and during charging, for pores of different widths, and for different electrolytes.^113,361^

Pean et al.^123^ investigated the ion diffusion in CDC electrodes (Figure 28a)) and observed a noticeably different behavior. While the diffusion coefficients varied appreciable with the potential, as for slit pores, they exhibited a maximum at low potentials and decreased monotonically with increasing the voltage (Figure 29b). This behavior is likely due to a complex network of interconnected pores, but the precise reason is unclear.

We note that neither behavior has been reproduced in experiments. Forse et al.^362^ measured ion diffusion in activated carbons with pulse field gradient NMR. In contrast to MD simulations, they observed only small variations of the diffusion coefficients with the applied cell voltage. The time scale of NMR experiment is in the range of micro- to milliseconds, while MD simulations operate on nanoseconds. It means that NMR experiments probe large-scale nanoporous structure with ion diffusion, rather than the short-time diffusivity measured in simulations. However, we are not aware of any study relating these experimental and simulation results.

#### 6.4.2 Charging Slit Nanopores: Four Charging Regimes

**Figure 30:**
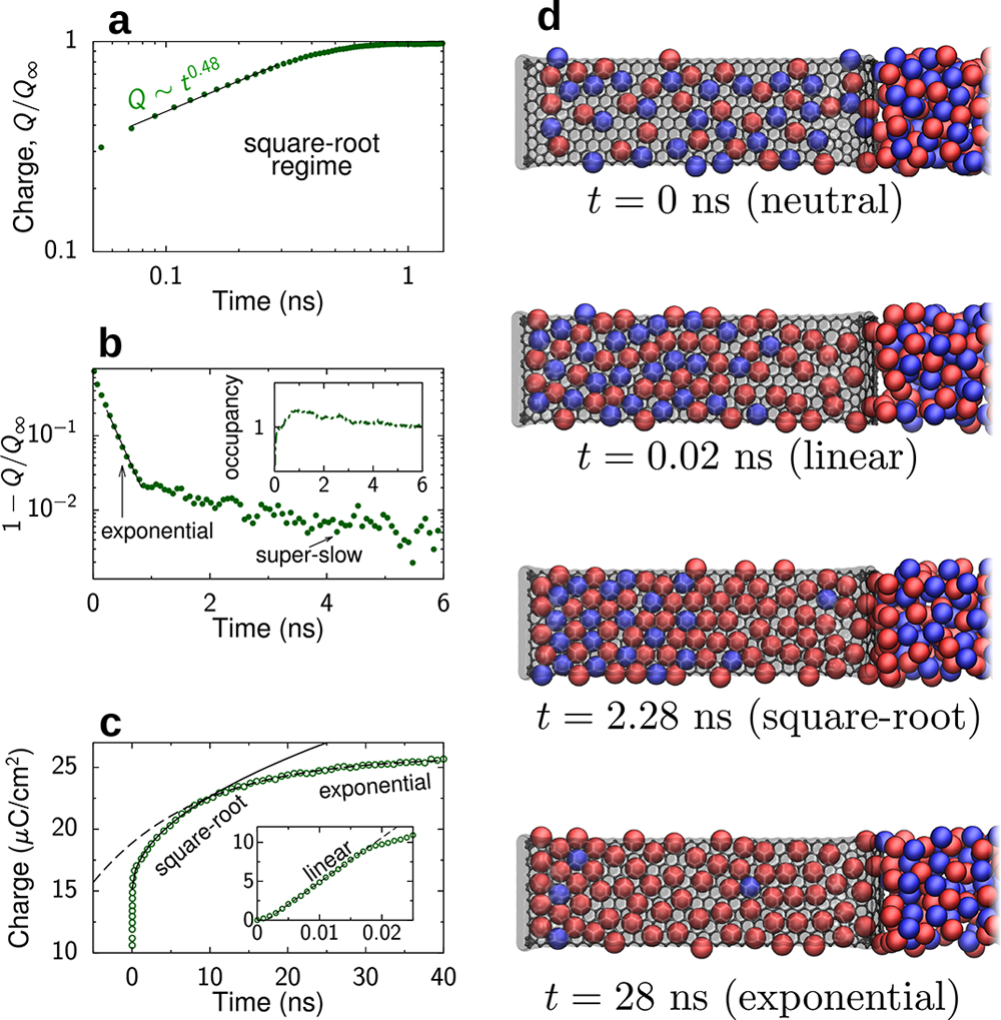
Four charging regimes from MD simulations. (a) At short times, the accumulated charge (*Q*) grows as a square root of time. (b) At later times, *Q* saturates exponentially and enters a superslow regime, during which it saturates exponentially with a larger decay time constant. The superslow regime is related to pore overfilling (the inset). Reproduced with permission from ref (113). Copyright 2014 Nature Publishing Group. (c) In addition to the square-root and exponential regimes, at very short times, there is a linear regime during which the charge grows linearly with time (see the inset). This regime is due to ion migration caused by the applied cell voltage acting on the ions outside of the pore. (d) Snapshots from MD simulations typical for various charging regimes. During the short-time linear regime, the neutral ionic liquids is “compressed” inside the pore by the adsorbed counterions. The charging proceed by melting an “interface” between a dense, neutral and a dilute, charged transient ionic liquid phases. Adapted with permission from ref (125). Copyright 2018 American Chemical Society.

As we discussed in section 4.5, analytical models predict that the charging of ultranarrow slit pores is diffusive with the accumulated charge growing as a square root of time at short times and saturating exponentially at late times (eqs 36 and 37). At yet later times, this model shows that the charging can enter a superslow regime, where the charge saturates exponentially but with a larger time constant (Figure 9). MD simulations confirmed the existence of all three charging regimes (Figure 30a,b).^113,125^ In addition, Breitsprecher et al.^125^ revealed a linear regime at the initial stage of charging (the inset in Figure 30c), which is not captured by the analytical theory of ref (112) and had been overlooked in earlier MD studies. It was, however, observed by Yang et al.,^126^ who used the modified Poisson–Nernst–Planck equations for wide cylindrical pores, although only when the bulk resistance was larger than that of the pore. The linear regime originates from ion migration due to the electrostatic forces acting on the ions outside of the pore, similarly to charging flat electrodes. For flat electrodes, this transient behavior depends on the Debye length and electrode separation.^363,364^ For nanopores, the characteristic time scale setting this regime depends on the pore length;^125^ MD simulations suggest that it might be related to how quickly the neutral ionic liquid inside the pores get “compressed” by the adsorbed counterions (Figure 30d). During this linear regime, narrow pores becomes overcrowded with ions, leading to sluggish dynamics.^42,125,341^

#### 6.4.3 Charging Complexly Nanostructured Electrodes

The fundamental understanding of the charging process with ionic liquids under complex nanoconfinement of realistic electrodes can help avoid compromising the power density when using such electrodes to enhance energy density. Nevertheless, only a few computational studies have reported the charging dynamics of complexly nanostructured electrodes. This scarcity is likely the result of the challenges in accounting for a constant potential applied at electrodes with complex nanostructures (sections 6.1.6 and 6.1.7).

**Figure 31:**
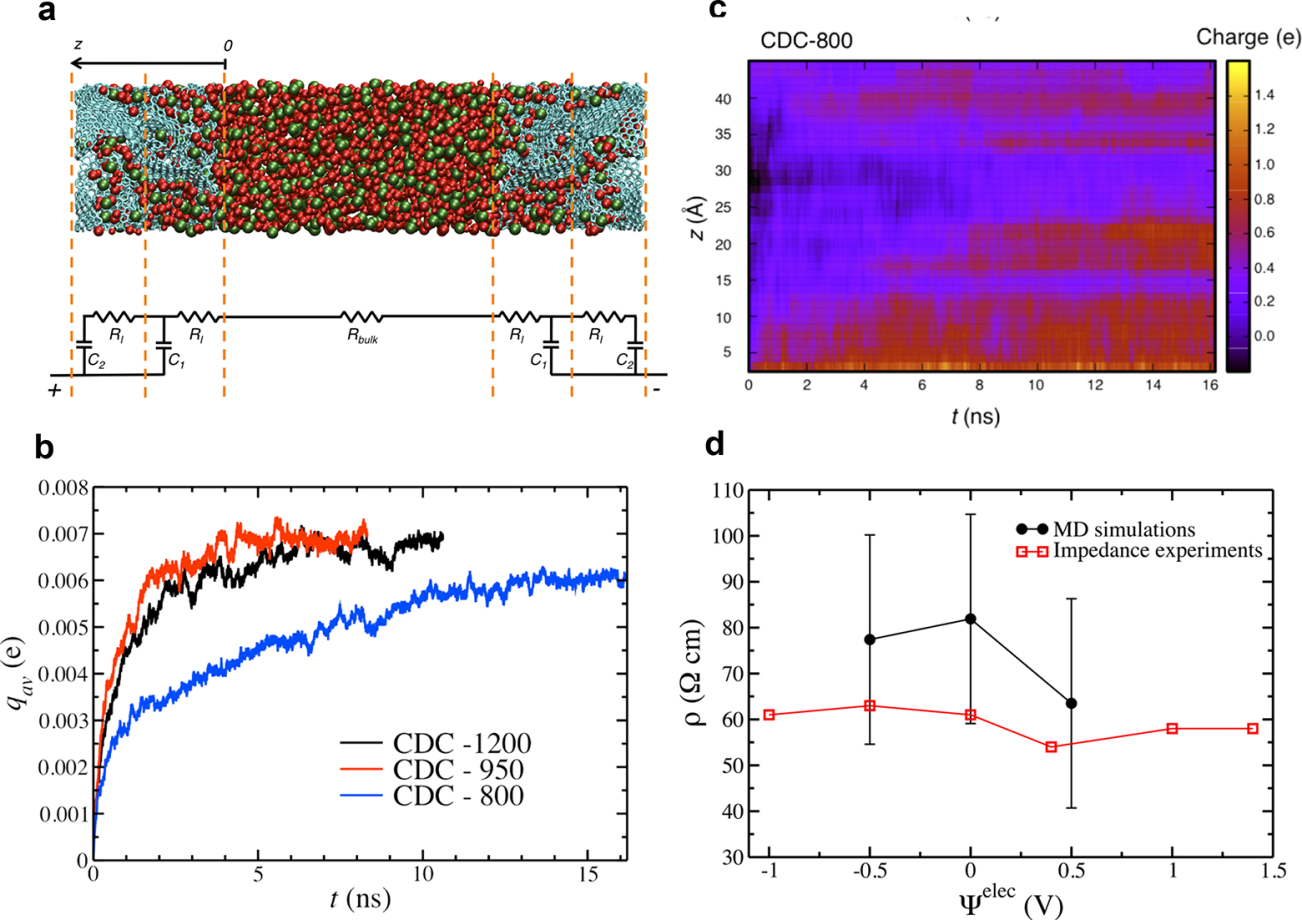
Dynamics of charging CDC electrodes. (a) Snapshot from MD simulations showing CDC-1200 (carbide chlorination temperature *T* = 1200 K). The gray sticks-and-balls show the nanoporous carbons while the red and green balls are the ions. The bottom schematic drawing shows an equivalent circuit used to interpret the simulation data. (b) Average charge per carbon atom as a function of time for three different CDCs. The average pore sizes are 9 Å for CDC-1200 and CDC-950 and 7.5 Å for CDC-800. (c) Evolution of the local charge with time as a function of the depth (*z*) inside the CDC electrodes, where *z* = 0 corresponds to the entrance of the porous electrode (see (a)). Reproduced with permission from ref (116). Copyright 2014 American Chemical Society. (d) Variation of the calculated and experimental in-pore resistivities with the electrode potential. Reproduced with permission from ref (123). Copyright 2015 American Chemical Society under CC-BY (http://creativecommons.org/licenses/by/4.0/).

Péan et al.^116^ performed MD simulations of three different CDC electrodes in [BMIM][PF_6_] (Figure 31a). They found that the CDC electrode characterized by the smallest average pore width *L*_*a*_ (viz., CDC-800 with *L*_*a*_ ≈ 7.5 Å versus *L*_*a*_ ≈ 9 Å of CDC-1200 and CDC-950) exhibited the slowest charging (Figure 31b). Given that narrower pores provide higher capacitances and stored energy densities (section 6.3), this result underlines the energy-power trade-off for nanoporous supercapacitors. These authors also observed “charging heterogeneity” at the nanoscale.^116^Figure 31c shows this heterogeneity in the form of the evolution of the charge with time as a function of the electrode depth; the charge was calculated on the carbon atoms for slabs of size 1 Å centered at *z*. The electrode at *z* ≈ 40 Å starts charging earlier than at *z* ≈ 35 Å and *z* ≈ 20 Å, which are closer to the pore entrance. This is because of a bulky pore ending (around *z* ≈ 25 Å) that acts as an electrolyte reservoir (the blue, practically uncharged region in Figure 31c). Thus, this charging heterogeneity originates from the structural heterogeneity of nanoporous electrodes.

The same group studied the effect of solvent and applied potential on the charging dynamics using the same CDC-based supercapacitors.^365^ They reported two charging regimes when the number of ions changed quickly and when it evolved more slowly. These two regimes likely correspond to the linear and square-root regimes (fast charging) and two exponential regimes (slow charging), reported for slit pores (section 6.4.2 and Figure 30). Péan et al.^365^ also found that the charging dynamics could be accelerated by applying a higher potential difference between the electrodes. It is probably because a higher potential could enhance counterion migration, speeding up the counterion adsorption. However, the quickly adsorbed counterions can overcrowd the pores and block the co-ions from diffusing out of the pore, as found for slit pores (Figure 30d). Why this did not happen for the studied CDCs is unclear.

Furthermore, Péan et al.^365^ identified the kinetic characteristics of the charging process. For a neat ionic liquid, they found that the charging mechanism was characterized by ion exchange, with counterion adsorption being faster than co-ion desorption. The rearrangement of counterions inside the electrode was also faster than co-ion rearrangement.^365^ Interestingly, these kinetic characteristics changed with the addition of solvent, so co-ion expulsion became faster than counterion adsorption.^365^ The rearrangement of counterions occurred mainly between planes and hollows, whereas the co-ions were reorganized primarily between the edges and planes. However, there was no preference for a certain type of site for acetonitrile.^365^ Burt et al.^298^ also observed that ion exchange dominated for CDC-800 immersed in pure RTIL with charging parameter (Section 2.2) *X* ≈ 0.1 and *X* ≈ 0.3 in the negative and positive electrodes, respectively. In a diluted organic electrolyte in CDC-800 (ACN mass fraction 67%), the charging mechanism switched to counterion adsorption. This transition may be attributed to the fact that counterions have easier access to the electrode surface, taking the place of solvent molecules that do not interact strongly with the surface or other adsorbed counterions.

Bi et al.^124^ studied charging dynamics of supercapacitors with electrodes based on metal–organic frameworks (MOFs) and [EMIM][BF_4_] ionic liquid. They considered three MOFs with pore sizes of 0.81, 1.57, and 2.39 nm. They found that the transmission line model (section 4.5.3) fitted the charging behaviors in all cases decently. The MOF with the smallest pore size (0.81 nm) exhibited the lowest relaxation time for the charging and the highest in-pore ion conductivity. This enhancement could be because this MOF had a size close to the half-integer multiple of the ion diameter (about 0.5 nm),^318^ but more work needs to be done to better understand this effect.

#### 6.4.4 Accelerating Charging

As discussed in section 6.4.2, the charging of narrow pores can be sluggish due to overcrowding and co-ion trapping,^42,125,341^ which occur during the short-time linear charging regime when the counterions from the bulk solution block a neutral ionic liquid inside the pores (Figure 30d). The question of practical interest that has been addressed in a few publications is, therefore, how to speed up the charging dynamics of ultranarrow pores. We recall that such pores enhance the capacitance and energy storage due to the anomalous capacitance increase^18,19^ (Figures 5 and 5) and superionic state^23,49^ (section 3).

**Figure 32:**
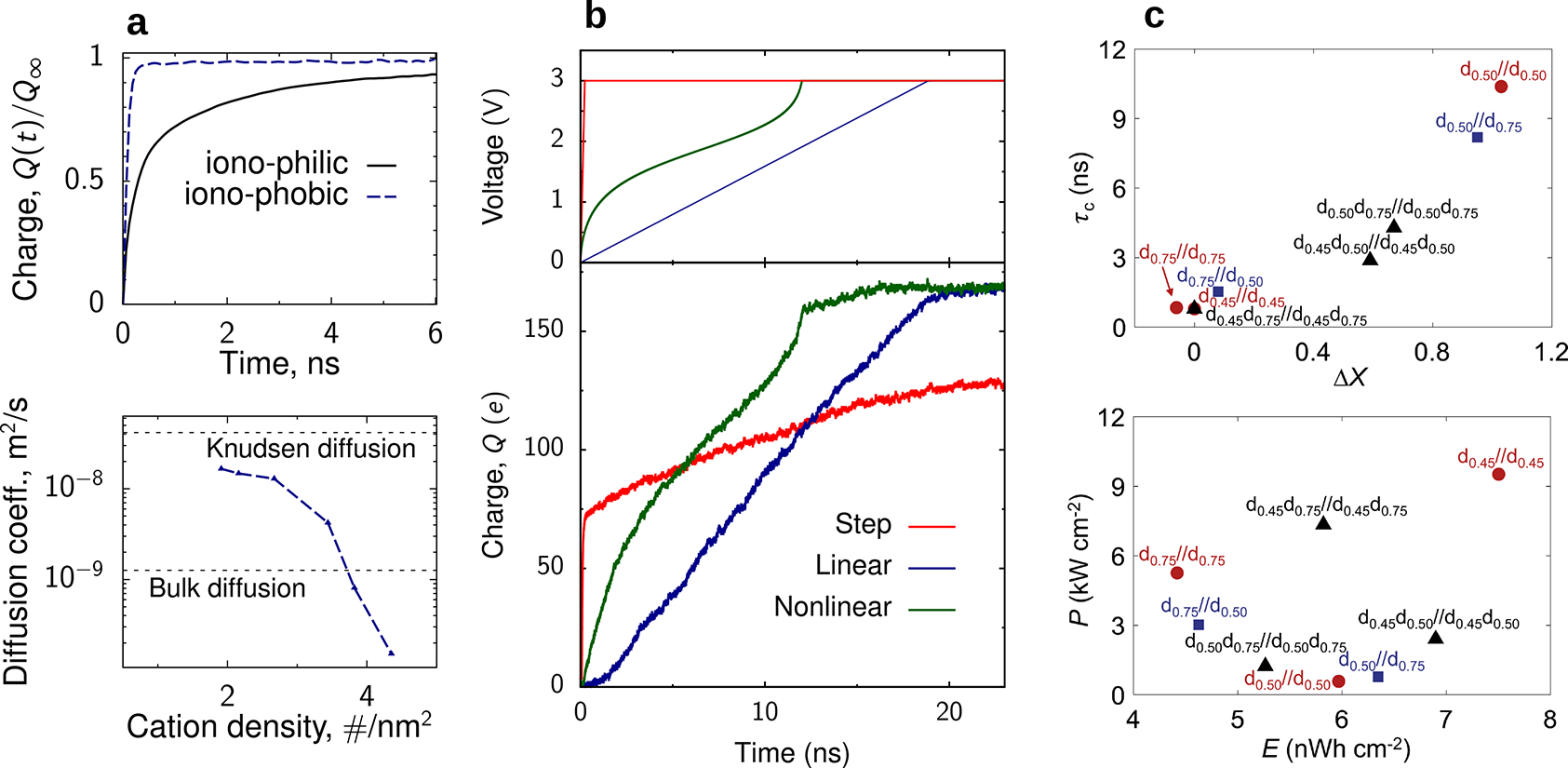
Accelerating charging dynamics. (a) Ionphobic pore can drastically speed up the charging dynamics, reducing the charging times by an order of magnitude. The bottom plot shows how the diffusion coefficient of a cation depends on the charge accumulated in the pore. Adapted with permission from ref^113^. Copyright 2014 Springer Nature. (b) Charging can be accelerated by applying the voltage slowly. The top plot shows the optimized linear and nonlinear charging protocols. The bottom plot compares the step-voltage charging with the optimal linear and nonlinear charging. Adapted with permission from ref (316). Copyright 2020 Nature Springer under CC-BY (http://creativecommons.org/licenses/by/4.0/). (c) Correlations between charging times and asymmetry in charging mechanism, described by parameter Δ*X*, eq 75 (Δ*X* = 0 means that charging mechanisms at two electrodes of a supercapacitor are identical; see the text). Each point in the plot corresponds to a supercapacitor consisting of two electrodes with one (//) or two (//) slit-shaped pores; *w*_1_ and *w*_2_ in this notation mean the pore widths. The bottom plot shows the Ragone plot, demonstrating that an ionophobic pore (d_0.45_//d_0.45_), free of ions at zero potential due to its small size, enhances both the power and energy density. Reproduced with permission from ref (122). Copyright 2022 Elsevier under CC-BY-NC-ND (http://creativecommons.org/licenses/by-nc-nd/4.0/).

One way of speeding up charging that has been theoretically explored in earlier work is making a pore ionophobic.^112,113^ Simulations of charging slit-shaped pores showed a dramatic decrease in the charging time of a pore that is empty at zero polarization (Figure 32a). Interestingly, this enhancement was much more significant than predicted by the analytical model of ref (112) (section 4.5.1). It is likely because this model assumed a constant (density-independent) ion diffusion, but MD simulations showed that it changes orders of magnitude during charging ionophobic pores (Figure 32a). Given the enhancement in energy storage at elevated (but experimentally achievable) voltages^60,71,76,183^ (Figure 3 and section 5.2.2), ionophobic pores offer an exciting opportunity to avoid the power-energy trade-off and boost both the power and energy storage. However, despite a few encouraging reports,^122,318,366,367^ it remains to be seen how to implement these ideas experimentally.

Another possibility to avoid the sluggish dynamics in narrow pores is to judicially control the rate with which the voltage is applied to a pore.^42,125,316,341^ Breitsprecher et al.^125^ found that there is a single optimal value of the linear sweeping rate *k*_opt_ that minimizes the charging time. They found , where  is the pore length (or the thickness of an electrode). In follow-up work, Breitsprecher et al.^316^ derived an expression for a nonlinear sweeping rate and demonstrated with MD simulations that it could provide even faster charging than the optimized linear sweep charging (Figure 32b). This enhancement has been achieved by adjusting the sweeping rate to the rate of co-ion desorption.^316^

Mo et al.^122^ took a different route and investigated how charging dynamics are related to charging mechanisms in a supercapacitor with (in general) asymmetric electrodes. They found correlations between charging times and asymmetry of charging mechanisms, characterized by a parameter
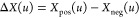
75where *X*_pos_ and *X*_neg_ are the charging mechanism parameters, eq 6, for positive and negative electrodes. Δ*X* = 0 means that the charging mechanisms are the same on both electrodes; a nonzero Δ*X* implies an asymmetry in the charging mechanism. Figure 32c shows that minimizing the asymmetry enhances the dynamics, providing shorter charging times. While the origin of these correlations is not well understood, they may have practical consequences for boosting the power and energy density of nanoporous supercapacitors. Figure 32c (bottom plot) shows the Ragone plot, demonstrating that a symmetric supercapacitor with 0.45 nm pore so narrow that it is free of ions at zero polarization, realizing an ionophobic pore) provides the highest energy and power densities. Interestingly, combining the ionophobic pore with wider pores within the same electrode could also enhance energy and power densities.^122^

#### 6.4.5 Accelerating Discharging

Discharging of narrow pores received much less attention, probably because discharging in capacitive energy storage depends on an external load, making it application-specific. However, discharging is essential in the capacitive deionization of saline water,^368−370^ enhancing the discharging dynamics can speed up water desalination, contributing to water security.

**Figure 33:**
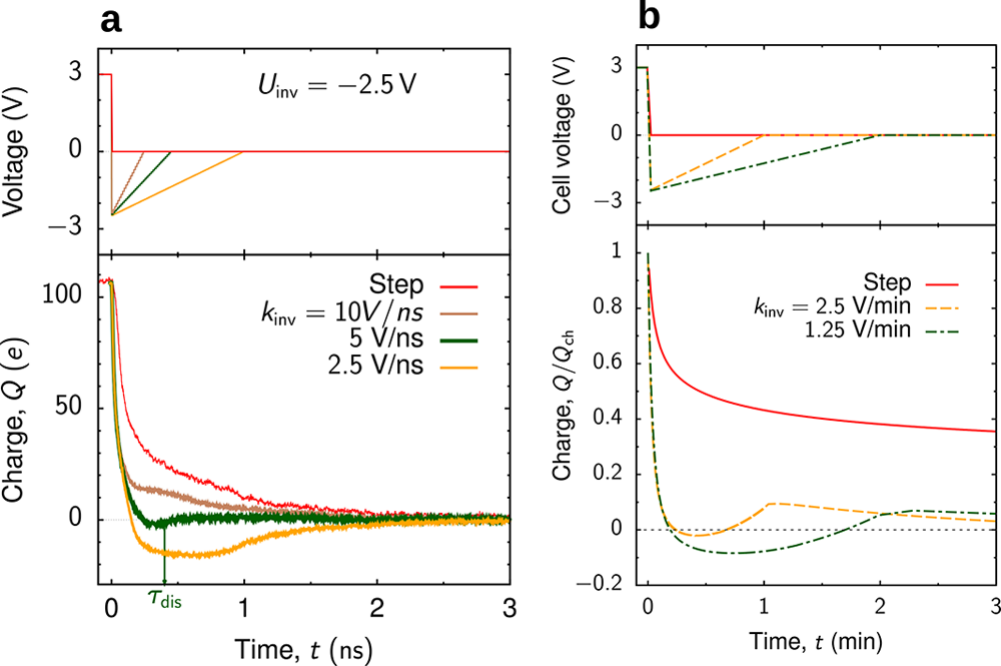
Accelerating discharging. (a) Discharging can be accelerated by applying voltage inversion, i.e., by dropping the voltage to *U*_inv_ and sweeping it to zero with rate *k*_inv_. The top plot shows examples of the voltage inversion protocol and the bottom plot compares their application with step-voltage discharging as obtained by MD simulations. (b) Voltage inversion in experiments with novolac-derived carbon and [EMIM]^+^-[BF_4_]^–^ ionic liquid as electrolyte. Adapted with permission from ref (316). Copyright 2020 Nature Springer under CC-BY (http://creativecommons.org/licenses/by/4.0/).

Breitsprecher et al.^316^ proposed a voltage-inversion protocol, showing with MD simulations and experiments that it could considerably speed up nanopore discharging. Instead of dropping the potential to zero, these authors considered a voltage drop *U*_inv_ to a potential opposite in sign to the charging potential and then varied the potential to zero with rate *k*_inv_ (Figure 33a); parameters *U*_inv_ and *k*_inv_ were taken as optimization variables to minimize the discharging time. With this discharging protocol, MD simulations demonstrated an approximately 4-fold decrease in the discharging time for a model supercapacitor with slit-shaped pores. Interestingly, experiments reported a much more significant reduction in the discharging times (Figure 33b).^316^ We note that this approach is relatively general and applicable to ionic liquids, inorganic electrolytes, and electrodes with virtually arbitrary porosity.

#### 6.4.6 Galvanostatic Charge–Discharge

In most theoretical and simulation studies on the charging dynamics, the charging process is controlled by voltage, that is, ion transport is driven by the potential difference applied between the electrodes. However, the charging dynamics driven by controlling the current is also common in practical applications and fundamental electrochemical studies.^18,371^

The charging dynamics in the current mode have been often studied by MD simulations using the constant charge method (CCM) .^372,373^ However, the importance of keeping electrodes equipotential in molecular modeling of electrochemical interfaces has been highlighted in a number of studies^48,48,116,312,318,374^ (see sections 6.1.6 and 6.1.7). While most constant potential methods (CPMs) have been developed to simulate dynamic processes in voltage mode, very few studies have been focused on modeling the charging and discharging process in the current mode with keeping the electrode equipotential.^375,376^

**Figure 34:**
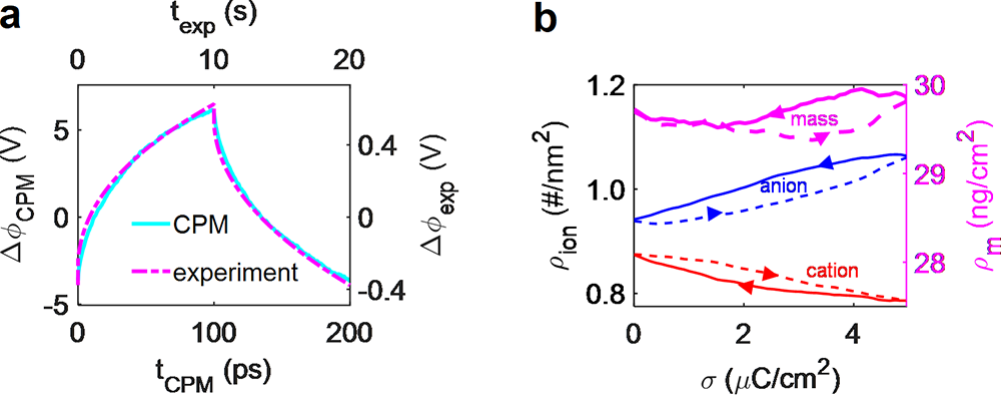
Galvanostatic charge–discharge of nanoporous electrodes with ionic liquid. (a) Galvanostatic charge–discharge curve from the experiment and CPM-based molecular simulation and the results of fitting by the diffusion equation. Δϕ_CPM_ (Δϕ_exp_) and Δ*t*_CPM_ (Δ*t*_exp_) are the potential difference and the time in MD simulation (experiment). (b) Total ion mass density, ρ_m_, and the number densities of cations and anions, ρ_ion_, inside the nanopores as a function of surface charge density, σ, in the galvanostatic charge–discharge process. The solid lines show the discharging process and the dashed lines the charging process. Reproduced with permission from ref (376). Copyright 2021 Nature Springer under CC-BY (http://creativecommons.org/licenses/by/4.0/).

Based on CPMs with fluctuating charges on electrode atoms,^124,259,294,377^ a CPM without limitation on electrode geometry was developed by Zeng et al.^376^ to simulate the galvanostatic charge–discharge (GCD) process of electrochemical devices in current mode, named GCD-CPM. In the GCD-CPM modeling, the sum of the electrode atom charges within an electrode is constrained to adjust the applied current. GCD-CPM was applied to investigate the GCD processes of ionic liquids in both open and nanoporous electrodes, and a comprehensive comparison with the CCM-based simulations has been made.^376^ For a flat electrode, the GCD curves and dynamic EDL structures obtained by the two methods agree well. For a nanoporous electrode, there are nonphysical phenomena in the constant-charge-based GCD simulations, such as a parabolic potential distribution on the electrode, excessive ion diffusion, and overmuch heat generation.^376^ In contrast, GCD-CPM captures the charging and discharging processes of nanoporous system consistent with experiment (Figure 34a), through the diffusion-type equation of the mean-field model^112^ discussed in section 4.5.1. The GCD-CPM simulations also revealed a “hysteresis” of ion adsorption–desorption dynamics during charging and discharging (Figure 34b), in which the ion density inside the pore during charging is different from the ion density during discharging. This effect originates from the slower response of electrolyte ions to polarization compared to the faster response of the electrode charges and the different in-pore ion responses during the charging and discharging.^376^

#### 6.4.7 Electrotunable Friction

Electrotunable lubrication (EL) is a fairly new field of research,^378^ which aims to control friction using external electrostatic fields. Most works to date have focused on studying friction in nanoscale RTIL films.^209,226,233,241,242,379^ Some attempts to investigate EL in thick aqueous solutions were reported recently, too.^236^ The great majority of EL computations focused on unravelling the microscopic mechanisms of electrotunable friction in RTILs by computing the friction force as a function of surface charge density, which can be controlled by setting up a potential difference between two confining surfaces with respect to a reference electrode (see ref (378)).

Many simulation studies have reported a linear dependence of the friction force with the normal load, *F*_L_, following the Amontons–Derjaguin’s law, *F* = *F*_0_ + μ*F*_L_ where μ = d*F*/d*F*_L_ is the friction coefficient and the cutoff *F*_0_ > 0 is determined by the adhesion force. The surface charge introduces minor changes to this general behavior, exhibiting a significant contribution of adhesion forces at both charged and neutral surfaces. The simulated friction coefficient (for sliding velocities of the order of 1–10 m/s) varies typically between μ = 0.01 and 0.1 in RTIL confined between silica, mica, amorphous carbon, iron, or gold surfaces.^225,233,242,380^ The magnitude of the friction coefficients agrees with experimental measurements.^324,381−383^ The friction coefficient varies significantly with film thickness. Experimental studies demonstrated the so-called “quantized friction” effect, a discrete multivalued friction behavior as a function of the normal load and the number of the confined ionic layers.^323^

**Figure 35:**
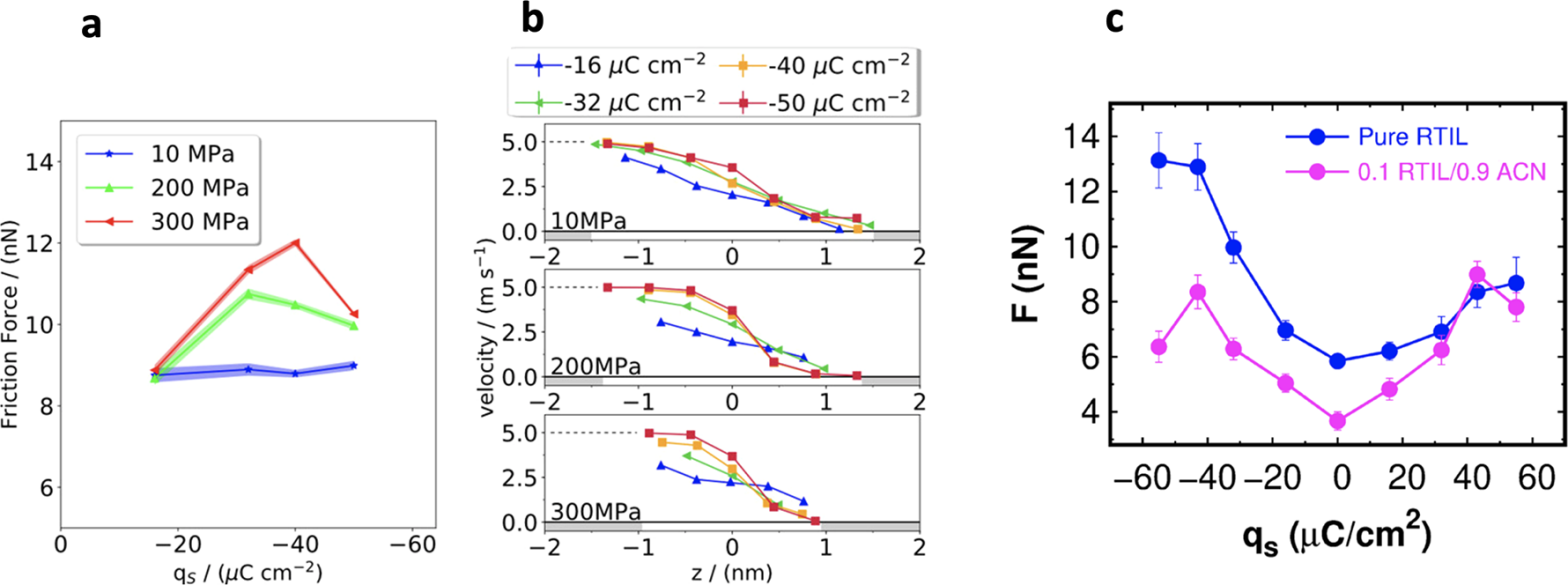
Friction forces and velocity profiles of RTILs under nanoconfinement. (a) Friction force of [C_6_MIM] [BF_4_] confined between negatively charged surfaces, as a function of the surface charge and normal load. (b) Velocity profiles of the same ionic liquid (as in (a)) as a function of surface charge and load. (a,b) Reproduced with permission from ref (233). Copyright 2020 American Chemical Society. (c) Friction force of [BMIM][ PF_6_] (pure and diluted at 0.1:0.9 molar fraction) as a function of surface charge, and a load of ∼500 MPa. Reproduced with permission from ref (386). Copyright 2020 American Chemical Society.

The friction force changes in a complex way with surface charge (Figure 35a). At low loads (10 MPa), it increases slightly with surface charge, while it increases significantly at high loads (100s MPa) before reaching a maximum and then decreasing upon further charging. This frictional behavior is coupled to the variation of velocity profiles in the confined films with surface charge and load. At low loads, when the films are relatively thick, the profiles conform to a typical Couette flow (see Figure 35b) with the velocity profile inside the fluid changing linearly between the velocity of the upper and the lower slabs (see, e.g., the results for −16 μC/cm^2^ and 10 MPa load in Figure 35b). However, at high loads and low surface charge, the dynamics of the film is very different. The compression induces the reduction of the film thickness, and the ionic layers feature a plug flow profile, with all the ionic layers moving at approximately the same speed. The increase in the surface charge leads to the strong adsorption of the ionic layers, modifying eventually this plug profile, which becomes of Couette type. Accordingly, the slippage plane is located between the strongly adsorbed RTIL layers and fluid layers in the interior of the film because the velocity of the adsorbed ionic layers is the same as that of the confining slabs (Figure 35b). The maximum in the friction force signals the *Plug-Couette flow dynamical transition*. At surfaces charges higher than the one corresponding to the transition, the friction forces decreases and the confined RTILs become better lubricants.

The EL mechanism discussed above has been predicted using primitive, coarse-grained, and fully atomistic models, crystalline or disordered lubricating films, and smooth and rough substrates.^226,233,242^ Low friction μ ∼ 0.001, which qualify as superlubricity states according to accepted definitions,^384^ were reported for surface charges above the Plug–Couette dynamic transition. Recent friction atomic microscope measurements support the observation of a maximum in the friction forces upon polarizing metallic surfaces.^385^ The simulations demonstrated that lubrication by RTILs relies upon two crucial properties, which are unusual in their combination: (1) counterions are attached firmly to the surfaces through their electrostatic interactions with charged surfaces, and (2) yet the film retains fluidity under compression. This combination of thermodynamic and kinetic features is essential to good boundary lubrication.

**Figure 36:**
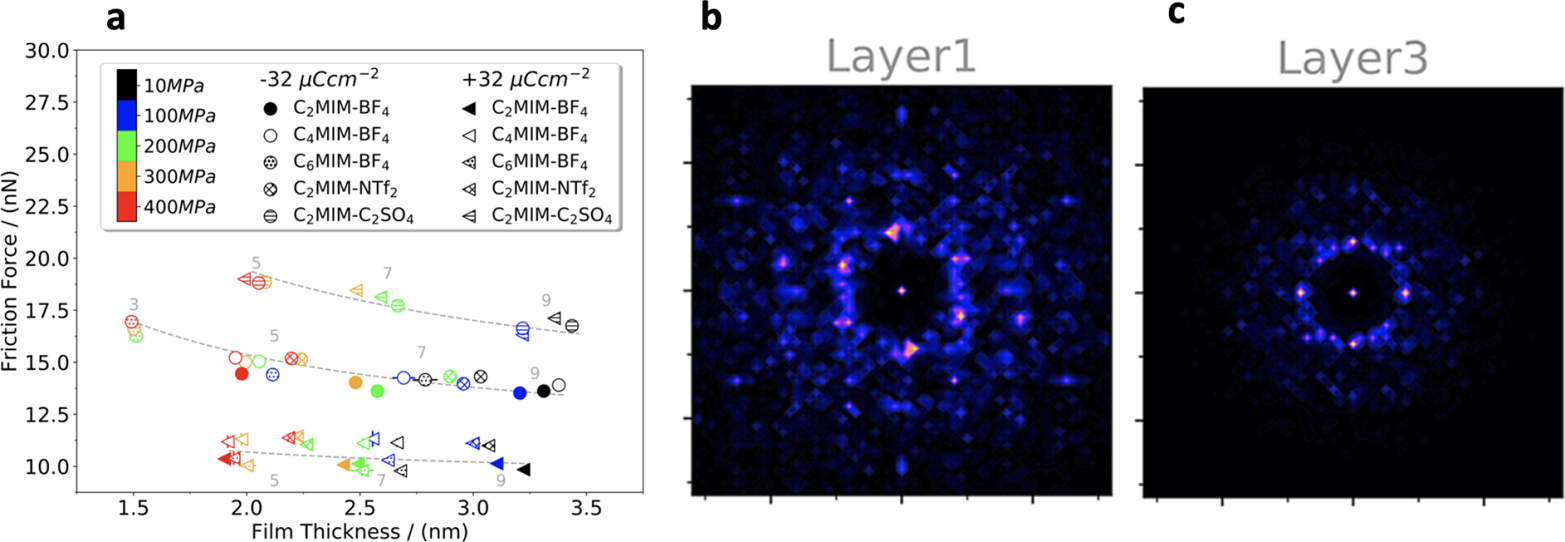
Dependence of the friction force with film thickness and interplane structure. (a) General dependence of the friction force of imidazolium ionic liquids with film thickness, polarity of the surface, size of the imidazolium cations, and normal load. (b) 2D structure factors of [C_2_MIM][BF_4_] confined between charged surfaces with the surface charge density σ = +32 μC/cm^2^. Layer 1 and layer 3 refer to the structure of the ionic liquid layer in contact with the surface and in the middle of the nanofilm, respectively. Left and right panels reproduced with permission from ref (234). Copyright 2020 American Chemical Society.

The interplay of RTIL composition and the polarity of the surface (positive or negative electrodes) has a significant impact on the friction force (Figure 36). Small cations ([EMIM]^+^) feature a better lubrication performance: lower friction forces and higher resistance to squeezing out than cations with long hydrocarbon chains. Enhanced lubrication was observed when RTILs were confined between positively charged surfaces, i.e., when the surface contact layers were enriched in anions. The structure and chemical composition of the anion in a RTIL plays a critical role in enhancing lubricity. In [EMIM] RTILs, [C_2_SO_4_]^–^ anions give rise to friction forces 60% higher than [BF_4_]^–^ and [NTF_2_]^–^ anions.^234^ This surprising result is connected to the in-plane ordering of the [EMIM][BF_4_]^–^ and [EMIM] [NTF_2_]^–^ nanofilms. Furthermore, crystallization triggers a friction mechanism similar to structural superlubricity,^387^ where the enhanced lubrication is due to the lattice misfit between the substrate and the RTIL crystal.

#### 6.4.8 Electrotunable Friction with Mixtures of RTILs and Polar Solvents

The study of RTILs diluted in polar solvents is receiving significant attention. The motivation is two-fold. First, many RTILs are hygroscopic and absorb water from humid air. Second, the addition of small amounts of IL in a solvent provides a route to reduce the cost of using RTIL lubricants in EL. Experiments of [EMIM][EtSO_4_] reported an increase in the film compressibility and the friction coefficient with water content.^388,389^ GC-MEMD simulations of [BMIM][PF_6_] show that water molecules adsorb at the charged surfaces, screening the repulsive Coulombic interactions between like-charged ions, and hence making the film softer, increasing its compressibility.^390^ Water adsorption shifts the slippage plane from the interior of the nanofilm to the solid–liquid interfaces, and the friction force increases, having important implications on the tribological properties of the system. Both CG and atomistic models showed that in aqueous mixtures of RTILs, water adsorbs strongly at mica surface, displacing the ions from the surface at sufficiently high water content.^214,391^

Diluted mixtures of RTILs with organic solvents (e.g., [BMIM][PF_6_] in acetonitrile) also feature a remarkable variation of the friction force with the electrode surface charge,^386^ which emerges from the accumulation of counterions at charged surfaces. Notably, the EL performance of diluted RTIL solutions is similar to that observed in pure RTIL. The ion-rich layers adjacent to charged plates are not squeezed out even under very high normal pressures (exceeding 100s of MPa), protecting surfaces from wear and providing lubrication at high loads.^321^ This significant result provides an approach to designing cost-effective RTILs for nanotribological applications.

## 7 Concluding Remarks

We have reviewed and discussed the current status of our understanding of the properties of ionic liquids with and without polar solvent additives in confined geometries with electrified “walls”, either polarizable or charged. We have overviewed the key concepts, different classes of physical models, and the existing theories of such systems. Our focus was on the effects specific to nanoconfinement. Thus, we limited the discussion of the bulk ionic properties and electrical double-layers at flat interfaces to the pure necessity (see refs (36), (37), (392), and (393), for a few reviews). We considered the associated phenomena in supercapacitors with nanostructured electrodes, energy storage, and charging dynamics and briefly sketched surface forces and nanoscale friction (for a recent detailed review of nanotribology with ionic liquid lubricants, see ref (378)).

An important part of the review was devoted to mapping the models of confined ILs onto the known statistical mechanics models broadly used in theory of adsorption and magnetism, such as Ising, Blume–Emery–Griffith, and Blume–Capel models on suitable lattices −1D, 2D, and Bethe lattices (sections 4.1.1 and 4.2.2). We also discussed an off-lattice approach and how it compares with the lattice models (section 4.1.2) and overviewed continuum density functional theory applied to confined ILs (section 5). We discussed these approaches in comparison with, where available, molecular simulations (section 6), as mapping the systems of this complexity on idealized, elementary models cannot be taken seriously without testing them against computer simulations or experiments. Simulations can handle more realistic representations of constituent ions and molecules and help identify which theoretical predictions are generic and which are system or model specific.

Focusing on the properties of ionic liquids, we only minimally discussed the properties of electrodes, their possible complex porous structure, finite density of states, and the associated quantum capacitance. These effects must be considered when the confinement is due to electrodes based on low-dimensional materials, such as graphenes, MXenes, CNTs, etc. The limited densities of electronic states give rise to quantum capacitance, which is generally necessary to consider in evaluating the energy storage capability of such electrodes but has not been discussed in the context of confinement so far.

Wherever possible, we attempted to outline the generic, model-independent or “less model-dependent” laws and distinguish them from system-specific effects. We discussed, where available, the comparison of the theoretical and simulation results with experiments and indicated which theoretical predictions would be interesting to test. Most currently available experimental techniques are based on macroscopic measurements related to capacitance, charging dynamics, or friction vs load measurements. A challenge for experiments remains the resolving of the structure of ionic liquids inside nanopores, where theory and in part simulations (Figures 6 and 7 and section 5.2.6) predict a variety of structural phase transformations not yet validated experimentally.

Despite its utmost importance, the ion dynamics in strong confinements remain largely unexplored, experimentally and theoretically. The available experimental data differ qualitatively from simulations, which *per se* show inconsistencies (section 6.4.1 and Figure 29). So far, limited information about the in-pore ion structure and dynamics is available experimentally. The essential steps have already been taken, however, particularly with NMR spectroscopy.^39,362,394−397^ We are excited to see new experimental advances either validating the existing theoretical predictions or posing new challenges to theory and simulations.

We feel that many scientifically intriguing and practically relevant problems are still ahead of us in this field. Future experiments will show how well the discussed theories and simulations capture reality, which essential features they miss, and how to improve them to fully align theory, simulations, and experiments, potentially leading to new discoveries.
