# Correction to “Theory and Simulations of Ionic Liquids in Nanoconfinement”

**DOI:** 10.1021/acs.chemrev.4c00885

**Published:** 2024-12-18

**Authors:** Svyatoslav Kondrat, Guang Feng, Fernando Bresme, Michael Urbakh, Alexei A. Kornyshev

In the original article, there is a typo in eq 9, which is missing a prime symbol next to the summation. The prime symbol indicates that the summation in this equation runs only over odd integer numbers. Thus, the correct version of this equation is
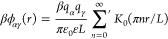
9Here β = (*k*_B_*T*)^−1^, *k*_B_ is the Boltzmann constant and *T* temperature, *q*_α_ and *q*_γ_ denote the ion charges, *K*_0_(*x*) is the zero-order modified Bessel function of the second kind, *L* is the slit width, ε is the relative permittivity, and ε_0_ is the permittivity of vacuum. Equation 9 can also be rewritten in an explicit form as
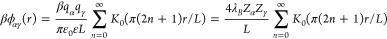
where λ_B_ = *βe*^2^/(4*πε*_0_ε) is the Bjerrum length, *Z*_α_ = *q*_α_/*e* and *Z*_γ_ = *q*_γ_/*e* are ion valencies, and *e* is the proton charge. The correct expression was used to produce Figure 2 in the original article.

We recall that this equation describes the interaction energy between two ions located at the symmetry plane of a slit. The complete expression for arbitrary ion positions in slit pores can be found in ref (1). We additionally stress that this interaction energy converges to the Coulomb interaction energy in the limit of the distance between the charges *r* → 0 (*r*/*L* ≪ 1).
